# Evolutionary drivers of encephalization and facial reduction in the genus *Homo*

**DOI:** 10.1038/s41467-026-74739-w

**Published:** 2026-07-06

**Authors:** Mark Hubbe, Katerina Harvati

**Affiliations:** 1 University of Tennessee - Knoxville, Department of Anthropology, Knoxville, USA; 2 DFG Center for Advanced Studies ‘Words, Bones, Genes, Tools: Tracking linguistic, cultural and biological trajectories of the human past’, Eberhard Karls University of Tübingen, Tübingen, Germany; 3 Paleoanthropology, Institute for Archaeological Sciences, Department of Geosciences, Eberhard Karls University of Tübingen, Tübingen, Germany; 4 Senckenberg Centre for Human Evolution and Palaeoenvironment, Department of Geosciences, Eberhard Karls University of Tübingen, Tübingen, Germany; 5 HUMAN ORIGINS—Cluster of Excellence for Integrative Human Origins Studies (EXC 3101), Eberhard Karls University of Tübingen, Tübingen, Germany

**Keywords:** Biological anthropology, Palaeontology

## Abstract

The evolution of cranial morphology in the genus *Homo* is marked by increasing encephalization and reduced facial robusticity through time. These changes are often assumed to be the product of directional natural selection for larger brain size, presumed to be associated with greater cognitive abilities, and for decreasing facial size to lower energetic costs, as masticatory demands were reduced through food processing and tool use. Here, we test this hypothesis by exploring the goodness-of-fit of the predictions of six evolutionary processes to the observed cranial morphological variation in *Homo*. We analyzed comprehensive neurocranial and facial 3D landmark coordinate datasets, grouped into eight Operational Taxonomic Units starting with early *Homo* and representing two lineages, *Homo sapiens* and *Homo neanderthalensis*. Our neurocranial analysis found strongest support for models of neutral evolution (Unbiased Random Walk) and stasis across both lineages. Similarly, the face shows strongest support for models of stasis, strict stasis, and neutral evolution (Unbiased Random Walk). Our findings suggest a limited role for gradual directional selection and underscore the importance of stabilizing selection and constraints in our lineage’s evolution, highlighting the importance of environmental constraints and possibly cultural behaviors as core drivers of human evolution.

## Abstract (web summary)

This study tests the evolutionary processes shaping the cranial morphology of the genus *Homo*. The authors report a limited role of gradual directional selection and underscore the importance of stabilizing selection and constraints in our lineage’s evolution.

## Introduction

The appearance of the genus *Homo* around 2.5–3.0 million years ago marks the beginning of a derived evolutionary trajectory for hominins, which resulted in multiple species with increased ability to occupy new habitats and ecological niches. With a few noteworthy exceptions (*Homo florensiensis*^1^; *Homo naledi*^2^), the evolution of our genus is marked by a general tendency towards increased encephalization^3,4^, reduced facial and dental robusticity^5,6^, and increased locomotion efficiency^7,8^. These trends culminated in the Middle to Late Pleistocene^9^ and were accompanied by important behavioral changes, including increased reliance on habitual stone tool use^10^, a related diversification of subsistence and food processing^11,12^, geographic range expansions^13,14^, and presumed changes in social structure^15,16^. It is generally assumed that these crucial behavioral changes were enabled over time by gradual directional selection for greater cognitive abilities, presumed to correlate with increased encephalization^17,18^. At the same time, cultural practices, such as the use of tools to process food, outsource biological activities to technological innovations and can buffer biological traits from selective pressures. These processes have been hypothesized as strong, gradual directional selection pressures for decreasing facial and dental size to reduce energetic costs^18,19^. We use a novel modeling method^20,21^ and a comprehensive dataset of fossil and recent humans to explore the fit of different evolutionary processes—gradual directional selection, stabilizing selection, neutral evolution, evolution towards an optimal value, punctuated equilibrium—to cranial morphological variation within the genus *Homo*. This approach translates evolutionary scenarios into quantifiable models, whose predictions can be compared to phenotypic observations from evolutionary lineages, allowing to test each model’s relative goodness-of-fit. We aim to test the commonly assumed hypothesis that gradual directional selection drove increased encephalization and reduced facial size of the genus *Homo* through time. Here, we show that gradual directional selection likely played a limited role in the evolution of the genus *Homo*’s cranial morphology and underscore the importance of stabilizing selection and constraints in our lineage’s evolution.

Our sample includes 63 fossil *Homo* and 24 recent *Homo sapiens* crania, grouped into eight Operational Taxonomic Units (OTUs) (Table S1): Early *Homo* (EH), including specimens attributed to *Homo habilis* and *Homo rudolfensis*; *Homo erectus s.l*. (HE); *Homo heidelbergensis s.l*. (HH); early *H. neanderthalensis* (EHN), comprising specimens dating to Marine Isotope Stage (MIS) 5 and older; classic *H. neanderthalensis* (LHN), comprising individuals younger than MIS5; early *H. sapiens* (EHS), comprising individuals dated to MIS5 or older; Upper Paleolithic *H. sapiens* (UPHS), including specimens from Europe, Asia, and Africa dating to MIS3; and recent *H. sapiens* (RHS), representing a broad geographical sample from recent/Holocene periods. We collected the data in the form of three-dimensional coordinates of osteometric landmarks and subdivided them into a neurocranial and a facial dataset (Tables S2 and S3^22–25^). These datasets represent the anatomical regions associated with changes derived from encephalization and facial reduction trends in the genus *Homo*. Encephalization and facial reduction are best described as changes relative to overall body size, and as such are difficult to evaluate in the fossil record, given that body size is unknown or poorly estimated for most hominin fossils. To address this limitation, we assess trends in morphological change focusing on the relative shape changes in each anatomical region, as well as on the analysis of the overall size of the specimens. As such, we assume that, even though our morphological data do not measure directly encephalization and facial reduction relative to body size, they are valid proxies for these processes in the genus *Homo*, which are associated with significant and well-documented changes in the overall architecture of the cranium (absolute expansion of the brain, increases in relative cranial height and expansion of relative cranial breadth; reduction of lower facial prognathism, overall verticalization of the face).

We process landmark data using Procrustes Superimposition to remove differences due to rotation, translation, and size among specimens, in order to best reflect the morphological trends investigated here (relative changes in neurocranial and facial shape). We conduct Principal Components Analyses (PCA) on the superimposed coordinates, and the resulting PC Scores (PCs 1–4 for each analysis) are used as phenotypic variables that represent major axes of morphological variation in the neurocranium and the face. We also analyze the centroid sizes extracted from the General Procrustes Analysis of each dataset to evaluate evolutionary models that best support the changes in overall size along the genus *Homo*. For each anatomical region, we test the fit of six evolutionary scenarios: General Random Walk (GRW) representing gradual directional selection; Unbiased Random Walk (URW) representing neutral evolutionary processes; Evolutionary Stasis (ES) interpreted as stabilizing selection; Strict Stasis (StS) representing strong stabilizing selection; Ornstein-Uhlenbeck (OU), interpreted as directional selection towards an adaptive peak; and Punctuated Equilibrium (PE), interpreted as fast directional selection separated by long periods of stabilizing selection.

The model-testing approach we use requires ancestral-descendant relationships between OTUs and can only accommodate a single evolutionary lineage at a time. We therefore run the analyses twice, once for each of the two commonly recognized later *Homo* lineages, *H. neanderthalensis* and *H. sapiens*^9,23,26–28^, with *H. heidelbergenis s.l*. representing their last common ancestor (but see^9,29^ for alternative phylogenetic scenarios).

## Results

### Encephalization

**Fig. 1:**
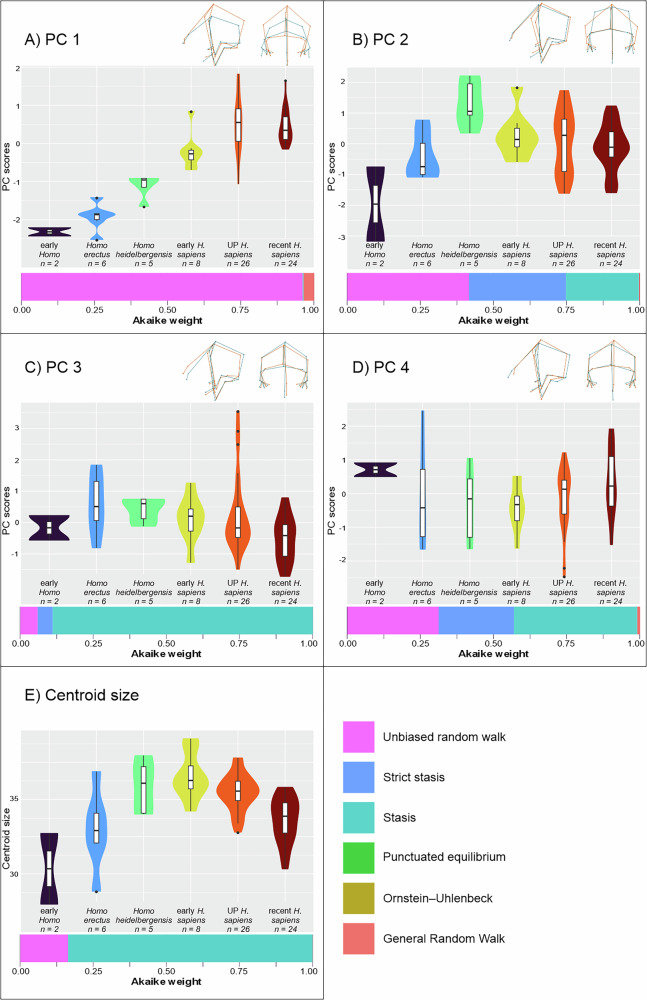
Neurocranial morphological variation across OTUs in the *Homo sapiens* lineage showing the morphological trends represented in the first four Principal Components and the centroid size, and their fit to the evolutionary models tested based on Akaike weights. The wireframes in each plot illustrate the morphology represented by the lowest PC score (blue lines) superimposed with the morphology for the highest PC score (orange lines). Akaike weight values represented in the bars below the plots reflect the values reported in Table 1. Data distribution is presented as violin plots showing relative density of points and as boxplots marking the median, interquartile range (IQR, white box), and lines defined by maxima/minima or 1.5 times IQR. **A** Morphological change and Akaike weights based on Principal Component 1. **B** Morphological change and Akaike weights based on Principal Component 2. **C** Morphological change and Akaike weights based on Principal Component 3. **D** Morphological change and Akaike weights based on Principal Component 4. **E** Morphological change and Akaike weights based on centroid size.

The PCA of the neurocranial dataset for the *H. sapiens* lineage captured in the first two PCs (Fig. 1) changes in neurocranial shape that are well-described for the genus *Homo*. Table S5 presents the goodness of fit based on log-likelihoods of the six models to each PC. The Akaike weights, which take into account the model complexity, are used here to evaluate the relative support of each model, and they are reported in Table S5 and summarized in Table 1. PC1 (27.5% of variance; Table S4) reflected overall neurocranial expansion in relative cranial height, especially at bregma, as well as in cranial breadth, occurring in a relatively linear trend through time. For this PC, our analysis found the Akaike weights lending overwhelming support for unbiased random walk (URW: 0.961), with very low probability of support for general random walk (GRW: 0.036) (Tables 1, S5). In contrast, PC2 (14.1% of variance; Table S4) reflected relative expansion in parietal breadth in *H. heidelbergensis* compared mainly to early *Homo* and *H. erectus* (Fig. 1). Here, the Akaike weights show that the best supported evolutionary model was unbiased random walk (URW 0.416), followed closely by strict statis (StS: 0.331), and evolutionary stasis (ES: 0.249) (Tables 1, S5). Evolutionary stasis (ES) was the strongest model for PC3 (7.3%; Table S4) and PC4 (6.3% of variance; Table S4) with Akaike weights of 0.889 and 0.421, respectively (Fig. 1, Table 1, S5). The changes in centroid size over the lineage (Fig. 1) show the well-documented trend of neurocranial size increase from early *Homo* to *Homo erectus* to *H. heidelbergensis*, followed by a size decrease in later and recent *Homo sapiens*. The Akaike weights show very strong support for evolutionary stasis (ES: 0.835) followed by unbiased random walk (URW: 0.163). The general trends reported here were observed across several alternative *Homo sapiens* lineages tested, detailed in supplementary results through Tables S7–S10.

**Table 1:** Weighted Akaike Scores for each of the evolutionary models tested against the Principal Component scores and Centroid Sizes of the four datasets analyzed

	General Random Walk	Unbiased Random Walk	Stasis	Strict Stasis	Punctuated Equilibrium	Ornstein-Uhlenbeck
	Neurocranial Analysis - *Homo sapiens* lineage					
PC1	0.036	**0.961**	0.003	0	0	0
PC2	0.003	**0.416**	0.249	0.331	0	0
PC3	0	0.059	**0.889**	0.051	0	0
PC4	0.01	0.312	**0.421**	0.257	0	0
Centroid Size	0.001	0.163	**0.835**	0	0	0
	Neurocranial Analysis - *Homo neanderthalensis* lineage					
PC1	0.024	**0.931**	0.045	0	0	0
PC2	0	0.026	0.15	**0.824**	0	0
PC3	0	0.03	0.054	**0.916**	0	0
PC4	0	0.017	0.184	**0.799**	0	0
Centroid Size	0	0.064	0.053	**0.883**	0	0
	Facial Analysis - *Homo sapiens* lineage					
PC1	0.041	0.387	**0.571**	0	0	0
PC2	0.001	0.062	0.149	**0.788**	0	0
PC3	0.005	0.434	**0.561**	0	0	0
PC4	0.003	**0.461**	0.441	0.096	0	0
Centroid Size	0.003	0.477	**0.519**	0	0	0
	Facial Analysis - *Homo neanderthalensis* lineage					
PC1	0	0.032	0.033	**0.934**	0	0
PC2	0.006	**0.951**	0.043	0	0	0
PC3	0	0.032	0.053	**0.915**	0	0
PC4	0	0.267	**0.398**	0.336	0	0
Centroid Size	0	0.033	0.033	**0.934**	0	0

In **bold**: Best supported model in each analysis.

**Fig. 2:**
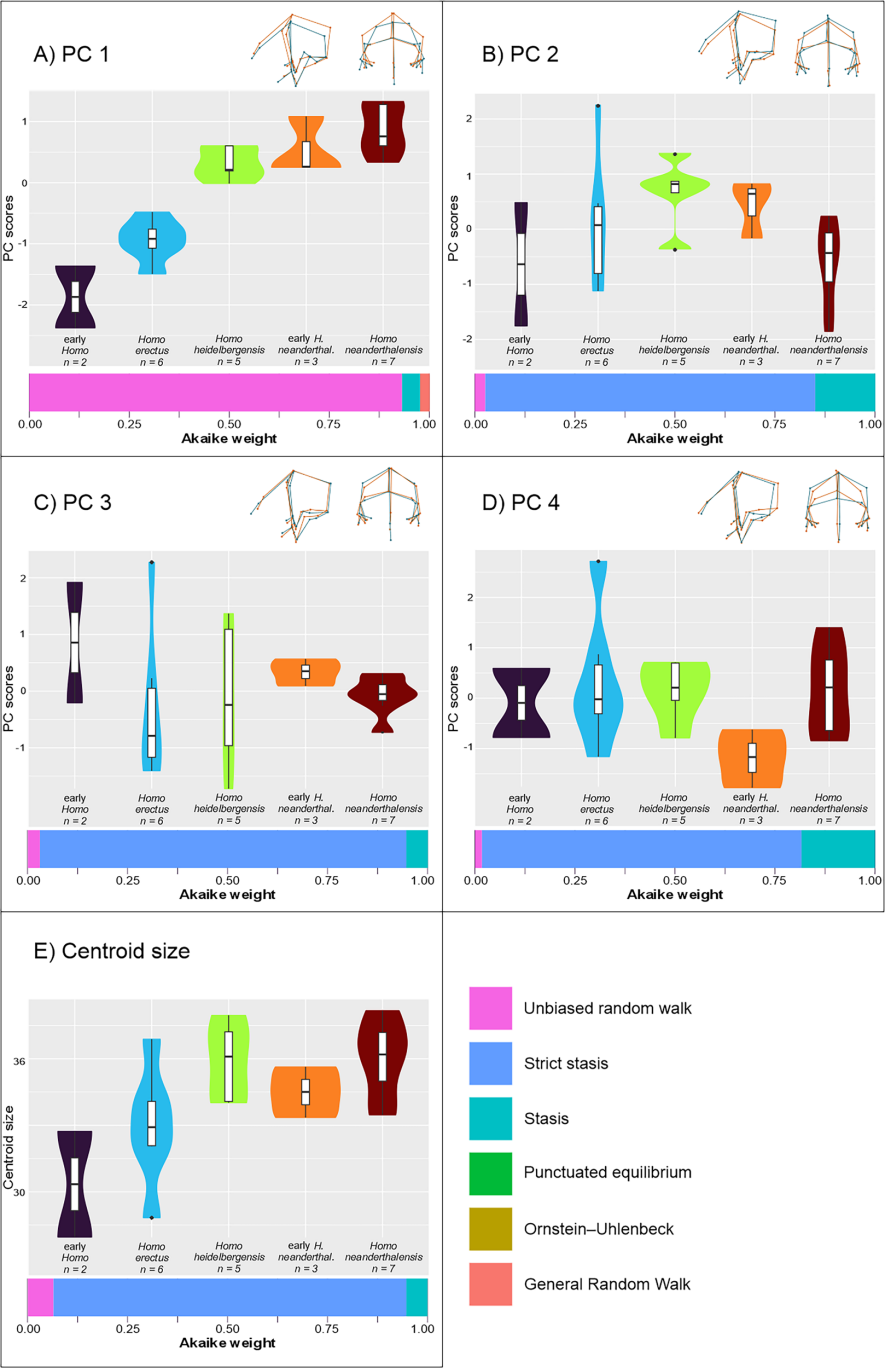
Neurocranial morphological variation across OTUs in the *Homo neanderthalensis* lineage showing the morphological trends represented in the first four Principal Components and the centroid size, and their fit to the evolutionary models tested based on Akaike weights. The wireframes in each plot illustrate the morphology represented by the lowest PC score (blue lines) superimposed with the morphology for the highest PC score (orange lines). Akaike weight values represented in the bars below the plots reflect the values reported in Table 1. Data distribution is presented as violin plots showing relative density of points and as boxplots marking the median, interquartile range (IQR, white box), and lines defined by maxima/minima or 1.5 times IQR. **A** Morphological change and Akaike weights based on Principal Component 1. **B** Morphological change and Akaike weights based on Principal Component 2. **C** Morphological change and Akaike weights based on Principal Component 3. **D** Morphological change and Akaike weights based on Principal Component 4. **E** Morphological change and Akaike weights based on the centroid size.

For the *H. neanderthalensis* lineage PCA, shape changes related to encephalization were captured mainly by PC1 (33.9% of the variance; Table S4), which reflected differences in relative neurocranial height, especially at lambda, as well as in relative breadth and length. These changes occurred in a relatively linear fashion through time, but with a clear gap between *H. neanderthalensis* and *H. heidelbergensis s.l*. on the one hand; and early *Homo* and *H. erectus s.l*. on the other (Fig. 2). Test of goodness of fit results were similar to those of the *H. sapiens* lineage for PC1, with the Akaike weights lending strongest support for unbiased random walk (URW: 0.931), and low probabilities for evolutionary stasis (ES: 0.045) and general random walk (GRW: 0.024) (Tables 1, S5). For PC2 (14.1%; Table S4), PC3 (8.8%; Table S4), and PC4 (8.3%; Table S4), the Akaike weights show the strongest support for strict stasis (StS, PC2: 0.824, PC3: 0.916, PC4: 0.799; Fig. 2; Table 1, S5). The analysis of the centroid sizes reflects documented changes in size in the lineage. Similar to PCs 3 and 4 in this analysis, the Akaike weights show strong support for strict stasis (StS: 0.883), and low levels of support for unbiased random walk (URW: 0.064) and evolutionary stasis (ES: 0.053; Tables 1, S5). Alternative scenarios tested for the *H. neanderthalensis* lineage (removing early *Homo*; pooling all *H. neanderthalesis* into a single OTU) show that the removal of these OTUs greatly affects the results. In these cases, the models that are best supported by Akaike weights (Tables S7, S11) are Ornstein-Uhlenbeck (OU) and Punctuated Equilibrium (PE). However, the alternative analyses that include only *H. habilis* or only *H. rudolfensis* in the early *Homo* OTU (Tables S8, S9) show similar results as the ones presented here.

### Facial reduction

**Fig. 3:**
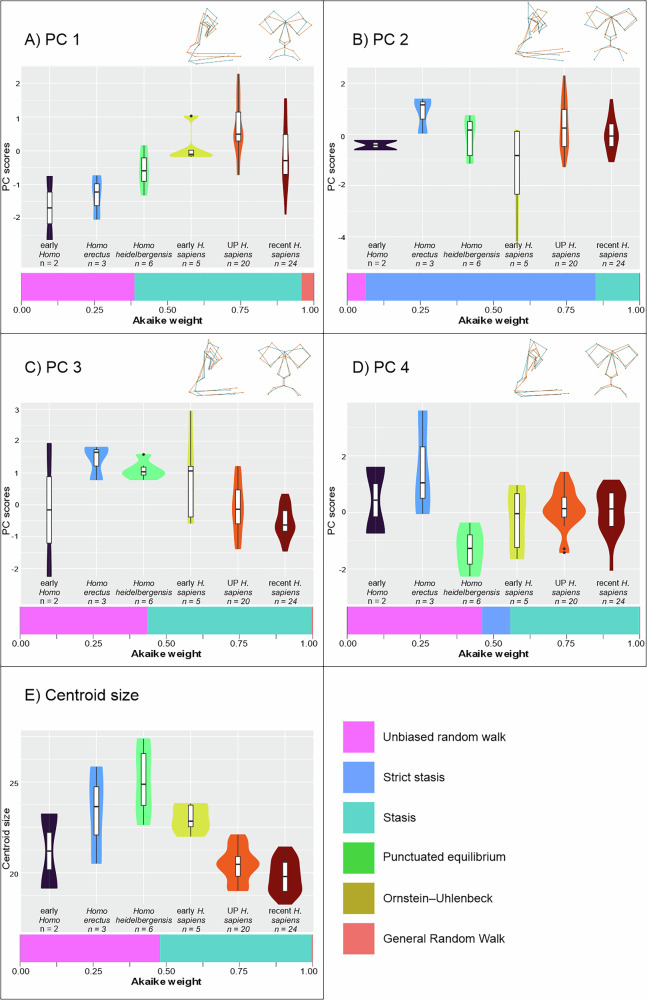
Facial morphological variation across OTUs in the *Homo sapiens* lineage showing the morphological trends represented in the first four Principal Components and the centroid size, and their fit to the evolutionary models tested based on Akaike weights. The wireframes in each plot illustrate the morphology represented by the lowest PC score (blue lines) superimposed with the morphology for the highest PC score (orange lines). Akaike weight values represented in the bars below the plots reflect the values reported in Table 1. Data distribution is presented as violin plots showing relative density of points and as boxplots marking the median, interquartile range (IQR, white box), and lines defined by maxima/minima or 1.5 times IQR. **A** Morphological change and Akaike weights based on Principal Component 1. **B** Morphological change and Akaike weights based on Principal Component 2. **C** Morphological change and Akaike weights based on Principal Component 3. **D** Morphological change and Akaike weights based on Principal Component 4. **E** Morphological change and Akaike weights based on the centroid size.

The PCA of the facial dataset (Fig. 3) for the *H. sapiens* lineage captured changes in facial shape related to facial reduction in the genus *Homo* in the first PC (22.1% of variance), occurring in a relatively linear fashion through time, but with a marked change between UP and recent *H. sapiens*. This axis reflected a reduction in overall relative prognathism and dental arcade length, a more vertical orientation of the face, and a relative narrowing of the upper face through time. The relative Akaike weights for this PC showed the strongest support for evolutionary stasis (ES: 0.571) followed by unbiased random walk (URW: 0.387), with lower levels of support for general random walk (GRW: 0.041; Tables 1, S5). Additionally, PC3 (10.7%; Table S5) reflected shape differences in lower face prognathism and upper face elongation between early *Homo* and *H. erectus s.l. / H. heidelbergensis s.l*. (Fig. 3). The Akaike weights for this PC showed broadly equivalent support for evolutionary stasis (ES: 0.561), and unbiased random walk (URW: 0.434). PC2 (11.6%; Table S4) does not show an easily discernible morphological change along the lineage, and the Akaike results present strongest support for strict stasis (StS, PC2: 0.788), followed by evolutionary stasis (ES: 0.149). PC4 (6.6%; Table S4) tracks the difference between early *Homo*/*H. erectus* and the later *Homo* OTUs in lower face prognathism. Here, the Akaike weights show the strongest support for unbiased random walk (URW: 0.461) and evolutionary stasis (ES: 0.441), and a lower level of support for strict stasis (StS: 0.096) (Fig. 3; Tables 1, S5). As with the neurocranial analyses, the results for centroid sizes show the well-described patterns of facial size in the lineage (Fig. 3), with an increase in overall size from early *Homo* to *H. heidelbergensis*, followed by the decrease in overall size in *H. sapiens*. The Akaike weights near-equally support evolutionary stasis (ES: 0.519) and unbiased random walk (URW: 0.477). Also similarly to the neurocranial analyses, the alternative scenarios tested show no significant departure from the results presented here (Tables S7–S10).

**Fig. 4:**
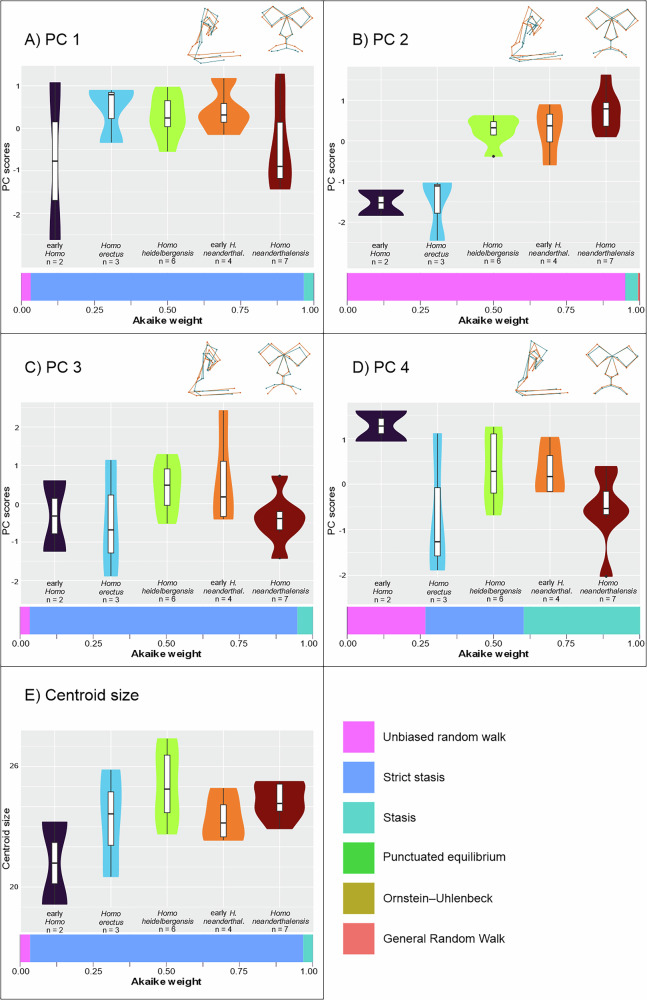
Facial morphological variation across OTUs in the *Homo neanderthalensis* lineage showing the morphological trends represented in the first four Principal Components and the centroid size, and their fit to the evolutionary models tested based on Akaike weights. The wireframes in each plot illustrate the morphology represented by the lowest PC score (blue lines) superimposed with the morphology for the highest PC score (orange lines). Akaike weight values represented in the bars below the plots reflect the values reported in Table 1. Data distribution is presented as violin plots showing relative density of points and as boxplots marking the median, interquartile range (IQR, white box), and lines defined by maxima/minima or 1.5 times IQR. **A** Morphological change and Akaike weights based on Principal Component 1. **B** Morphological change and Akaike weights based on Principal Component 2. **C** Morphological change and Akaike weights based on Principal Component 3. **D** Morphological change and Akaike weights based on Principal Component 4. **E** Morphological change and Akaike weights based on the centroid size.

The *H. neanderthalensis* lineage PCA shows the clearer differentiation in facial morphology along PC2 (11.6%, Table S4), associated with the progressive loss of lower face prognathism in the lineage. This axis of variation has the strongest support according to Akaike weight to unbiased random walk (URW: 0.951), with a lower level of support for evolutionary stasis (ES: 0.043) (Fig. 4; Tables 1, S5). The morphological patterns shown by the other PCs are less clear. PC1 (22.1% of variance; Table S4) and PC4 (6.6% of variance; Table S4) differentiate early *Homo* from the other taxa, while PC3 (10.6% of variance; Table S4) shows no clear pattern of change across the lineage. As with the neurocranial analysis for the *H. neanderthalensis* lineage, PC1 and PC3 have Akaike weights showing overwhelmingly support for strict stasis (StS, PC1: 0.934, PC3: 0.915). PC4 has Akaike weights supporting evolutionary stasis (ES: 0.398), strict statis (StS: 0.336), and unbiased random walk (URW: 0.267) (Fig. 4; Tables 1, S5). The analysis of the centroid sizes shows an overall increase in facial size from early *Homo* to *H. heidelbergensis*, followed by a small reduction in size among *H*. *neanderthalensis*. The log-likelihood values for the models tested are relatively similar for this variable, and the Akaike weights overwhelmingly support strict stasis (StS: 0.934; Tables 1, S4). The alternative scenarios tested for this dataset show the same patterns as observed in the neurocranial analysis: scenarios with reduced OTUs show stronger support for OU and PE (Tables S7, S11), while the changes in composition of the early *Homo* OTU do not have major effects on the results (Tables S8, S9).

## Discussion

Here we aimed to test the hypotheses that the observed trends for encephalization and for facial reduction in the evolution of *Homo* were driven by gradual directional selection. In the case of encephalization, we hypothesized directional selection for cognitive abilities, presumably related to larger brain size, which became increasingly important with growing reliance on technology and with greater social complexity through time. In the case of facial reduction, we hypothesized that directional selection drove this trend as a response to the gradually reduced need for masticatory power with an increased reliance on cultural practices for processing food, which, in turn, would make the energetic investment in large faces disadvantageous.

Our PCAs for both the face and the neurocranium captured shape differences among *Homo* species that are related to these temporal trends. In both cases, however, the trends were clearest for the *H. sapiens* lineage. In the neurocranium, the *H. sapiens* lineage PCA showed this trend on two axes: PC2 reflecting a shift in parietal breadth starting with *H. heidelbergensis s.l*. relative to the earlier taxa; and PC1, showing a more or less linear trend of increased cranial height and breadth, culminating in *H. sapiens*. In contrast, the *H. neanderthalensis* lineage PCA showed these changes only in one axis, PC1, which reflected increased cranial parietal breadth and height and showed both a change in *H. heidelbergensis s.l*. relative to earlier taxa, as well as a second, more subtle, shift starting with the late *H. neanderthalensis*. These findings are consistent with the differences in the cranial morphology of *H. sapiens* and *H. neanderthalensis*: the former exhibiting a derived reorganization of the neurocranium with a posteriorly rounded shape, while the latter accommodating increases in brain size within the ancestral, elongated cranial architectural plan^17^.

Although the encephalization and related morphological trends were shown clearly in our PCAs, our analysis of goodness of fit of different evolutionary scenarios did not support gradual directional selection as a driving force. Instead, for both lineages, variation along the relevant axes overwhelmingly fits a model of stochastic changes best, followed to a much smaller degree by stabilizing selection. Although directional selection was also detected in some of these analyses, its probability was minimal. Additionally, all other axes of variation investigated (PCs 3–4 in the *H. sapiens* lineage analysis, PCs 2–4 in the *H. neanderthalensis* lineage analysis) were found to fit best with stabilizing selection, in most cases strong stabilizing selection. The analyses of changes in size through time in the two lineages complement those of shape differences. Even though they also document well-described patterns of change along each lineage, they show stronger support for models of stasis in the *H. sapiens* lineage, and strict stasis in the *H. neanderthalensis* lineage. Although the process of encephalization in the genus *Homo* is a function of brain size expansion relative to body size, it is also true that neurocranial shape changes in both lineages are associated with changes in absolute cranial size. In this sense, our results indicate that neurocranial size itself is likely not under directional selective pressure along the *Homo* genus.

For the facial analysis, our PCAs again captured the morphological differences associated with facial reduction more clearly for the *H. sapiens* lineage. A relatively linear trend associated with reduction in prognathism and dental arcade length, more vertical orientation of the face, and relative narrower upper face was observed on PC1, while PC3 additionally reflected a reduction in lower face prognathism and relative elongation of the upper face from early *Homo* to *H. erectus*/*H. heidelbergensis s.l*. The patterns were weaker in the *H. neanderthalensis* lineage PCA, with PC2 capturing the general tendency of reduced prognathism, while PCs 1 and 4 reflected the significant change between early *Homo* and later taxa. Our results are consistent with the expected morphological differences between *H. sapiens* and *H. neanderthalensis*, with the latter generally retaining larger faces compared to the former.

Again, our goodness-of-fit analysis found little support for gradual directional selection driving these trends in either lineage. In the *H. sapiens* analysis, they were attributed mainly to stabilizing selection and, to a lesser degree, random processes, with minimal support for linear directional selection. In the *H. neanderthalensis* analysis, strong stabilizing selection was the best fit for variation along the first axis, and the second showed strong support for neutral evolutionary models. In both analyses, the departure of *H. erectus* from early *Homo* in the degree of lower face prognathism fit with neutral processes and stabilizing selection. Similar to neurocranial size, facial size was best explained by stasis and stochastic models in the *H. sapiens* lineage and by strict stasis for the *H. neanderthalensis* lineage. These results are consistent with our facial shape analysis and suggest that the overall size of the face was not subject to linear directional selection.

It is important to note here that the axes of variation most clearly reflecting the evolutionary trends described in our hypotheses (for *H. sapiens*, PCs 1 and 2 in the neurocranium and PCs 1 and 3 in the face; for *H. neanderthalensis*, PC 1 in the neurocranium, and PC2 in the face) were also the only ones where directional selection was indicated as a potential fit, albeit with minimal probability. In these axes of variation, the log likelihood for the gradual directional selection model would support better goodness of fit for these models if taken at face value (Table S5). However, the model comparisons based on Akaike weights penalize models that are more complex (i.e., models that require more statistical parameters) to prevent overfitting, and instead favor models that are generalizable. Because gradual directional selection is a model that is more complex than the stochastic and stasis models, it receives less support than alternative simpler models, if the latter are able to explain the data similarly well. Therefore, it is important to underline that, although there may be an expectation of directional selection when directional trends in morphology are observed in the fossil record, these trends are not strong enough to outweigh the additional complexity of a gradual directional selection process compared to differences that can be accumulated under neutral or constraining evolutionary models. Furthermore, we cannot rule out the possibility that linear directional selection played a role under more complex evolutionary scenarios, such as punctuated equilibrium models, where directional selection is followed by long periods of stasis, or Ornstein-Uhlenbeck models, where traits evolve towards and oscillate around adaptive peaks. Although our tests did not support the Punctuated Equilibrium or Ornstein-Uhlenbeck models for our datasets, it is possible that the small number of OTUs, which reflect the limitations of the hominin fossil record, preclude us from properly testing the fit to such complex scenarios. In other words, while we found no support for more complex models based on the data available, it is possible that we lack the data required to properly test these models. Therefore, we cannot exclude adaptive processes occurring in small portions of the lineage, or selective pressures that change in tandem with relatively fast environmental change over the evolution of our genus. Finally, our neurocranial dataset was based exclusively on ectocranial landmarks and as such its variation is partially influenced by cranial topography not directly related to brain size, like the mastoid process and glabellar prominence. While we assumed here that this source of variation will not be significant when compared to overall neurocranial form, our conclusions should be validated in the future using endocranial variables.

In conclusion, our results are consistent with previous work suggesting a limited role for gradual directional selection in human evolution^30–32^ and underscore the importance of stabilizing selection and constraints, which our analyses strongly supported relative to other evolutionary processes. The cranial morphology of the genus *Homo* was therefore likely shaped by factors constraining the accumulation of variation (i.e., that promoted evolutionary stasis), as well as by stochastic processes. The commonly proposed model of gradual selective pressures generating adaptive differences over time in human evolution—reflected in the hypotheses tested here—largely overlooks the importance of such constraints and stabilizing forces. Rather than focusing on directional selection, therefore, future work may more productively focus on the role that the release of stabilizing selection may have played in critical transitions in the history of the genus *Homo*. A reduction in the strength of selective constraints will result in an increase in variance within a lineage, and since the evolutionary potential of a lineage is positively associated with the amount of phenotypic variance, releases of stabilizing selection are likely associated with faster evolutionary rates. Therefore, the identification of key moments of release in selective constraints are of high importance to the understanding of the evolutionary trajectories of hominins. While the fossil record offers limited ability to explore this through an analysis of the increase in variance within hominin species, our observations that encephalization appears to be driven by a combination of neutral processes and/or stasis suggests that both the early stages of encephalization seen with *H. heidelbergenis s.l*. and the changes associated with the evolution of the large-brained *H. sapiens* and *H. neanderthalensis* were potentially related to a release of constraints. Such constraints could have included, in addition to ontogenetic and architectural factors, also energetic and metabolic ones, which would have been fundamentally altered with the increasing reliance of *Homo* species on cultural behaviors, such as the growing exploitation of animal resources, culminating in the top predator niche position of both *H. sapiens* and *H. neanderthalensis;* and the emergence of cooking, a process which often increases the nutritional value of food^33^. These same behavioral changes would be expected to also drastically affect the evolution of facial reduction. Here, too, gradual directional selection was not supported as an evolutionary driver in our results. Rather, as with encephalization, an important release of constraints is suggested in the transition from early *Homo* to *H. erectus s.l*., that may have been enabled by the increasing replacement of masticatory function with cultural practices. Additional changes associated with the *H. sapiens* lineage may be potentially attributed to similar factors, although these were not found to influence the evolution of *H. neanderthalensis’* facial morphology, which instead appeared to remain highly constrained. While our findings do not nullify the need for identifying selective pressures across our evolutionary trajectory, they shift the focus of attention to the conditions and mechanisms, including cultural behaviors, under which *Homo* populations were able to evade the evolutionary limits constraining their potential to evolve new phenotypes^18^.

## Methods

### Specimen inclusion

The analyses are based on a dataset of 63 fossil specimens assigned to the genus *Homo*, complemented by data from recent modern humans. Inclusion of fossils in the analyses depended on their state of completeness for each of the anatomical regions analyzed (face and neurocranium). The neurocranium dataset included 57 specimens and the face included 47. Table S1 lists the fossils included in the final analyses with their contextual information (OTU assigned, chronological age, and percentage of missing values estimated). 24 complete skulls of modern *Homo sapiens* were added to the fossil specimens, to represent modern humans. The 24 individuals represent one male and one female randomly chosen from a larger sample of 233 individuals from 12 modern human populations worldwide, representing subsets of previously published samples^22–25^ (Table S1). This sample size was chosen to avoid the overrepresentation of modern humans in the calculation of the average covariance between landmarks.

### Operational taxonomic units

To maximize the number of steps in the *Homo* evolutionary lineage in the tests of evolutionary scenarios, we classified the specimens into eight Operational Taxonomic Units (OTUs) along chronological and species boundaries (Table S6). In order to maximize specimens for each OTU we used the broadest definitions of these taxa (i.e., *Homo erectus* s.l., *Homo heidelbergensis* s.l., and early Homo)^10^. For *Homo neanderthalensis* and *Homo sapiens*, the chronological resolution available for the fossils and the larger sample sizes allowed them to be separated into more than one OTU to represent morphological trends over time. Table S6 presents the final breakdown of specimens per OTU for each of the datasets as well as their assumed chronological ranges. The OTUs represent different taxonomic levels, with some of them grouping fossils considered as being part of different species (early *Homo* includes *H. rudolphensis* and *H. habilis*) and other OTUs separating fossils of the same species into different chronological OTUs (early and late *H. neaderthalensis*, and early, Upper Paleolithic, and recent *H. sapiens*). This approach is consistent with the model-fitting methods adopted, as they do not require that similar taxonomic levels are represented, only that they are part of a lineage with relative chronology known for each of its steps. The models tested assume that the lineage was subject to the same evolutionary process across its entire existence, which means that the expected changes in trait values are proportional to the time that separates consecutive steps in the lineage. As such, OTUs chronologically close to each other are expected to show small changes compared to more distantly related steps. This proportional expectation of change is consistent with diachronic trends within a species as well as with changes between species. Parameters that are of larger relevance to the estimation of models’ goodness of fit are the mean and variance in each step and the number of steps in the lineage. Therefore, the lineages used in this study show a compromise between the bare minimum number of fossils in an OTU to allow a calculation of variance and the maximum number of OTUs that can be considered part of the lineage. However, the scarcity of the fossil record makes the statistical impact of our grouping decisions hard to evaluate. To address this limitation, we ran several alternative scenarios, with different combinations of OTUs or specimens in each OTU. All the analyses described in subsequent sections were replicated for the OTUs presented in Table S6 and for five alternative scenarios:

- Alternative scenario A (Table S7) removed the two early *Homo* specimens, and started each lineage with *H. erectus*, to explore the impact of not having the earliest OTU, given that it is defined by only one specimen for each of two species (*H. habilis* and *H. rudolphensis*).
- Alternative scenario B (Table S8) only includes the *H. habilis* specimen in the early *Homo* OTU, and assumes its measurements represent the average of the species. Variance of this OTU is defined as the average variance within all other OTUs.
- Alternative scenario C (Table S9) only includes the *H. rudolphensis* specimen in the early *Homo* OTU, and assumes its measurements represent the average of the species. As with the previous scenario, variance of this OTU is defined as the average variance within all other OTUs.
- Alternative Scenario D (Table S10) removed modern *H. sapiens* from the analyses, to make the results directly comparable to the results of the *H. neanderthalensis* lineage in terms of number of OTUs and approximate sample sizes.
- Alternative scenario E (Table S11) joined all *H. neanderthalensis* fossils in a single OTU, to respect the currently accepted species boundaries for the species.

### Morphometric data

Coordinate data were collected by KH using either a microscribe directly from specimens or digitally from 3D models. Landmarks were selected to represent overall craniofacial morphology, while minimizing missing information in the data (see below). The final datasets comprised 21 landmarks for the neurocranial and 23 landmarks for the facial datasets as subsets of previously published data^22–25^. Tables S2, S3 list the landmarks included in each dataset, as well as the percentage of missing data for each landmark.

Encephalization is best defined as the increase in cranial capacity relative to body size. As information on body size is not available for most specimens included in this study, we use changes in shape and changes in overall size as proxies to encephalization, given that encephalization in the genus *Homo* was accompanied by both significant changes in neurocranial architecture and in absolute size. Therefore, the shape analyses were conducted on a dataset scaled to size (see below) and our analysis of size was based on the calculation of the centroid size for each specimen. A similar approach was adopted for the analysis of facial reduction, as facial changes in the genus *Homo* are reflected in changes in shape and absolute size over the lineage.

Our neurocranial dataset is based on ectocranial landmarks (Table S2) and as such its variation is partially influenced by cranial osseous structures that are not directly related to brain size or shape. Nevertheless, we assume here that this variation is minimal and that the greatest component of the variance in neurocranial form is associated with brain size and shape, as the brain represents a large proportion of neurocranial volume in the genus *Homo*.

Estimation of missing data in the fossils was conducted in three steps. During data collection, when the morphological features allowed for accurate inference of landmarks missing, landmark coordinates were approximated. After data collection, bilateral landmarks were estimated by reflecting the side of available landmarks into the side of missing landmarks, following^34^. Finally, each dataset was trimmed to remove any specimen with more than 35% of landmarks missing. The trimmed datasets had their missing values estimated using thin-plate splines to interpolate landmarks missing^34^. The combination of these three steps of missing data estimation represents a necessary compromise to minimize the information missing in the original fossil record, while generating enough data points for the estimation of goodness-of-fit of the different evolutionary models. Although 35% is a high proportion of missing values, few fossils included have high percentage of missing values in the final dataset. The average percentages of missing values in the fossils for the neurocranial dataset is 3.82% and for the face it is 8.42% (See individual information in Table S1).

All individuals were transposed to the same origin, rotated to common axes of orientation, and scaled to the same size using Generalized Procrustes Analyses (GPA), and the transformed coordinates were used to calculate the Principal Components used in the tests of evolutionary models. Missing data estimation and GPA were performed in R^35^, using the geomorph package^36,37^.

### Principal component analyses

The evolutionary models tested in this study are based on theoretical assumptions about changes of variance and means of linear traits over evolutionary lineages^20^, which require the 3D morphometric data to be converted into linear dimensions. In this study, we rotated the landmark coordinates through Principal Components Analysis and used the scores of the first four Principal Components as variables to be tested against the evolutionary models, as detailed below. The choice of using Principal Components Scores as measurements of morphological trends allows the morphometric information available in 3D data to be explored across the axes of largest variance in the dataset, without the need to explore combinations of linear measurements between landmarks. As the Principal Components with the largest eigenvalues represent the axes of largest shared variance of landmarks across the specimens in the dataset, they represent the most important dimensions of morphological change among *Homo*, and each of the OTUs can be visualized along those axes. Moreover, as the axes of largest shared variance in the data, they also correlate strongly with the dimensions of theoretical highest evolvability in *Homo*^38,39^, i.e., the dimensions along which more variance is available to be pulled by selective pressures in the environment. As such, the first Principal Components scores represent dimensions of morphological change with the highest potential for responding to selective pressures, and are in theory the most likely to show strong responses to selective evolutionary pressures.

The evolutionary model fitting was limited to four Principal Components because the lower principal components explain smaller amounts of information, and are more influenced by variance within OTUs than variance that derives from differences between OTUs, which limits their explanatory power of morphological trends over time. In all datasets, the cumulative variance explained by the first four PCs is at least 50% of the variance in the original data. Details about the PCs’ eigenvalues and variance explained are reported in Table S4. The PC scores for specimens in each OTU were visualized through violin plots, and the morphological changes that are represented in each PC were illustrated by deforming wireframes connecting some of the landmarks to the minimum and maximum values observed in each PC. Principal components calculations and visualization were performed in R 4.5.2^35^, supported by packages MASS^40^, ggplot2^41^, and plotly^42^.

### Analysis of size

The morphological analysis of shape variation represented through the Principal Component scores of the Procrustes-transformed data was complemented with the analysis of variation in size across the *Homo* lineage. Size changes were analyzed by extracting the centroid size of each specimen, calculated from the GPA analysis, and were analyzed via the model-testing procedure in the same way as the PC scores.

### Evolutionary model testing

As defined by Hunt^20,43,44^, different evolutionary models can be conceptualized based on the magnitude and direction of trait averages’ change over time, in function of the variance observed in each of the lineage’s steps. As the expected outcomes of different evolutionary processes can be derived based on basic descriptive parameters of seriated samples (mean, variance, and relative time separating populations), they can be contrasted to observed data and their fit to the data can be quantified. Different evolutionary processes generate different expectations, and their relative goodness-of-fit to an evolutionary time series can be contrasted to other models using maximum likelihood estimates, which return the relative likelihood of different models tested to explain the variation and change observed in the measured data^20^.

Hunt^43,44^ derived the expectations of several distinct evolutionary models that can be fit against linear variables from seriated data. Here, we implement Hunt’s method to explore the evolutionary models that have the strongest fit to the craniofacial morphological variation of the genus *Homo*. We selected to test the fit of six different evolutionary models to the dimensions of morphological change defined by the Principal Components scores calculated from 3D morphometric data, as detailed before. The models tested are:

1. General Random Walk (GRW): this model assumes that changes over time are the result of a biased walk, where changes in each step are drawn from a distribution with non-zero mean, and reflect the process that would be expected for lineages under a relatively constant and gradual directional selective pull from the environment. Under this model, evolutionary changes for each step in the lineage are drawn from a distribution with a non-zero mean step, which determines the direction of trait change, and variance that determines volatility of each step, which defines how much steps can vary from each other.
2. Unbiased Random walk (URW): this model assumes that evolution is non-directional, and the accumulation of change over time is the product of the random sampling of the variance distribution at each step of the lineage. In this model, evolutionary changes at each step are drawn from a distribution with mean 0, and variance representing the volatility of each step. This model assumes that changes accumulate over time, but in a meandering way, representing stochastic or neutral evolutionary processes.
3. Evolutionary Stasis (ES): this model assumes that there exists an optimum phenotype around which some variation is permitted, but due to the constraints acting to define the optimum, no accumulation of net morphological differences is seen over time. This model assumes that the traits for all steps are normally distributed around the optimal phenotype with a variance value that defines the magnitude of fluctuations that can be observed around the fixed mean.
4. Strict Stasis (StS): This is a stricter version of the previous model, where all variation observed is assumed to be random noise and there is a strong pull around one same optimum phenotype across the entire lineage. The difference between this and the previous model is that in this stricter version of stasis, the expected variance around the optimal mean is zero.
5. Ornstein-Uhlenbeck (OU): this model assumes that a population is orbiting around a nearby peak in the adaptive landscape, causing the trait to oscillate around the peak. The evolution towards the adaptive peak depends on the optimal trait value, and the strength of attraction to that optimal, variation of the distribution of which each step is taken (a measurement of genetic drift) and the value of the trait at the start of the evolutionary sequence.
6. Punctuated Equilibrium (PE): this model assumes that there is a period of quick shift in the value of a trait, which separates periods of relative stability. Each period of stability in this case is defined by its own optimal value and variance around it. While the model accommodates the test of multiple possible steps of change, our analyses were limited to the search of only one step, due to the reduced number of OTUs available in our sequence. In this case, the model tests all possible steps in the evolutionary sequence where a shift could have occurred, reporting back the step that results in the strongest fit to the data.

The fit of different models can be compared to each other using maximum likelihood estimates and Akaike weights (standardized from the Akaike Information Criteria) are used to evaluate the relative plausibility of each model. The Akaike Information Criteria penalizes complex models over simpler ones, to avoid overfitting of models to the data and maximize the generalization that can be derived from the model. This is relevant to this study because in situations where the log likelihood between the different models is similar (i.e., that is, when there are no significant differences in how well models fit the data based on the maximum likelihood estimate), the Akaike weight will favor the simplest model. Model complexity is defined by the number of parameters included in each of the six models tested here. OU and PE are the more complex models (4 parameters), followed by GRW (3 parameters), then URW and ES (2 parameters) and finally StS (1 parameter). We use the Akaike weights in this study to contrast the fit of the different models to the Principal Components scores and to the centroid size of each dataset tested.

The evolutionary models tested assume that all steps in the temporal sequence tested are part of one single lineage, i.e., there is a direct ancestor-descendant relationship between all steps in the series. However, this is not a reasonable assumption in the genus *Homo*, especially when looking at the later taxa, given that *Homo neanderthalensis* and *Homo sapiens* are sister taxa, and the latter is not directly descended from the former. For this reason, we tested two different evolutionary sequences for *Homo*. The first one removes *Homo neanderthalensis* from the analysis, creating a time series that connects early *Homo* to archaic humans, to *Homo sapiens* (Table S1). The *H. sapiens* lineage is the most parsimonious sequence to explain the origin of modern humans through direct descent of the *Homo* fossils known to date. The second sequence removes all *H. sapiens* OTUs and, similar to the previous one, establishes a fossil sequence that leads directly to the appearance of *Homo neanderthalensis*. This *H. neanderthalensis* lineage explores the evolution of the *Homo* branch that led to *H. neanderthalensis*. However, it is a time series with a more limited number of OTUs and smaller sample sizes than the other lineage (Table S1). As described above, we explored five different alternative scenarios for these two lineages, changing the OTUs and fossils in them, to evaluate the biases introduced by our OTU definition, given the limited number of fossil specimens available to study.

For each lineage, we contrasted the fit of the six evolutionary models on two different datasets, to test our hypotheses. The first dataset considers only landmarks of the face and the second dataset only landmarks of the neurocranium. All model testing analyses were performed in R 4.5.2, using package PaleoTS^45^.

### Analysis replicability

All analyses were conducted in R 4.5.2, using packages described in the previous sections. To permit the replication of the study, a commented document with all code, links to external resources, and results was compiled and is shared in html and quarto formats (Supplementary Code 1).

### Reporting summary

Further information on research design is available in the Nature Portfolio Reporting Summary linked to this article.

## Supplementary information

Supplementary Information

Description of Additional Supplementary Files

Supplementary Code 1

Reporting Summary

Transparent Peer Review file

## Data Availability

The neurocranial and facial datasets used in this study have been deposited in Zenodo and are available at 10.5281/zenodo.20049310^46^.

## Code availability

HTML and Quarto documents with all code, links to external resources, and results are provided in Supplementary Code 1.
