# Supplementary material for: Evolutionary drivers of encephalization and facial reduction in the genus Homo
Source: Nat Commun. 2026 Jul 6;17:5625. doi: 10.1038/s41467-026-74739-w (PMC13338430; doi:10.1038/s41467-026-74739-w)
Supplement: Supplementary file 1 — Supplementary Information [file 41467_2026_74739_MOESM1_ESM.pdf]

Table S1 - Hominin specimens included in the study and their contextual information

ID	Specimen	OTU	Included in Homo sapiens lineage	Included in Homo neanderthalensis lineage	Included in Face analyses	Percentage of Missing Landmarks in Face	Included in Neurocraneum analyses	Percentage of Missing Landmarks in Neurocraneum	Chronology	Reference
HHBRDU002HA	KNM ER 1470	Early *Homo*	Yes	Yes	Yes	17.39	Yes	19.05	1.9MA	LEAKEY, R. Nature 242, 447–450 (1973). https://doi.org/10.1038/242447a0; Hillhouse, J., Ndombi, J., Cox, A. et al. Nature 265, 411–415 (1977). https://doi.org/10.1038/265411a0; Gleadow, Nature 284, 225–230 (1980). https://doi.org/10.1038/284225a0
HHBHBU001HA	KNM ER 1813	Early *Homo*	Yes	Yes	Yes	8.7	Yes	0	1.9MA	Leakey, R. Nature 248, 653–656 (1974). https://doi.org/10.1038/248653a0
HMEDMU003HA	Dmanisi 2280	*H. erectus*	Yes	Yes	No		Yes	0	1.8MA	Lordkipanidze et al. Science 342, 326-331(2013).DOI:10.1126/science.1238484
HMEDMU008HA	Dmanisi 2282	*H. erectus*	Yes	Yes	Yes	21.74	Yes	9.52	1.8MA	Lordkipanidze et al. Science 342, 326-331(2013).DOI:10.1126/science.1238484
HMEERU004HA	KNM ER 3733	*H. erectus*	Yes	Yes	Yes	34.78	Yes	0	1.8MA	Lepre, C.J., Kent, DV. Earth and Planetary Science Letters 290, 362-374 (2010). doi.org/10.1016/j.epsl.2009.12.032.
HMEERU005HA	KNM ER 3883	*H. erectus*	Yes	Yes	No		Yes	0	1.6MA	Leakey, R.E.F. and Walker, A.C. Am. J. Phys. Anthropol., 67: 135-163 (1985). https://doi-org.proxy.lib.ohio-state.edu/10.1002/ajpa.1330670209
HMEERU002HS	KNM WT 15000	*H. erectus*	Yes	Yes	Yes	4.35	Yes	4.76	1.6MA	Walker, A., Leakey, R., (Eds.) The Nariokotome Homo erectus Skeleton, Harvard University Press, Cambridge (1993), pp. 63-94
HMEOHM007HA	OH9	*H. erectus*	Yes	Yes	No		Yes	4.76	1.4MA	Antón, S.C. Am. J. Phys. Anthropol., 122: 126-170 (2003). https://doi-org.proxy.lib.ohio-state.edu/10.1002/ajpa.10399
HMHARU011HA	Arago 21[a]	*H. heidelbergensis* s.l.	Yes	Yes	Yes	0	No		600-350	de Lumley and de Lumley, 1973 - Yearb. Phys. Anthropol., 17 (1973), pp. 162-168; Cook et al., 1982 - Yearbk. Phys. Anthropol., 25 (1982), pp. 19-65; Falguères et al., 2004 - J. Archaeol. Sci., 31 (2004), pp. 941-952
HMHBDM006HA	Bodo	*H. heidelbergensis* s.l.	Yes	Yes	Yes	4.35	No		600	Clark, J. D., et al., Science 264, 1907–1910 (1994). http://www.jstor.org/stable/2883979
HMHKBM001HA	Broken Hill (Kabwe)	*H. heidelbergensis* s.l.	Yes	Yes	Yes	0	Yes	0	299+-25	Grün, R., Pike, A., McDermott, F. et al.. Nature 580, 372–375 (2020). https://doi.org/10.1038/s41586-020-2165-4
HMHDAU017HA	Dali	*H. heidelbergensis* s.l.	Yes	Yes	Yes	21.74	Yes	0	260+-20	Sun, X., et al. Quaternary International 434, 99-106 (2017). https://doi.org/10.1016/j.quaint.2015.05.027.
HMHNDF007HA	Ndutu	*H. heidelbergensis* s.l.	Yes	Yes	No		Yes	4.76	600-400	Mturi, A., Nature 262, 484–485 (1976). https://doi.org/10.1038/262484a0; Rightmire, G.P. Am. J. Phys. Anthropol., 61: 245-254 (1983). https://doi-org.proxy.lib.ohio-state.edu/10.1002/ajpa.1330610214
HMHPEM002HA	Petralona	*H. heidelbergensis* s.l.	Yes	Yes	Yes	0	Yes	19.05	620-350	Papamarinopoulos, S., et al., Archaeometry, 29: 50-59 (2007). https://doi-org.proxy.lib.ohio-state.edu/10.1111/j.1475-4754.1987.tb00397.x
HMHSMM005HA	Sima Craneo 5	*H. heidelbergensis* s.l.	Yes	Yes	Yes	8.7	Yes	0	400-450	Demuro, M., et al., J Hum Evol 131, 76-95 (2019) https://doi.org/10.1016/j.jhevol.2018.12.003.; Arsuaga J.L., et al., Science. 344:1358-63 (2014). doi: 10.1126/science.1253958.
Apidima 2	Apidima 2	early *H. neanderthalensis*	No	Yes	Yes	17.39	No		170	Harvati, K., Röding, C., Bosman, A.M. et al. Nature 571, 500–504 (2019). https://doi.org/10.1038/s41586-019-1376-z
HMNEAF006HA	Krapina 3	early *H. neanderthalensis*	No	Yes	Yes	26.09	Yes	28.57	140-120	Rink, W., Schwarcz, H., Smith, F. et al. Nature 378, 24 (1995). https://doi.org/10.1038/378024a0

HMNEAF009HA	Saccopastore 1	early *H. neanderthalensis*	No	Yes	Yes	30.43	Yes	14.29	295-220	Marra, F., et al, PLoS One e0170434 (2017). https://doi.org/10.1371/journal.pone.0170434
HMNNEF013HA	Tabun C1	early *H. neanderthalensis*	No	Yes	Yes	30.43	Yes	0	130-100 ka	Grün, R., Stringe, C., J Hum. Evol. 39, 601-612 (2000). https://doi.org/10.1006/jhev.2000.0443;
HMNNEM001HA	Amud 1	*H. neanderthalensis*	No	Yes	Yes	17.39	Yes	0	55-60	Valladas, H., et al., J. Arch. Sci. 26 259-268 (1999). https://doi.org/10.1006/jasc.1998.0334.
HMNWEF004HA	Gibraltar 1	*H. neanderthalensis*	No	Yes	Yes	8.7	Yes	0	50	Oakley, K.P., et al. Catalogue of Fossil Hominids Part II: Europe. Br. Mus. Nat. Hist., London.
HMNWEM005HA	Guattari 1	*H. neanderthalensis*	No	Yes	Yes	8.7	Yes	0	50-60	Grün, R., Stringer, C. B., Archaeometry 33, 153:199 (1991) https://doi.org/10.1111/j.1475-4754.1991.tb00696.x ;Schwarcz H. P., et al., Cur Anthropolol 32, 313-316 (1991). https://doi.org/10.1086/203959
HMNWEM002HA	La Chapelle aux Saints	*H. neanderthalensis*	No	Yes	Yes	17.39	Yes	0	56-47	Grün, R., Stringer, C. B., Archaeometry 33, 153:199 (1991) https://doi.org/10.1111/j.1475-4754.1991.tb00696.x
HMNWEM003HA	La Ferrassie 1	*H. neanderthalensis*	No	Yes	Yes	0	Yes	0	43-45	Guérin, G., et al., J Arch Sci 58, 147-166 (2015). https://doi.org/10.1016/j.jas.2015.01.019.
HMNWEF008HA	Quina 5	*H. neanderthalensis*	No	Yes	No		Yes	0	>48 (MIS 3-4)	Debénath, A., Jelinek, A. Gallia Prehist. 40, 29-74 (1998)
HMNNEM010HA	Shanidar 1	*H. neanderthalensis*	No	Yes	Yes	0	Yes	0	46-50 Uncalibrated	Vogel, J.C. , Waterbolk, H.T., Radiocarbon 5 163-2502 (1963)
HMNWEU018HA	Shanidar 5	*H. neanderthalensis*	No	Yes	Yes	4.35	No		46-50 Uncalibrated	Vogel, J.C. , Waterbolk, H.T., Radiocarbon 5 163-2502 (1963)
HMSAMU011HA	Das Es Soltane 5	early *H. sapiens*	Yes	No	Yes	21.74	Yes	28.57		Hublin, J.-J. Philosophical Transactions: Biological Sciences 337, 185–191 (1992). http://www.jstor.org/stable/57106
HMHIRU004HA	Jebel Irhoud 1	early *H. sapiens*	Yes	No	Yes	8.7	Yes	0	315+-34	Richter D., et al., Nature 546, 293-296 (2017). doi: 10.1038/nature22335.
HMHIRU010HA	Jebel Irhoud 2	early *H. sapiens*	Yes	No	No		Yes	0	315+-34	Richter D., et al., Nature 546, 293-296 (2017). doi: 10.1038/nature22335.
HMHLHU008HA	LH18	early *H. sapiens*	Yes	No	No		Yes	0	120	Magori, C.C., Day, M.H. J Hum Evol 12, 747-753. https://doi.org/10.1016/S0047-2484(83)80130-4.
HMSOMU001HA	Omo 1	early *H. sapiens*	Yes	No	No		Yes	4.76	195+-5	McDougall, I., Brown, F. & Fleagle, J. . Nature 433, 733–736 (2005). https://doi.org/10.1038/nature03258
HMSAMM001HA	Qafzeh 6	early *H. sapiens*	Yes	No	Yes	0	Yes	0	135-100	Grün, R., et al. J Hum Evol 49, 316-334 (2005). https://doi.org/10.1016/j.jhevol.2005.04.006.
HMSAMF002HA	Qafzeh 9	early *H. sapiens*	Yes	No	Yes	0	Yes	0	135-100	Grün, R., et al. J Hum Evol 49, 316-334 (2005). https://doi.org/10.1016/j.jhevol.2005.04.006.
HMSAMM004HA	Skhul 5	early *H. sapiens*	Yes	No	Yes	30.43	Yes	0	135-100	Grün, R., et al. J Hum Evol 49, 316-334 (2005). https://doi.org/10.1016/j.jhevol.2005.04.006.
HMSUPF010HA	Abri Pataud	Upper Paleolithic *H. sapiens*	Yes	No	Yes	0	Yes	0	28-26 (22 uncalibrated)	Douka, K., et al. J Hum Evol 141, 102730 (2020). https://doi.org/10.1016/j.jhevol.2019.102730.
HMSUPU036HA	Brno 2	Upper Paleolithic *H. sapiens*	Yes	No	No		Yes	9.52	23.7 uncalibrated (ca. 28.5 cal BP)	Pettitt, P., Trinkaus, E., Anthropologie, 38: 149-150 (2000)
HMSUPU011HA	Chancelade	Upper Paleolithic *H. sapiens*	Yes	No	Yes	8.7	Yes	0	18	Barshay-Szmidt, C., et al., J Arch Sci: Reports 17: 809-838 (2016). https://doi.org/10.1016/j.jasrep.2017.09.003.
HMSUPU015HA	Cioclovina	Upper Paleolithic *H. sapiens*	Yes	No	No		Yes	0	ca33	Harvati, K., et al. J Hum Evol 2007, 732-746 (2007). https://doi.org/10.1016/j.jhevol.2007.09.009.
HMSUPM001HA	Cro Magnon 1	Upper Paleolithic *H. sapiens*	Yes	No	Yes	13.04	Yes	0	ca 30	Movius, H.L., Anuario Estudios Atlanticos 15, 323-344 (1969); Henry-Gambier, D., et al., Paleo 24, 121-138 (2002). https://doi.org/10.4000/paleo.2859

HMSUPF002HA	Cro-Magnon 2	Upper Paleolithic *H. sapiens*	Yes	No	Yes	8.7	Yes	9.52	ca 30	Movius, H.L., Anuario Estudios Atlanticos 15, 323-344 (1969); Henry-Gambier, D., et al., Paleo 24, 121-138 (2002). https://doi.org/10.4000/paleo.2859
HMSUPM030HA	Dolni Vestonice 13	Upper Paleolithic *H. sapiens*	Yes	No	Yes	0	Yes	0	ca. 31	Fewlass, H., et al., J Arch Sci 27: 102000 (2019). https://doi.org/10.1016/j.jasrep.2019.102000.
HMSUPM032HA	Dolni Vestonice 15	Upper Paleolithic *H. sapiens*	Yes	No	Yes	4.35	Yes	0	ca. 31	Fewlass, H., et al., J Arch Sci 27: 102000 (2019). https://doi.org/10.1016/j.jasrep.2019.102000.
HMSUPM033HA	Dolni Vestonice 16	Upper Paleolithic *H. sapiens*	Yes	No	Yes	0	Yes	0	ca. 30	Fewlass, H., et al., J Arch Sci 27: 102000 (2019). https://doi.org/10.1016/j.jasrep.2019.102000.
HMSUPF028HA	Dolni Vestonice 3	Upper Paleolithic *H. sapiens*	Yes	No	Yes	0	Yes	0	undated	Fewlass, H., et al., J Arch Sci 27: 102000 (2019). https://doi.org/10.1016/j.jasrep.2019.102000.
HMSUPU025HA	Grimaldi	Upper Paleolithic *H. sapiens*	Yes	No	Yes	0	Yes	0	25 uncal (ca. 29.5 cal BP)	Formicola, V., et al., Curr. Anthropol., 45, 114-118 (2004). https://doi.org/10.1086/381008
HMSHOU017HA	Hofmeyr	Upper Paleolithic *H. sapiens*	Yes	No	Yes	8.7	Yes	14.29	36	Grine, F.E., et al., Science 315,226-229 (2007). DOI:10.1126/science.1136294
HMSIEU001HA	Iwo Eleru	Upper Paleolithic *H. sapiens*	Yes	No	No		Yes	0	13	Harvati K., et al., PLoS One 6, e24024 (2011). doi: 10.1371/journal.pone.0024024.
HMSKLM043HA	Kanalda	Upper Paleolithic *H. sapiens*	Yes	No	Yes	0	Yes	9.52	10	Storm, P. (1995). The evolutionary significance of the Wajak skulls. Scripta Geologica, 110, 1–248.
HMSUPU041HS	Lau bassa	Upper Paleolithic *H. sapiens*	Yes	No	No		Yes	9.52	10	
HMSUPF003HA	Mladec 1	Upper Paleolithic *H. sapiens*	Yes	No	Yes	0	Yes	9.52	35-36.5	Wild, E., et al. . Nature 435, 332–335 (2005). https://doi.org/10.1038/nature03585
HMSUPF004HA	Mladec 2	Upper Paleolithic *H. sapiens*	Yes	No	Yes	13.04	Yes	4.76		Wild, E., et al. . Nature 435, 332–335 (2005). https://doi.org/10.1038/nature03585
HMSUPM008HA	Mladec 5	Upper Paleolithic *H. sapiens*	Yes	No	No		Yes	0	31	Wild, E., et al. . Nature 435, 332–335 (2005). https://doi.org/10.1038/nature03585
HMSUPM009HA	Mladec 6	Upper Paleolithic *H. sapiens*	Yes	No	No		Yes	19.05	31	Wild, E., et al. . Nature 435, 332–335 (2005). https://doi.org/10.1038/nature03585
HMSUPF039HA	Muierii 1	Upper Paleolithic *H. sapiens*	Yes	No	Yes	0	No		ca. 35	Soficaru, A., Dobos, A, Trinkaus E., Proc. Natl. Acad. Sci. U.S.A. 103, 17196-17201 (2006), https://doi.org/10.1073/pnas.0608443103
HMSUPF037HA	Oase 2	Upper Paleolithic *H. sapiens*	Yes	No	Yes	0	Yes	0	ca. 40,5	Rougier, H., Proc. Natl. Acad. Sci. U.S.A. 104, 1165-1170 (2007). https://doi.org/10.1073/pnas.0610538104
HMSUPU026HA	Ohalo II	Upper Paleolithic *H. sapiens*	Yes	No	Yes	0	Yes	0	19	Hershkovitz I., et al., Am J Phys Anthropol. 96, 215-34 (1995). doi: 10.1002/ajpa.1330960302.
HMSUPM034HA	Pavlov 1	Upper Paleolithic *H. sapiens*	Yes	No	No		Yes	0	25-26	Fewlass, H., et al., J Arch Sci 27: 102000 (2019). https://doi.org/10.1016/j.jasrep.2019.102000.
HMSUPM005HA	Predmost 3	Upper Paleolithic *H. sapiens*	Yes	No	No		Yes	0	27-29	Svoboda, J. A., J Hum Evol 54, 15-33 (2008). https://doi.org/10.1016/j.jhevol.2007.05.016.
HMSUPF006HA	Predmost 4	Upper Paleolithic *H. sapiens*	Yes	No	Yes	0	Yes	0	27-29	Svoboda, J. A., J Hum Evol 54, 15-33 (2008). https://doi.org/10.1016/j.jhevol.2007.05.016.
HMSUPM014HA	Upper Cave 101	Upper Paleolithic *H. sapiens*	Yes	No	Yes	0	Yes	0	24-29	Harvati, K., Röding, C., Bosman, A.M. et al. Nature 571, 500–504 (2019). https://doi.org/10.1038/s41586-019-1376-z
HMSUPF016HA	Upper Cave 103	Upper Paleolithic *H. sapiens*	Yes	No	Yes	4.35	Yes	9.52	24-29	Harvati, K., Röding, C., Bosman, A.M. et al. Nature 571, 500–504 (2019). https://doi.org/10.1038/s41586-019-1376-z
HMSUPU027HA	Wadi Kubbaniya	Upper Paleolithic *H. sapiens*	Yes	No	Yes	0	No		20	Wendorf F, Schild R. The Wadi Kubbaniya Skeleton: A Late Paleolithic Burial from Southern Egypt (1986).
HMSAUF029HA	Australian19	*H. sapiens*	Yes	No	Yes	0	Yes	0	<1	
HMSAUM003HA	Australian21	*H. sapiens*	Yes	No	Yes	0	Yes	0	<1	
HMSCHF001HA	Chinese01	*H. sapiens*	Yes	No	Yes	0	Yes	0	<1	

HMSCHM002HA	Chinese12	*H. sapiens*	Yes	No	Yes	0	Yes	0	<1
HMSEFF004HA	East African07	*H. sapiens*	Yes	No	Yes	0	Yes	0	<1
HMSEFM005HA	East African09	*H. sapiens*	Yes	No	Yes	0	Yes	0	<1
HMSESF042HA	Eskimo (Inusguk)08	*H. sapiens*	Yes	No	Yes	0	Yes	0	<1
HMSESM046HA	Eskimo (Inusguk)13	*H. sapiens*	Yes	No	Yes	0	Yes	0	<1
HMSNEM010HA	Europe02	*H. sapiens*	Yes	No	Yes	0	Yes	0	<1
HMSNEF008HA	Europe14	*H. sapiens*	Yes	No	Yes	0	Yes	0	<1
HMSGGF003HA	Grand Gulch1	*H. sapiens*	Yes	No	Yes	0	Yes	0	<1
HMSGGM007HA	Grand Gulch12	*H. sapiens*	Yes	No	Yes	0	Yes	0	<1
HMSSMF015HA	KhoiSan12	*H. sapiens*	Yes	No	Yes	0	Yes	0	<1
HMSSMM048HA	KhoiSan28	*H. sapiens*	Yes	No	Yes	0	Yes	0	<1
HMSANF017HA	Melanesian07	*H. sapiens*	Yes	No	Yes	0	Yes	0	<1
HMSANM025HA	Melanesian14	*H. sapiens*	Yes	No	Yes	0	Yes	0	<1
HMSSRM016HA	Near Eastern08	*H. sapiens*	Yes	No	Yes	0	Yes	0	<1
HMSSRF004HA	Near Eastern15	*H. sapiens*	Yes	No	Yes	0	Yes	0	<1
HMSTSF003HA	Tasmania1	*H. sapiens*	Yes	No	Yes	0	Yes	0	<1
HMSTSM002HA	Tasmania5	*H. sapiens*	Yes	No	Yes	0	Yes	0	<1
HMSTHF016HA	Thai06	*H. sapiens*	Yes	No	Yes	0	Yes	0	<1
HMSTHM018HA	Thai08	*H. sapiens*	Yes	No	Yes	0	Yes	0	<1
HMSZUM001HA	Zulu01	*H. sapiens*	Yes	No	Yes	0	Yes	0	<1
HMSZUF009HA	Zulu23	*H. sapiens*	Yes	No	Yes	0	Yes	0	<1

[a] – landmarks for this fossil were collected on the reconstruction from *Gunz, P., Mitteroecker, P., Neubauer, S., Weber, G. W. & Bookstein, F. L. J. Hum. Evol. 57, 48–62 (2009)*.

Table S2 - Neurocranial landmarks included in the analysis and percentage of missing values in each.

Landmarks in Neurocraneum Dataset	Percentage of Missing Landmarks
Inion	7.02
Asterion R	1.75
Asterion L	1.75
Lambda	5.26
Stylomastoid For. R	10.53
Porion R	0
Auriculare R	0
Parietal Notch R	3.51
Mastoidiale R	5.26
Lat. Glenoid R	8.77
Stylomastoid For. L	10.53
Porion L	0
Auriculare L	0
Parietal Notch L	3.51
Mastoidiale L	5.26
Lat. Glenoid L	8.77
Bregma	1.75
Post-toral sulcus	1.75
Glabella	7.02
Stephanion R	1.75
Stephanion L	1.75

Table S3 - Facial landmarks included in the analysis and percentage of missing values in each.

**Landmarks in Face Dataset**	**Percentage of Missing Landmarks**
Canine-Premolar contact R	21.28
Canine-Premolar contact L	25.53
Distal M3 R	25.53
Distal M3 L	25.53
Nasion	6.38
Nasospinale	10.64
Prosthion	6.38
Mid-orbit torus superior R	2.13
Mid-orbit torus inferior R	2.13
Dacryon R	2.13
Zygoorbitale R	6.25
Frontomalare Orb R	2.13
Infraorbital for.R	6.38
Zygomaxillare R	8.51
Alare R	4.26
Mid-orbit torus superior L	2.13
Mid-orbit torus inferior L	2.13
Dacryon L	2.13
Zygoorbitale L	8.33
Frontomalare Orb L	2.13
Infraorbital for. L	6.38
Zygomaxillare L	8.51
Alare L	4.26

Table S4 - Eigenvalues and variance explained by the first four principal components for each of the four datasets analyzed

PC	eigenvalues	variance	cumulative eigenvalue	cumulative variance
**Neurocranial Analysis - H. sapiens lineage**				
1	0.0021177	27.585	0.0021177	27.585
2	0.0010838	14.118	0.0032015	41.703
3	0.0005632	7.336	0.0037647	49.039
4	0.0004855	6.325	0.0042502	55.364
**Neurocranial Analysis - H. neanderthalensis lineage**				
1	0.0028512	33.86	0.0028512	33.86
2	0.0011894	14.125	0.0040406	47.985
3	0.0007426	8.819	0.0047832	56.804
4	0.000705	8.372	0.0054882	65.176
**Facial Analysis - H. sapiens lineage**				
1	0.0023441	22.122	0.0023441	22.122
2	0.0012312	11.619	0.0035753	33.741
3	0.0011311	10.674	0.0047063	44.415
4	0.000703	6.634	0.0054093	51.049
**Facial Analysis - H. neanderthalensis lineage**				
1	0.0023441	22.122	0.0023441	22.122
2	0.0012312	11.619	0.0035753	33.741
3	0.0011311	10.674	0.0047063	44.415
4	0.000703	6.634	0.0054093	51.049

Table S5 - Model comparison results for the four Principal Components and centroid sizes tested in the four datasets analyzed.

Neurocranial Analysis - Homo sapiens lineage

Model	logL	K	AICc	dAICc	Akaike weight
**Principal Component 1**					
General Random Walk	-1.63673	3	21.27347	6.5694	**0.036**
Unbiased Random Walk	-3.35203	2	14.70407	0	**0.961**
Stasis	-9.07623	2	26.15245	11.44838	**0.003**
Strict Stasis	-265.323	1	533.6465	518.9424	**0**
Punctuated Equilibrium	-3.29176	4	54.58352	39.87945	**0**
Ornstein-Uhlenbeck	-1.64051	4	51.28102	36.57695	**0**
**Principal Component 2**					
General Random Walk	-6.05763	3	30.11525	9.779557	**0.003**
Unbiased Random Walk	-6.16785	2	20.3357	0	**0.416**
Stasis	-6.68028	2	21.36056	1.024864	**0.249**
Strict Stasis	-8.89694	1	20.79389	0.458189	**0.331**
Punctuated Equilibrium	-5.13993	4	58.27985	37.94415	**0**
Ornstein-Uhlenbeck	-3.89726	4	55.79453	35.45883	**0**
**Principal Component 3**					
General Random Walk	-5.60964	3	29.21929	15.30798	**0**
Unbiased Random Walk	-5.66264	2	19.32528	5.413973	**0.059**
Stasis	-2.95566	2	13.91131	0	**0.889**
Strict Stasis	-8.30902	1	19.61805	5.706736	**0.051**
Punctuated Equilibrium	-1.85683	4	51.71367	37.80235	**0**
Ornstein-Uhlenbeck	-2.55725	4	53.11449	39.20318	**0**

Neurocranial Analysis - Homo neanderthalensis lineage

Model	logL	K	AICc	dAICc	Akaike weight
**Principal Component 1**					
General Random Walk	2.278425	3	25.44315	7.339272	**0.024**
Unbiased Random Walk	-4.05194	2	18.10388	0	**0.931**
Stasis	-7.08233	2	24.16466	6.060783	**0.045**
Strict Stasis	-46.7621	1	96.85759	78.75372	**0**
Punctuated Equilibrium	-1.23495	4	Inf	Inf	**0**
Ornstein-Uhlenbeck	2.366775	4	Inf	Inf	**0**
**Principal Component 2**					
General Random Walk	-6.01475	3	42.02949	26.89221	**0**
Unbiased Random Walk	-6.03013	2	22.06027	6.922987	**0.026**
Stasis	-4.27204	2	18.54407	3.406793	**0.15**
Strict Stasis	-5.90197	1	15.13728	0	**0.824**
Punctuated Equilibrium	-3.79446	4	Inf	Inf	**0**
Ornstein-Uhlenbeck	-3.99458	4	Inf	Inf	**0**
**Principal Component 3**					
General Random Walk	-2.60428	3	35.20856	26.8309	**0**
Unbiased Random Walk	-2.6101	2	15.22019	6.842529	**0.03**
Stasis	-2.02818	2	14.05636	5.678697	**0.054**
Strict Stasis	-2.52216	1	8.377661	0	**0.916**
Punctuated Equilibrium	-1.94158	4	Inf	Inf	**0**
Ornstein-Uhlenbeck	-1.76813	4	Inf	Inf	**0**

Facial Analysis - Homo sapiens lineage

Model	logL	K	AICc	dAICc	Akaike weight
**Principal Component 1**					
General Random Walk	-4.43241	3	26.86482	5.248694	**0.041**
Unbiased Random Walk	-7.19796	2	22.39592	0.7798	**0.387**
Stasis	-6.80806	2	21.61612	0	**0.571**
Strict Stasis	-16.3714	1	35.74281	14.12669	**0**
Punctuated Equilibrium	-3.11084	4	54.22167	32.60555	**0**
Ornstein-Uhlenbeck	-5.62501	4	59.25001	37.63389	**0**
**Principal Component 2**					
General Random Walk	-4.51059	3	27.02118	13.17621	**0.001**
Unbiased Random Walk	-5.45858	2	18.91717	5.072188	**0.062**
Stasis	-4.59018	2	17.18037	3.335387	**0.149**
Strict Stasis	-5.42249	1	13.84498	0	**0.788**
Punctuated Equilibrium	-4.11922	4	56.23844	42.39346	**0**
Ornstein-Uhlenbeck	-2.69619	4	53.39237	39.5474	**0**
**Principal Component 3**					
General Random Walk	-7.30407	3	32.60815	9.517902	**0.005**
Unbiased Random Walk	-7.8033	2	23.6066	0.516353	**0.434**
Stasis	-7.54512	2	23.09024	0	**0.561**
Strict Stasis	-66.169	1	135.3381	112.2478	**0**
Punctuated Equilibrium	-1.4987	4	50.99739	27.90715	**0**
Ornstein-Uhlenbeck	-6.81041	4	61.62083	38.53058	**0**

Facial Analysis - Homo neanderthalensis lineage

Model	logL	K	AICc	dAICc	Akaike weight
**Principal Component 1**					
General Random Walk	-2.39373	3	34.78746	26.2833	**0**
Unbiased Random Walk	-2.61518	2	15.23037	6.726206	**0.032**
Stasis	-2.58542	2	15.17085	6.666685	**0.033**
Strict Stasis	-2.58541	1	8.50416	0	**0.934**
Punctuated Equilibrium	-2.40174	4	Inf	Inf	**0**
Ornstein-Uhlenbeck	-2.36427	4	Inf	Inf	**0**
**Principal Component 2**					
General Random Walk	1.177631	3	27.64474	10.08866	**0.006**
Unbiased Random Walk	-3.77804	2	17.55607	0	**0.951**
Stasis	-6.88401	2	23.76802	6.211948	**0.043**
Strict Stasis	-23.636	1	50.60539	33.04931	**0**
Punctuated Equilibrium	0.016659	4	Inf	Inf	**0**
Ornstein-Uhlenbeck	1.243202	4	Inf	Inf	**0**
**Principal Component 3**					
General Random Walk	-4.39305	3	38.78609	26.58426	**0**
Unbiased Random Walk	-4.45427	2	18.90853	6.706697	**0.032**
Stasis	-3.94709	2	17.89419	5.692352	**0.053**
Strict Stasis	-4.43425	1	12.20184	0	**0.915**
Punctuated Equilibrium	-3.03882	4	Inf	Inf	**0**
Ornstein-Uhlenbeck	-3.83964	4	Inf	Inf	**0**

	Principal Component 4					Principal Component 4					Principal Component 4					Principal Component 4				
General Random Walk	-2.33301	3	22.66602	7.546935	**0.01**	-5.74788	3	41.49576	25.40533	**0**	-6.96357	3	31.92713	9.925849	**0.003**	-3.46495	3	36.9299	16.32631	**0**
Unbiased Random Walk	-3.86126	2	15.72251	0.603422	**0.312**	-6.8743	2	23.74859	7.658158	**0.017**	-7.00064	2	22.00128	0	**0.461**	-5.70144	2	21.40288	0.799294	**0.267**
Stasis	-3.55954	2	15.11909	0	**0.421**	-4.51288	2	19.02575	2.935321	**0.184**	-7.04523	2	22.09046	0.089173	**0.441**	-5.30179	2	20.60359	0	**0.398**
Strict Stasis	-6.55205	1	16.1041	0.985012	**0.257**	-6.37855	1	16.09043	0	**0.799**	-11.0737	1	25.1475	3.146212	**0.096**	-8.80477	1	20.94288	0.339292	**0.336**
Punctuated Equilibrium	-1.78377	4	51.56755	36.44846	**0**	-3.6241	4	Inf	Inf	**0**	-4.4799	4	56.95981	34.95852	**0**	-4.30899	4	Inf	Inf	**0**
Ornstein-Uhlenbeck	-0.92118	4	49.84236	34.72328	**0**	-4.30731	4	Inf	Inf	**0**	-5.80773	4	59.61545	37.61417	**0**	-2.561	4	Inf	Inf	**0**
	**Centroid size**					**Centroid size**					**Centroid size**					**Centroid size**				
General Random Walk	-13.4288	3	44.85759	13.13519	**0.001**	-6.80556	3	43.61112	19.86591	**0**	-12.5039	3	43.00789	10.05333	**0.003**	-6.05424	3	42.10848	25.58286	**0**
Unbiased Random Walk	-13.4921	2	34.98429	3.261885	**0.163**	-9.50422	2	29.00843	5.263226	**0.064**	-12.5622	2	33.12442	0.169864	**0.477**	-6.60691	2	23.21381	6.688189	**0.033**
Stasis	-11.8612	2	31.7224	0	**0.835**	-9.68358	2	29.36717	5.621962	**0.053**	-12.4773	2	32.95456	0	**0.519**	-6.59615	2	23.19229	6.666671	**0.033**
Strict Stasis	-23.2279	1	49.45577	17.73337	**0**	-10.2059	1	23.7452	0	**0.883**	-52.5864	1	108.1728	75.21822	**0**	-6.59614	1	16.52562	0	**0.934**
Punctuated Equilibrium	-9.73319	4	67.46638	35.74398	**0**	-6.56901	4	Inf	Inf	**0**	-7.38	4	62.75999	29.80543	**0**	-5.74171	4	Inf	Inf	**0**
Ornstein-Uhlenbeck	-10.2967	4	68.59332	36.87092	**0**	-6.10603	4	Inf	Inf	**0**	-11.2979	4	70.59587	37.64131	**0**	-5.3996	4	Inf	Inf	**0**

Table S6 - Sample size of the OTUs for each of the datasets analyzed.

	Neurocranial analyses				Facial analyses			
**OTU**	**Homo sapiens lineage**	**Homo neanderthalensis lineage**	**Minimium percentage of missing values**	**Maximum percentage of missing values**	**Homo sapiens lineage**	**Homo neanderthalensis lineage**	**Minimium percentage of missing values**	**Maximum percentage of missing values**
early *Homo*	2	2	9.52	19.05	2	2	13.04	17.39
H. erectus	6	6	3.17	9.52	3	3	20.29	34.78
H. heidelbergensis s.l.	5	5	4.76	19.05	6	6	5.8	21.74
early *H. neanderthalensis*	0	3	14.29	28.57	0	4	26.09	30.43
H. neanderthalensis	0	7	0	0	0	7	8.07	17.39
early *H. sapiens*	8	0	4.17	28.57	5	0	12.17	30.43
Upper Paleolithic *H. sapiens*	26	0	3.66	19.05	20	0	3.04	13.04
H. sapiens	24	0	0	0	24	0	0	0

Table S7 - Model comparison results for the four Principal Components and centroid sizes tested in the four datasets analyzed for alternative Scenario A.

	Neurocranial Analysis - Homo sapiens lineage					Neurocranial Analysis - Homo neanderthalensis lineage					Facial Analysis - Homo sapiens lineage					Facial Analysis - Homo neanderthalensis lineage				
Model	logL	K	AICc	dAICc	**Akaike weight**	logL	K	AICc	dAICc	**Akaike weight**	logL	K	AICc	dAICc	**Akaike weight**	logL	K	AICc	dAICc	**Akaike weight**
	**Principal Component 1**					**Principal Component 1**					**Principal Component 1**					**Principal Component 1**				
General Random Walk	-2.64586	3	35.29172	18.19455	**0**	1.311474	3	Inf	Inf	**0**	-3.81637	3	37.63274	16.61691	**0**	-1.91438	3	Inf	Inf	**0**
Unbiased Random Walk	-3.54859	2	17.09717	0	**0.969**	-2.75076	2	21.50152	56.1427	**0**	-5.53325	2	21.0665	0.050669	**0.494**	-4.40889	2	24.81778	53.01035	**0**
Stasis	-7.00893	2	24.01785	6.920681	**0.03**	-4.85681	2	25.71362	60.3548	**0**	-5.50792	2	21.01583	0	**0.506**	-3.8683	2	23.73659	51.92916	**0**
Strict Stasis	-91.8554	1	187.0441	169.9469	**0**	-29.3509	1	62.70171	97.34289	**0**	-17.7393	1	38.81191	17.79607	**0**	-4.86912	1	13.73825	41.93082	**0**
Punctuated Equilibrium	-1.53256	4	Inf	Inf	**0**	-3.07999	4	-25.84	8.801154	**0.012**	-2.11433	4	Inf	Inf	**0**	-3.00791	4	-25.9842	2.208385	**0.249**
Ornstein-Uhlenbeck	-2.64714	4	Inf	Inf	**0**	1.320591	4	-34.6412	0	**0.988**	-2.78019	4	Inf	Inf	**0**	-1.90371	4	-28.1926	0	**0.751**
	**Principal Component 2**					**Principal Component 2**					**Principal Component 2**					**Principal Component 2**				
General Random Walk	-3.07216	3	36.14432	19.3629	**0**	-4.08128	3	Inf	Inf	**0**	-2.67338	3	35.34676	25.54392	**0**	-2.00391	3	Inf	Inf	**0**
Unbiased Random Walk	-3.39071	2	16.78141	0	**0.614**	-4.09039	2	24.18078	51.90503	**0**	-3.29793	2	16.59585	6.793012	**0.031**	-3.77413	2	23.54825	56.24649	**0**
Stasis	-4.85673	2	19.71346	2.932049	**0.142**	-2.68715	2	21.3743	49.09855	**0**	-3.222	2	16.44401	6.641165	**0.034**	-4.75029	2	25.50057	58.19881	**0**
Strict Stasis	-7.64404	1	18.62142	1.840008	**0.245**	-3.44142	1	10.88283	38.60708	**0**	-3.23476	1	9.802843	0	**0.935**	-6.84168	1	17.68337	50.38161	**0**
Punctuated Equilibrium	-4.78487	4	Inf	Inf	**0**	-2.65248	4	-26.695	1.029213	**0.374**	-3.12853	4	Inf	Inf	**0**	-4.36389	4	-23.2722	9.426016	**0.009**
Ornstein-Uhlenbeck	-0.78392	4	Inf	Inf	**0**	-2.13788	4	-27.7243	0	**0.626**	-2.11951	4	Inf	Inf	**0**	0.34912	4	-32.6982	0	**0.991**
	**Principal Component 3**					**Principal Component 3**					**Principal Component 3**					**Principal Component 3**				
General Random Walk	-3.0511	3	36.1022	21.4553	**0**	-5.58204	3	Inf	Inf	**0**	-5.87561	3	41.75121	20.3157	**0**	NA				
Unbiased Random Walk	-4.49205	2	18.9841	4.337195	**0.085**	-5.58971	2	27.17942	53.32017	**0**	-6.07817	2	22.15634	0.720821	**0.411**	NA				
Stasis	-2.32345	2	14.6469	0	**0.742**	-3.59393	2	23.18786	49.32862	**0**	-5.71776	2	21.43552	0	**0.589**	NA				
Strict Stasis	-7.10883	1	17.55099	2.904086	**0.174**	-5.04535	1	14.0907	40.23146	**0**	-67.1122	1	137.5578	116.1223	**0**	NA				
Punctuated Equilibrium	-1.01718	4	Inf	Inf	**0**	-2.92962	4	-26.1408	0	**0.547**	-1.10172	4	Inf	Inf	**0**	NA				
Ornstein-Uhlenbeck	-2.0672	4	Inf	Inf	**0**	-3.11944	4	-25.7611	0.37964	**0.453**	-4.46151	4	Inf	Inf	**0**	NA				

	Principal Component 4					Principal Component 4					Principal Component 4					Principal Component 4				
General Random Walk	-1.97986	3	33.95972	26.7394	**0**	0.401528	3	Inf	Inf	**0**	-5.84885	3	41.69769	20.48805	**0**	-1.57593	3	Inf	Inf	**0**
Unbiased Random Walk	-1.99549	2	13.99097	6.770649	**0.031**	-0.09249	2	16.18499	49.37129	**0**	-6.05193	2	22.10387	0.894222	**0.26**	-1.92877	2	19.85755	49.36066	**0**
Stasis	-1.57672	2	13.15343	5.933111	**0.047**	-0.0607	2	16.1214	49.30771	**0**	-5.60482	2	21.20964	0	**0.407**	-1.91799	2	19.83599	49.3391	**0**
Strict Stasis	-1.9435	1	7.220324	0	**0.921**	-0.06068	1	4.121361	37.30767	**0**	-9.1387	1	21.61072	0.40108	**0.333**	-1.91799	1	7.835981	37.33909	**0**
Punctuated Equilibrium	-0.91989	4	Inf	Inf	**0**	0.09144	4	-32.1829	1.003425	**0.377**	-4.18066	4	Inf	Inf	**0**	-1.24844	4	-29.5031	0	**0.579**
Ornstein-Uhlenbeck	-1.46635	4	Inf	Inf	**0**	0.593152	4	-33.1863	0	**0.623**	-3.6961	4	Inf	Inf	**0**	-1.56592	4	-28.8682	0.63496	**0.421**
	**Centroid size**					**Centroid size**					**Centroid size**					**Centroid size**				
General Random Walk	-10.3902	3	50.78045	23.76248	**0**	-4.61141	3	Inf	Inf	**0**	-9.83554	3	49.67108	19.62283	**0**	-3.90667	3	Inf	Inf	**0**
Unbiased Random Walk	-10.3957	2	30.79139	3.773427	**0.132**	-6.62723	2	29.25446	52.69325	**0**	-10.0241	2	30.04826	0	**0.616**	-3.92533	2	23.85066	48.64967	**0**
Stasis	-8.50898	2	27.01797	0	**0.868**	-6.18265	2	28.36529	51.80408	**0**	-10.4974	2	30.99482	0.946562	**0.384**	-3.91602	2	23.83204	48.63105	**0**
Strict Stasis	-19.5785	1	42.49038	15.47241	**0**	-6.46319	1	16.92638	40.36517	**0**	-50.9135	1	105.1603	75.11207	**0**	-3.91602	1	11.83204	36.63105	**0**
Punctuated Equilibrium	-8.19779	4	Inf	Inf	**0**	-6.04976	4	-19.9005	3.538296	**0.146**	-5.15977	4	Inf	Inf	**0**	-3.6005	4	-24.799	0	**0.54**
Ornstein-Uhlenbeck	-7.34895	4	Inf	Inf	**0**	-4.28061	4	-23.4388	0	**0.854**	-9.14866	4	Inf	Inf	**0**	-3.75997	4	-24.4801	0.31894	**0.46**

Table S8 - Model comparison results for the four Principal Components and centroid sizes tested in the four datasets analyzed for alternative Scenario B.

	**Neurocranial Analysis - Homo sapiens lineage**					**Neurocranial Analysis - Homo neanderthalensis lineage**					**Facial Analysis - Homo sapiens lineage**					**Facial Analysis - Homo neanderthalensis lineage**				
Model	logL	K	AICc	dAICc	**Akaike weight**	logL	K	AICc	dAICc	**Akaike weight**	logL	K	AICc	dAICc	**Akaike weight**	logL	K	AICc	dAICc	**Akaike weight**
	**Principal Component 1**					**Principal Component 1**					**Principal Component 1**					**Principal Component 1**				
General Random Walk	-3.585481	3	25.17096	6.805233	**0.032**	-0.8823501	3	31.7647	11.377513	**0.003**	-4.764861	3	27.52972	4.042749	**0.079**	-7.909955	3	45.81991	24.545601	**0**
Unbiased Random Walk	-5.182865	2	18.36573	0	**0.951**	-5.1935935	2	20.38719	0	**0.947**	-7.743486	2	23.48697	0	**0.593**	-8.114694	2	26.22939	4.955079	**0.068**
Stasis	-9.201186	2	26.40237	8.036643	**0.017**	-8.1390755	2	26.27815	5.890964	**0.05**	-8.335998	2	24.672	1.185023	**0.328**	-7.560909	2	25.12182	3.847509	**0.119**
Strict Stasis	-125.180262	1	253.36052	234.994794	**0**	-62.2360006	1	127.80533	107.418148	**0**	-19.286035	1	41.57207	18.085097	**0**	-8.970488	1	21.27431	0	**0.813**
Punctuated Equilibrium	-3.113674	4	54.22735	35.861617	**0**	-3.5980693	4	Inf	Inf	**0**	-4.405536	4	56.81107	33.324098	**0**	-6.640697	4	Inf	Inf	**0**
Ornstein-Uhlenbeck	-3.589524	4	55.17905	36.813319	**0**	0.8606699	4	Inf	Inf	**0**	-5.189738	4	58.37948	34.892503	**0**	-1.128282	4	Inf	Inf	**0**
	**Principal Component 2**					**Principal Component 2**					**Principal Component 2**					**Principal Component 2**				
General Random Walk	-7.488687	3	32.97737	9.203625	**0.009**	-6.670858	3	43.34172	25.33726	**0**	-4.143415	3	26.28683	13.583372	**0.001**	-0.4602687	3	30.92054	11.003918	**0.003**
Unbiased Random Walk	-7.886875	2	23.77375	0	**0.858**	-6.685766	2	23.37153	5.367076	**0.05**	-4.922644	2	17.84529	5.141829	**0.066**	-4.9583096	2	19.91662	0	**0.841**
Stasis	-9.841408	2	27.68282	3.909067	**0.121**	-5.237511	2	20.47502	2.470567	**0.214**	-4.8376	2	17.6752	4.971742	**0.072**	-6.6593399	2	23.31868	3.402061	**0.153**
Strict Stasis	-14.630611	1	32.26122	8.487471	**0.012**	-7.335561	1	18.00446	0	**0.736**	-4.851729	1	12.70346	0	**0.861**	-14.1548149	1	31.64296	11.726344	**0.002**
Punctuated Equilibrium	-7.072959	4	62.14592	38.372168	**0**	-4.903853	4	Inf	Inf	**0**	-3.67673	4	55.35346	42.650001	**0**	-1.8095586	4	Inf	Inf	**0**
Ornstein-Uhlenbeck	-3.168744	4	54.33749	30.563738	**0**	-3.75443	4	Inf	Inf	**0**	-4.062677	4	56.12535	43.421896	**0**	-0.4410469	4	Inf	Inf	**0**
	**Principal Component 3**					**Principal Component 3**					**Principal Component 3**					**Principal Component 3**				
General Random Walk	-4.447433	3	26.89487	12.419548	**0.002**	-4.643696	3	39.28739	26.418645	**0**	-9.582121	3	37.16424	10.4236478	**0.003**	-4.606814	3	39.21363	25.422721	**0**
Unbiased Random Walk	-5.832074	2	19.66415	5.188829	**0.062**	-4.855949	2	19.7119	6.843152	**0.028**	-9.614407	2	27.22881	0.4882206	**0.438**	-5.251781	2	20.50356	6.712655	**0.031**
Stasis	-3.237659	2	14.47532	0	**0.824**	-3.512377	2	17.02475	4.156008	**0.108**	-9.370297	2	26.74059	0	**0.559**	-4.324582	2	18.64916	4.858258	**0.078**
Strict Stasis	-7.724409	1	18.44882	3.973499	**0.113**	-4.767706	1	12.86875	0	**0.864**	-90.723506	1	184.44701	157.7064176	**0**	-5.228787	1	13.79091	0	**0.89**
Punctuated Equilibrium	-1.970557	4	51.94111	37.465794	**0**	-3.496014	4	Inf	Inf	**0**	-8.780396	4	65.56079	38.8201984	**0**	-2.99939	4	Inf	Inf	**0**
Ornstein-Uhlenbeck	-3.183893	4	54.36779	39.892467	**0**	-2.134763	4	Inf	Inf	**0**	-5.914794	4	59.82959	33.0889946	**0**	-4.049781	4	Inf	Inf	**0**

	Principal Component 4					Principal Component 4					Principal Component 4					Principal Component 4				
General Random Walk	-3.592742	3	25.18548	15.071299	**0**	-4.486515	3	38.97303	25.25822	**0**	-7.443415	3	32.88683	11.439869	**0.002**	-3.529365	3	37.05873	25.234802	**0**
Unbiased Random Walk	-3.620619	2	15.24124	5.127053	**0.064**	-5.207422	2	20.41484	6.700033	**0.032**	-7.443499	2	22.887	1.440038	**0.24**	-4.273853	2	18.54771	6.723777	**0.032**
Stasis	-3.11724	2	14.23448	4.120294	**0.106**	-4.571803	2	19.14361	5.428795	**0.06**	-6.72348	2	21.44696	0	**0.493**	-4.228005	2	18.45601	6.632081	**0.034**
Strict Stasis	-3.557093	1	10.11419	0	**0.83**	-5.190739	1	13.71481	0	**0.908**	-9.843752	1	22.6875	1.240544	**0.265**	-4.245298	1	11.82393	0	**0.934**
Punctuated Equilibrium	-2.470977	4	52.94195	42.827768	**0**	-3.693892	4	Inf	Inf	**0**	-4.248584	4	56.49717	35.050208	**0**	-3.893203	4	Inf	Inf	**0**
Ornstein-Uhlenbeck	-2.516338	4	53.03268	42.918491	**0**	-4.46788	4	Inf	Inf	**0**	-6.192206	4	60.38441	38.937452	**0**	-3.044149	4	Inf	Inf	**0**
	**Centroid size**					**Centroid size**					**Centroid size**					**Centroid size**				
General Random Walk	-12.821195	3	43.64239	14.210656	**0.001**	-6.231582	3	42.46316	21.605924	**0**	-11.665626	3	41.33125	9.6769287	**0.005**	NA				
Unbiased Random Walk	-12.833135	2	33.66627	4.234535	**0.107**	-8.612142	2	27.22428	6.367044	**0.038**	-11.827162	2	31.65432	0	**0.614**	NA				
Stasis	-10.715867	2	29.43173	0	**0.892**	-8.411578	2	26.82316	5.965916	**0.046**	-12.30441	2	32.60882	0.9544957	**0.381**	NA				
Strict Stasis	-21.932586	1	46.86517	17.433437	**0**	-8.761953	1	20.85724	0	**0.916**	-54.370506	1	111.74101	80.0866869	**0**	NA				
Punctuated Equilibrium	-9.014697	4	66.02939	36.597659	**0**	-5.905099	4	Inf	Inf	**0**	-6.303181	4	60.60636	28.952038	**0**	NA				
Ornstein-Uhlenbeck	-10.094479	4	68.18896	38.757224	**0**	-6.082105	4	Inf	Inf	**0**	-10.740801	4	69.4816	37.8272772	**0**	NA				

Table S9 - Model comparison results for the four Principal Components and centroid sizes tested in the four datasets analyzed for alternative Scenario C.

	**Neurocranial Analysis - Homo sapiens lineage**					**Neurocranial Analysis - Homo neanderthalensis lineage**					**Facial Analysis - Homo sapiens lineage**					**Facial Analysis - Homo neanderthalensis lineage**				
Model	logL	K	AICc	dAICc	**Akaike weight**	logL	K	AICc	dAICc	**Akaike weight**	logL	K	AICc	dAICc	**Akaike weight**	logL	K	AICc	dAICc	**Akaike weight**
	**Principal Component 1**					**Principal Component 1**					**Principal Component 1**					**Principal Component 1**				
General Random Walk	-3.956252	3	25.9125	7.403152	**0.023**	1.452319	3	27.09536	8.873461	**0.011**	-5.307035	3	28.61407	7.0423385	**0.018**	-2.153486	3	34.30697	16.152946	**0**
Unbiased Random Walk	-5.254676	2	18.50935	0	**0.951**	-4.110951	2	18.2219	0	**0.934**	-7.24979	2	22.49958	0.9278492	**0.379**	-5.486027	2	20.97205	2.818029	**0.173**
Stasis	-8.889958	2	25.77992	7.270565	**0.025**	-6.944458	2	23.88892	5.667015	**0.055**	-6.785865	2	21.57173	0	**0.603**	-5.845116	2	21.69023	3.536208	**0.121**
Strict Stasis	-107.479575	1	217.95915	199.449799	**0**	-38.230481	1	79.79429	61.572393	**0**	-20.162984	1	43.32597	21.7542377	**0**	-7.410346	1	18.15402	0	**0.707**
Punctuated Equilibrium	-2.426927	4	52.85385	34.344502	**0**	-1.674687	4	Inf	Inf	**0**	-2.932041	4	53.86408	32.2923521	**0**	-2.874586	4	Inf	Inf	**0**
Ornstein-Uhlenbeck	-3.958492	4	55.91698	37.407634	**0**	1.474807	4	Inf	Inf	**0**	-6.059047	4	60.11809	38.5463628	**0**	-2.130088	4	Inf	Inf	**0**
	**Principal Component 2**					**Principal Component 2**					**Principal Component 2**					**Principal Component 2**				
General Random Walk	-4.97333	3	27.94666	9.381776	**0.005**	NA					-3.847354	3	25.69471	14.774285	**0.001**	-3.549893	3	37.09979	19.573584	**0**
Unbiased Random Walk	-5.282442	2	18.56488	0	**0.547**	NA					-4.041481	2	16.08296	5.162538	**0.065**	-5.299083	2	20.59817	3.071965	**0.158**
Stasis	-6.485279	2	20.97056	2.405674	**0.164**	NA					-3.96022	2	15.92044	5.000016	**0.071**	-5.668429	2	21.33686	3.810656	**0.109**
Strict Stasis	-8.439816	1	19.87963	1.314748	**0.284**	NA					-3.960212	1	10.92042	0	**0.863**	-7.096434	1	17.5262	0	**0.733**
Punctuated Equilibrium	-2.885062	4	53.77012	35.205241	**0**	NA					-3.417165	4	54.83433	43.913906	**0**	-1.402997	4	Inf	Inf	**0**
Ornstein-Uhlenbeck	-3.211468	4	54.42294	35.858051	**0**	NA					-3.713688	4	55.42738	44.506952	**0**	-2.831789	4	Inf	Inf	**0**
	**Principal Component 3**					**Principal Component 3**					**Principal Component 3**					**Principal Component 3**				
General Random Walk	-6.230683	3	30.46137	15.035404	**0**	-7.642624	3	45.28525	26.509412	**0**	-7.444195	3	32.88839	10.671026	**0.003**	-3.325862	3	36.65172	26.654625	**0**
Unbiased Random Walk	-6.515412	2	21.03082	5.604862	**0.056**	-7.648394	2	25.29679	6.520952	**0.023**	-7.824068	2	23.64814	1.430771	**0.327**	-3.34875	2	16.6975	6.7004	**0.033**
Stasis	-3.712981	2	15.42596	0	**0.917**	-4.907649	2	19.8153	1.039462	**0.364**	-7.108682	2	22.21736	0	**0.669**	-3.331887	2	16.66377	6.666674	**0.033**
Strict Stasis	-9.753378	1	22.50676	7.080794	**0.027**	-7.721252	1	18.77584	0	**0.612**	-48.261616	1	99.52323	77.305867	**0**	-3.331883	1	9.9971	0	**0.934**
Punctuated Equilibrium	-2.422621	4	52.84524	37.41928	**0**	-4.341048	4	Inf	Inf	**0**	-2.568617	4	53.13723	30.919869	**0**	-3.022851	4	Inf	Inf	**0**
Ornstein-Uhlenbeck	-3.463899	4	54.9278	39.501835	**0**	-4.656548	4	Inf	Inf	**0**	-5.901136	4	59.80227	37.584907	**0**	-2.599629	4	Inf	Inf	**0**

	Principal Component 4					Principal Component 4					Principal Component 4					Principal Component 4				
General Random Walk	-3.010358	3	24.020716	15.090594	**0**	-1.159654	3	32.319308	25.615654	**0**	-6.830637	3	31.66127	9.557436	**0.006**	-2.781395	3	35.56279	25.870648	**0**
Unbiased Random Walk	-3.02731	2	14.05462	5.124498	**0.065**	-1.7131858	2	13.426372	6.722717	**0.032**	-7.051919	2	22.10384	0	**0.67**	-3.192274	2	16.384548	6.692406	**0.033**
Stasis	-2.741559	2	13.483119	4.552996	**0.087**	-1.6851743	2	13.370349	6.666694	**0.033**	-7.9215	2	23.843	1.739162	**0.281**	-3.179406	2	16.358811	6.666669	**0.033**
Strict Stasis	-2.965061	1	8.930122	0	**0.847**	-1.6851604	1	6.703654	0	**0.934**	-12.301901	1	27.6038	5.499965	**0.043**	-3.179404	1	9.692142	0	**0.934**
Punctuated Equilibrium	-2.125768	4	52.251536	43.321414	**0**	-0.9743775	4	Inf	Inf	**0**	-5.367229	4	58.73446	36.630621	**0**	-2.2638	4	Inf	Inf	**0**
Ornstein-Uhlenbeck	-2.642529	4	53.285058	44.354936	**0**	-1.0803625	4	Inf	Inf	**0**	-5.326131	4	58.65226	36.548425	**0**	-2.768841	4	Inf	Inf	**0**
	**Centroid size**					**Centroid size**					**Centroid size**					**Centroid size**				
General Random Walk	-13.66249	3	45.32498	9.631531	**0.005**	-8.276043	3	46.55209	14.856097	**0**	-12.68625	3	43.3725	9.9994081	**0.004**	-7.346718	3	44.69344	21.690842	**0**
Unbiased Random Walk	-13.846724	2	35.69345	0	**0.639**	-11.026499	2	32.053	0.357008	**0.386**	-12.68654	2	33.37309	0	**0.574**	-9.781522	2	29.56304	6.56045	**0.035**
Stasis	-14.433676	2	36.86735	1.173903	**0.355**	-11.956163	2	33.91233	2.216336	**0.152**	-12.99255	2	33.9851	0.6120129	**0.422**	-9.801522	2	29.60304	6.600449	**0.034**
Strict Stasis	-30.546117	1	64.09223	28.398786	**0**	-14.181328	1	31.69599	0	**0.461**	-52.76026	1	108.52051	75.1474251	**0**	-9.834631	1	23.0026	0	**0.931**
Punctuated Equilibrium	-11.13153	4	70.26306	34.569612	**0**	-8.246495	4	Inf	Inf	**0**	-11.3235	4	70.64699	37.2739036	**0**	-6.890603	4	Inf	Inf	**0**
Ornstein-Uhlenbeck	-9.890604	4	67.78121	32.087759	**0**	-5.911439	4	Inf	Inf	**0**	-10.78614	4	69.57229	36.1991971	**0**	-5.183312	4	Inf	Inf	**0**

Table S10 - Model comparison results for the four Principal Components and centroid sized tested in the four datasets analyzed for alternative Scenario D.

	**Neurocranial Analysis - Homo sapiens lineage**					**Facial Analysis - Homo sapiens lineage**				
Model	logL	K	AICc	dAICc	**Akaike weight**	logL	K	AICc	dAICc	**Akaike weight**
	**Principal Component 1**					**Principal Component 1**				
General Random Walk	-1.49778	3	32.99556	16.58121	**0**	-2.95961	3	35.91923	16.37802	**0**
Unbiased Random Walk	-3.20717	2	16.41435	0	**0.974**	-4.7706	2	19.5412	0	**0.747**
Stasis	-6.82172	2	23.64344	7.229098	**0.026**	-5.85177	2	21.70355	2.162341	**0.253**
Strict Stasis	-140.071	1	283.4747	267.0603	**0**	-17.9565	1	39.24629	19.70509	**0**
Punctuated Equilibrium	-2.99803	4	Inf	Inf	**0**	-1.10853	4	Inf	Inf	**0**
Ornstein-Uhlenbeck	-1.50261	4	Inf	Inf	**0**	-3.22542	4	Inf	Inf	**0**
	**Principal Component 2**					**Principal Component 2**				
General Random Walk	-5.72444	3	41.44888	22.74751	**0**	-4.57492	3	39.14984	21.85289	**0**
Unbiased Random Walk	-5.81429	2	21.62858	2.927208	**0.15**	-4.75228	2	19.50455	2.207599	**0.197**
Stasis	-5.52337	2	21.04674	2.345369	**0.201**	-3.64848	2	17.29695	0	**0.593**
Strict Stasis	-7.68402	1	18.70137	0	**0.649**	-8.02016	1	19.37365	2.076693	**0.21**
Punctuated Equilibrium	-4.23699	4	Inf	Inf	**0**	-3.1108	4	Inf	Inf	**0**
Ornstein-Uhlenbeck	-3.60475	4	Inf	Inf	**0**	-2.16014	4	Inf	Inf	**0**
	**Principal Component 3**					**Principal Component 3**				
General Random Walk	-2.13219	3	34.26437	25.25658	**0**	-2.14699	3	34.29398	14.83674	**0**
Unbiased Random Walk	-2.98418	2	15.96836	6.960562	**0.028**	-4.98779	2	19.97557	0.518334	**0.291**
Stasis	-2.27805	2	14.5561	5.548305	**0.057**	-4.85597	2	19.71193	0.254692	**0.332**
Strict Stasis	-2.83723	1	9.007798	0	**0.915**	-8.06195	1	19.45724	0	**0.377**
Punctuated Equilibrium	0.283433	4	Inf	Inf	**0**	-1.62325	4	Inf	Inf	**0**
Ornstein-Uhlenbeck	-1.83276	4	Inf	Inf	**0**	-2.12434	4	Inf	Inf	**0**

	Principal Component 4					Principal Component 4				
General Random Walk	0.671866	3	28.65627	21.90779	**0**	-5.34964	3	40.69929	20.95613	**0**
Unbiased Random Walk	-1.55066	2	13.10132	6.352842	**0.039**	-5.54528	2	21.09055	1.347395	**0.257**
Stasis	-1.55016	2	13.10032	6.351843	**0.039**	-5.6223	2	21.2446	1.501446	**0.238**
Strict Stasis	-1.70757	1	6.748474	0	**0.923**	-8.20491	1	19.74316	0	**0.505**
Punctuated Equilibrium	0.983266	4	Inf	Inf	**0**	-3.21799	4	Inf	Inf	**0**
Ornstein-Uhlenbeck	1.345545	4	Inf	Inf	**0**	-3.8545	4	Inf	Inf	**0**
	**Centroid size**					**Centroid size**				
General Random Walk	-6.45294	3	42.90588	19.03694	**0**	-11.4576	3	52.91526	23.04034	**0**
Unbiased Random Walk	-9.1632	2	28.3264	4.457463	**0.094**	-11.4833	2	32.96666	3.091748	**0.176**
Stasis	-10.1453	2	30.29062	6.421683	**0.035**	-9.93746	2	29.87492	0	**0.824**
Strict Stasis	-10.2678	1	23.86894	0	**0.871**	-34.9299	1	73.1931	43.31818	**0**
Punctuated Equilibrium	-5.44717	4	Inf	Inf	**0**	-8.94172	4	Inf	Inf	**0**
Ornstein-Uhlenbeck	-5.03021	4	Inf	Inf	**0**	-9.53169	4	Inf	Inf	**0**

Table S11 - Model comparison results for the four Principal Components and centroid sizes tested in the four datasets analyzed for alternative Scenario E.

	**Neurocranial Analysis - Homo neanderthalensis lineage**					**Facial Analysis - Homo neanderthalensis lineage**				
Model	logL	K	AICc	dAICc	**Akaike weight**	logL	K	AICc	dAICc	**Akaike weight**
	**Principal Component 1**					**Principal Component 1**				
General Random Walk	2.316302	3	Inf	Inf	**0**	NA				
Unbiased Random Walk	-3.804	2	23.60801	60.57026	**0**	NA				
Stasis	-5.74491	2	27.48981	64.45207	**0**	NA				
Strict Stasis	-44.8494	1	93.69873	130.661	**0**	NA				
Punctuated Equilibrium	-0.89759	4	-30.2048	6.757443	**0.033**	NA				
Ornstein-Uhlenbeck	2.481127	4	-36.9623	0	**0.967**	NA				
	**Principal Component 2**					**Principal Component 2**				
General Random Walk	NA					1.325269	3	Inf	Inf	**0**
Unbiased Random Walk	NA					-3.24779	2	22.49559	57.52259	**0**
Stasis	NA					-5.56059	2	27.12118	62.14819	**0**
Strict Stasis	NA					-21.7292	1	47.45837	82.48537	**0**
Punctuated Equilibrium	NA					0.995744	4	-33.9915	1.035515	**0.373**
Ornstein-Uhlenbeck	NA					1.513502	4	-35.027	0	**0.627**
	**Principal Component 3**					**Principal Component 3**				
General Random Walk	-1.01938	3	Inf	Inf	**0**	-2.03136	3	Inf	Inf	**0**
Unbiased Random Walk	-1.02476	2	18.04951	48.7372	**0**	-2.10231	2	20.20461	48.88414	**0**
Stasis	-0.95731	2	17.91462	48.60231	**0**	-2.08537	2	20.17073	48.85027	**0**
Strict Stasis	-0.95727	1	5.914537	36.60223	**0**	-2.08536	1	8.170726	36.85026	**0**
Punctuated Equilibrium	-0.94842	4	-30.1032	0.584538	**0.427**	-1.66023	4	-28.6795	0	**0.532**
Ornstein-Uhlenbeck	-0.65616	4	-30.6877	0	**0.573**	-1.78749	4	-28.425	0.254503	**0.468**

	Principal Component 4					Principal Component 4				
General Random Walk	-0.7716	3	Inf	Inf	**0**	-1.92564	3	Inf	Inf	**0**
Unbiased Random Walk	-0.95979	2	17.91959	48.41038	**0**	-4.38801	2	24.77602	54.25977	**0**
Stasis	-0.94286	2	17.88572	48.37652	**0**	-4.31919	2	24.63839	54.12213	**0**
Strict Stasis	-0.94285	1	5.885706	36.3765	**0**	-7.32086	1	18.64173	48.12547	**0**
Punctuated Equilibrium	-0.86437	4	-30.2713	0.219529	**0.473**	-3.0411	4	-25.9178	3.565948	**0.144**
Ornstein-Uhlenbeck	-0.7546	4	-30.4908	0	**0.527**	-1.25813	4	-29.4838	0	**0.856**
	**Centroid size**					**Centroid size**				
General Random Walk	-4.76674	3	Inf	Inf	**0**	-5.03632	3	Inf	Inf	**0**
Unbiased Random Walk	-7.54596	2	31.09192	55.30072	**0**	-5.3499	2	26.6998	50.2471	**0**
Stasis	-8.20709	2	32.41417	56.62297	**0**	-5.34037	2	26.68074	50.22805	**0**
Strict Stasis	-8.52013	1	21.04027	45.24907	**0**	-5.34037	1	14.68073	38.22804	**0**
Punctuated Equilibrium	-4.35135	4	-23.2973	0.911499	**0.388**	-4.56879	4	-22.8624	0.684879	**0.415**
Ornstein-Uhlenbeck	-3.8956	4	-24.2088	0	**0.612**	-4.22635	4	-23.5473	0	**0.585**
