# Supplementary material for: Evolutionary drivers of encephalization and facial reduction in the genus Homo
Source: Nat Commun. 2026 Jul 6;17:5625. doi: 10.1038/s41467-026-74739-w (PMC13338430; doi:10.1038/s41467-026-74739-w)
Supplement: Supplementary file 2 — Description of Additional Supplementary Files [file 41467_2026_74739_MOESM2_ESM.pdf]

Description of Additional Supplementary Files

File Name: Supplementary Code 1

Description: Html and Quarto documents with all code, links to external resources, and analyses to permit replication of the results presented in the article.
