# Supplementary material for: Evolutionary drivers of encephalization and facial reduction in the genus Homo
Source: Nat Commun. 2026 Jul 6;17:5625. doi: 10.1038/s41467-026-74739-w (PMC13338430; doi:10.1038/s41467-026-74739-w)
Supplement: Supplementary file 3 — Supplementary Code 1 [file 41467_2026_74739_MOESM3_ESM.zip › Hubbe and Harvati analyses in html format.html]

Paper analyses script


# Paper analyses script

Author

Hubbe and Harvati

Published

March 10, 2026

# Introduction

This Quarto document recreates all the analyses for the the article **Evolutionary drivers of encephalization and facial reduction in the genus *Homo***

A few notes:

1. All analyses and tables for the article are generated here. However, the final images from the publications were compiled from the plots generated here outside of RStudio (in CorelDraw). Therefore, the format of the images in the article will not match the ones generated here.
2. Some exploratory analyses generated here are not reported in the article. However, they are kept in the document to fully demonstrate the workflow of the study.

## 0. Data preparation and exploratory analyses

In this section, the data is prepared for the analyses that are part of the article and general descriptive statistics are calculated.

### a. Load required packages and functions

The analyses rely on packages available in R, and are complemented by a few functions written by Mark Hubbe and made available in github.

```
#Load packages required
library(tidyverse) # for data handling
```

```
── Attaching core tidyverse packages ──────────────────────── tidyverse 2.0.0 ──
✔ dplyr     1.1.4     ✔ readr     2.1.6
✔ forcats   1.0.1     ✔ stringr   1.6.0
✔ ggplot2   4.0.1     ✔ tibble    3.3.1
✔ lubridate 1.9.4     ✔ tidyr     1.3.2
✔ purrr     1.2.1     
── Conflicts ────────────────────────────────────────── tidyverse_conflicts() ──
✖ dplyr::filter() masks stats::filter()
✖ dplyr::lag()    masks stats::lag()
ℹ Use the conflicted package (<http://conflicted.r-lib.org/>) to force all conflicts to become errors
```

```
library(kableExtra) # to create html tables
```

```
Attaching package: 'kableExtra'

The following object is masked from 'package:dplyr':

    group_rows
```

```
library(MASS) # used to calculate Principal Components
```

```
Attaching package: 'MASS'

The following object is masked from 'package:dplyr':

    select
```

```
library(plotly) # used to plot 3D plots
```

```
Attaching package: 'plotly'

The following object is masked from 'package:MASS':

    select

The following object is masked from 'package:ggplot2':

    last_plot

The following object is masked from 'package:stats':

    filter

The following object is masked from 'package:graphics':

    layout
```

```
library(geomorph) # used for processing of geometric morphometric data
```

```
Loading required package: RRPP
Loading required package: rgl
Loading required package: Matrix

Attaching package: 'Matrix'

The following objects are masked from 'package:tidyr':

    expand, pack, unpack
```

```
library(paleoTS) # used for the calculation of the evolutionary models by Hunt

#Additional functions from github
source("https://raw.githubusercontent.com/markhubbe/homininPaleoTS/refs/heads/main/PC%20analysis%20with%20Plot.R")

source("https://raw.githubusercontent.com/markhubbe/homininPaleoTS/refs/heads/main/array2matrix.R")

source("https://raw.githubusercontent.com/markhubbe/homininPaleoTS/refs/heads/main/dists2paleoTS.R")

source("https://raw.githubusercontent.com/markhubbe/homininPaleoTS/refs/heads/main/lm2dists.R")

source("https://raw.githubusercontent.com/markhubbe/homininPaleoTS/refs/heads/main/matrix2array.R")

source("https://raw.githubusercontent.com/markhubbe/homininPaleoTS/refs/heads/main/multiple%20paleoTS.R")

source("https://raw.githubusercontent.com/markhubbe/homininPaleoTS/refs/heads/main/plotGPAplotly.R")

source("https://raw.githubusercontent.com/markhubbe/homininPaleoTS/refs/heads/main/plotly3d%20from%20dataframe.R")
```

### b. Load data for the analyses

The datasets used in the neurocranial and facial analyses are loaded from an online repository.

- The data used here includes 21 landmarks for the neurocranium and 23 landmarks for the face.
- Only individuals with less than 35% of missing values are included in these datasets.
- Data includes 24 modern humans, one male and one female from 12 different populations worldwide.

```
#Load data from github. 

#These are the addresses for the data 
face_url <- "https://raw.githubusercontent.com/markhubbe/homininPaleoTS/refs/heads/main/Facial%20data.csv"

neurocranium_url <- "https://raw.githubusercontent.com/markhubbe/homininPaleoTS/refs/heads/main/Neurocranial%20data.csv"

#Load facial data

face_data <- read_csv(face_url)
```

```
Rows: 71 Columns: 71
── Column specification ────────────────────────────────────────────────────────
Delimiter: ","
chr  (2): Specimen, SpeciesExpanded
dbl (69): Canine-Premolar contact R_X, Canine-Premolar contact R_Y, Canine-P...

ℹ Use `spec()` to retrieve the full column specification for this data.
ℹ Specify the column types or set `show_col_types = FALSE` to quiet this message.
```

```
#Load neurocranium data

neurocranium_data <- read_csv(neurocranium_url)
```

```
Rows: 81 Columns: 65
── Column specification ────────────────────────────────────────────────────────
Delimiter: ","
chr  (2): Specimen, SpeciesExpanded
dbl (63): Inion_X, Inion_Y, Inion_Z, Asterion R_X, Asterion R_Y, Asterion R_...

ℹ Use `spec()` to retrieve the full column specification for this data.
ℹ Specify the column types or set `show_col_types = FALSE` to quiet this message.
```

```
#The analyses require an average chronology for each OTU. This average chrnology is created next manually. Average dates are based on information available on Table S1 

taxa_chronology <- tibble(
  taxon = c("early H. sapiens", "H. erectus", "early Homo", "H. heidelbergensis s.l.", "early H. neanderthalensis", "H. neanderthalensis", "H. sapiens", "Upper Paleolithic H. sapiens" 
  ),
  chronology = c(
    120, 1600, 1900, 450, 150, 50, 0, 27
  )
)
```

### c. Report on missing values per fossil specimen

The information on the percentage of missing values for each fossil specimen, as shown on Table S1 is calculated next.

```
#Calculate missing values' percentage for each fossil in the neurocranial data and report in table
#NOTE: modern Homo sapiens are removed from the table, as they are all complete
neuro_na <- neurocranium_data|>
  filter(
    SpeciesExpanded != "H. sapiens"
  )|>
  pivot_longer(
    cols = 3:last_col(),
    names_to = "variable",
    values_to = "values"
  )|>
  group_by(Specimen)|>
  summarise(
   NA_frequency = sum(is.na(values))/n()*100
  )

neuro_na|>
  kbl(digits=2,caption = "Percentage of missing values  for each fossil specimen in the neurocranial data")|>
  kable_styling(
    bootstrap_options = c("striped", "hover", "condensed"),
    full_width = F)
```

Percentage of missing values for each fossil specimen in the neurocranial data

| Specimen | NA\_frequency |
| --- | --- |
| Abri Pataud | 0.00 |
| Amud 1 | 0.00 |
| Brno 2 | 9.52 |
| Broken Hill (Kabwe) | 0.00 |
| Chancelade | 0.00 |
| Cioclovina | 0.00 |
| Cro Magnon 1 | 0.00 |
| Cro-Magnon 2 | 9.52 |
| Dali | 0.00 |
| Das Es Soltane 5 | 28.57 |
| Dmanini2 | 9.52 |
| Dmanisi1 | 0.00 |
| Dolni Vestonice 13 | 0.00 |
| Dolni Vestonice 15 | 0.00 |
| Dolni Vestonice 16 | 0.00 |
| Dolni Vestonice 3 | 0.00 |
| Gibraltar 1 | 0.00 |
| Grimaldi | 0.00 |
| Guattari 1 | 0.00 |
| Hofmeyr | 14.29 |
| Iwo Eleru | 0.00 |
| Jebel Irhoud 1 | 0.00 |
| Jebel Irhoud 2 | 0.00 |
| KNM ER 1470 | 19.05 |
| KNM ER 1813 | 0.00 |
| KNM ER 3733 | 0.00 |
| KNM ER 3883 | 0.00 |
| KNM WT 15000 | 4.76 |
| Kanalda | 9.52 |
| Krapina 3 | 28.57 |
| LH18 | 0.00 |
| La Chapelle aux Saints | 0.00 |
| La Ferrassie 1 | 0.00 |
| Lau bassa | 9.52 |
| Mladec 1 | 9.52 |
| Mladec 2 | 4.76 |
| Mladec 5 | 0.00 |
| Mladec 6 | 19.05 |
| Ndutu | 4.76 |
| OH9 | 4.76 |
| Oase 2 | 0.00 |
| Ohalo II | 0.00 |
| Omo 1 | 4.76 |
| Pavlov 1 | 0.00 |
| Petralona | 19.05 |
| Predmost 3 | 0.00 |
| Predmost 4 | 0.00 |
| Qafzeh 6 | 0.00 |
| Qafzeh 9 | 0.00 |
| Quina 5 | 0.00 |
| Saccopastore 1 | 14.29 |
| Shanidar 1 | 0.00 |
| Sima Craneo 5 | 0.00 |
| Skhul 5 | 0.00 |
| Tabun C1 | 0.00 |
| Upper Cave 101 | 0.00 |
| Upper Cave 103 | 9.52 |

```
#alculate missing values' percentage for each fossil in the facial data and report in table
#NOTE: modern Homo sapiens are removed from the table, as they are mostly complete
face_na <- face_data|>
  filter(
    SpeciesExpanded != "H. sapiens"
  )|>
  pivot_longer(
    cols = 3:last_col(),
    names_to = "variable",
    values_to = "values"
  )|>
  group_by(Specimen)|>
  summarise(
   NA_frequency = sum(is.na(values))/n()*100
  )
face_na|>
  kbl(digits=2,caption = "Percentage of missing values  for each fossil specimen in  the facial data")|>
  kable_styling(
    bootstrap_options = c("striped", "hover", "condensed"),
    full_width = F)
```

Percentage of missing values for each fossil specimen in the facial data

| Specimen | NA\_frequency |
| --- | --- |
| Abri Pataud | 0.00 |
| Amud 1 | 17.39 |
| Apidima 2 | 17.39 |
| Arago 21 | 0.00 |
| Bodo | 4.35 |
| Broken Hill (Kabwe) | 0.00 |
| Chancelade | 8.70 |
| Cro Magnon 1 | 13.04 |
| Cro-Magnon 2 | 8.70 |
| Dali | 21.74 |
| Das Es Soltane 5 | 21.74 |
| Dmanini2 | 21.74 |
| Dolni Vestonice 13 | 0.00 |
| Dolni Vestonice 15 | 4.35 |
| Dolni Vestonice 16 | 0.00 |
| Dolni Vestonice 3 | 0.00 |
| Gibraltar 1 | 8.70 |
| Grimaldi | 0.00 |
| Guattari 1 | 8.70 |
| Hofmeyr | 8.70 |
| Jebel Irhoud 1 | 8.70 |
| KNM ER 1470 | 17.39 |
| KNM ER 1813 | 8.70 |
| KNM ER 3883 | 34.78 |
| KNM WT 15000 | 4.35 |
| Kanalda | 0.00 |
| Krapina 3 | 26.09 |
| La Chapelle aux Saints | 17.39 |
| La Ferrassie 1 | 0.00 |
| Mladec 1 | 0.00 |
| Mladec 2 | 13.04 |
| Muierii 1 | 0.00 |
| Oase 2 | 0.00 |
| Ohalo II | 0.00 |
| Petralona | 0.00 |
| Predmost 4 | 0.00 |
| Qafzeh 6 | 0.00 |
| Qafzeh 9 | 0.00 |
| Saccopastore 1 | 30.43 |
| Shanidar 1 | 0.00 |
| Shanidar 5 | 4.35 |
| Sima Craneo 5 | 8.70 |
| Skhul 5 | 30.43 |
| Tabun C1 | 30.43 |
| Upper Cave 101 | 0.00 |
| Upper Cave 103 | 4.35 |
| Wadi Kubbaniya | 0.00 |

### d. Estimate missing values

The final step in data preparation is the estimation of missing values. This is done using the procedure inside the `geomorph` package, through the the function `estimate.missing`.

```
#To estimate missing values, the dataframes are converted to an array format, as required by geomorph, and then brought back to data.frame format.

#matrix to array
neuro_array <- matrix2array(neurocranium_data[,c(1,3:ncol(neurocranium_data))]) #column 2, with species label, is removed from this data 

face_array <- matrix2array(face_data[,c(1,3:ncol(face_data))]) #column 2, with species label, is removed from this data 

#estimate missing
neuro_array_nomiss <- estimate.missing(neuro_array)

face_array_nomiss <- estimate.missing(face_array)

#superimpose data using procrustes generalizaed analysis

neuro_gpa <- gpagen(neuro_array_nomiss)
```

```
Performing GPA

  |                                                                            
  |                                                                      |   0%
  |                                                                            
  |==================                                                    |  25%
  |                                                                            
  |===================================                                   |  50%
  |                                                                            
  |======================================================================| 100%

Making projections... Finished!
```

```
face_gpa <- gpagen(face_array_nomiss)
```

```
Performing GPA

  |                                                                            
  |                                                                      |   0%
  |                                                                            
  |==================                                                    |  25%
  |                                                                            
  |===================================                                   |  50%
  |                                                                            
  |====================================================                  |  75%
  |                                                                            
  |======================================================================| 100%

Making projections... Finished!
```

```
#return to dataframe format
neuro_nomiss <- array2matrix(neuro_gpa$coords)
colnames(neuro_nomiss) <- colnames(neurocranium_data)[c(1,3:ncol(neurocranium_data))] #column 2, with species label, is removed from column names, so it matches the array data

face_nomiss <- array2matrix(face_gpa$coords)
colnames(face_nomiss) <- colnames(face_data)[c(1,3:ncol(face_data))] #column 2, with species label, is removed from column names, so it matches the array data
```

## 1 . Article analyses

This section creates the analyses reported in the main article. Principal Components Analyses and the fitting of evolutionary models are done for the neurocranial and facial data for the *Homo sapiens* and *Homo neanderthalensis* lineages.

### a. Neurocranial analyses of the *Homo sapiens* lineage

The analyses presented in this section generate Figure 1 and are compiled with the other analyses to generate Tables 1, S4, and S5. The analyses are separated into Principal Components Analysis, evolutionary model testing, and finally the generation of the wireframes for the extreme values of the Principal Components to illustrate the morphological changes across each axis.

Before the analyses, specimens that are not part of the *H. sapiens* lineage are removed.

```
#create an index of which rows belong to the H. sapiens lineage
sapiens_lineage_index <- !neurocranium_data$SpeciesExpanded %in% c(
      "early H. neanderthalensis", "H. neanderthalensis"
      )

neuro_sapiens_nomiss <- neuro_nomiss|>
  filter( #Filter out species not on H. sapiens lineage
    sapiens_lineage_index
  )
```

#### i. Principal Components Analysis

PCA is used to explore the first four Principal Components through violin plots that are compiled in Figure 1 in the article.

The final violin plot presents the distribution of centroid size of specimens in each OTU, to explore specimen size.

```
#PCA is calculated using an imported custom function written by MH.
PCA_neuro_sapiens <- PCA(neuro_sapiens_nomiss[,2:ncol(neuro_nomiss)],neuro_sapiens_nomiss[,1])

#A dataframe is created to facilitate ploting via ggplot
PCA_neuro_sapiens_plotdf <- tibble(
  Specimen = neuro_sapiens_nomiss$Specimen,
  Species =  factor(neurocranium_data$SpeciesExpanded[sapiens_lineage_index],
                    levels = 
                       c("early Homo", "H. erectus",
                         "H. heidelbergensis s.l.", 
                         "early H. sapiens",
                         "Upper Paleolithic H. sapiens",
                         "H. sapiens")
  ),
  PC1 = PCA_neuro_sapiens$PCS[,1],
  PC2 = PCA_neuro_sapiens$PCS[,2],
  PC3 = PCA_neuro_sapiens$PCS[,3],
  PC4 = PCA_neuro_sapiens$PCS[,4],
  Centroid_size = neuro_gpa$Csize[sapiens_lineage_index]
)

#Plot PC1
ggplot(PCA_neuro_sapiens_plotdf, aes(y = PC1,x=Species))+
  geom_violin(aes(color=Species, fill = Species))+
  scale_colour_viridis_d(option="H")+
  geom_boxplot(width=0.1)+
  scale_fill_viridis_d(alpha=0.3,option="H")+
  labs(x = "PC1")
```

```
#Plot PC2
ggplot(PCA_neuro_sapiens_plotdf, aes(y = PC2,x=Species))+
  geom_violin(aes(color=Species, fill = Species))+
  scale_colour_viridis_d(option="H")+
  geom_boxplot(width=0.1)+
  scale_fill_viridis_d(alpha=0.3,option="H")+
  labs(x = "PC2")
```

```
#Plot PC3
ggplot(PCA_neuro_sapiens_plotdf, aes(y = PC3,x=Species))+
  geom_violin(aes(color=Species, fill = Species))+
  scale_colour_viridis_d(option="H")+
  geom_boxplot(width=0.1)+
  scale_fill_viridis_d(alpha=0.3,option="H")+
  labs(x = "PC3")
```

```
#Plot PC4
ggplot(PCA_neuro_sapiens_plotdf, aes(y = PC4,x=Species))+
  geom_violin(aes(color=Species, fill = Species))+
  scale_colour_viridis_d(option="H")+
  geom_boxplot(width=0.1)+
  scale_fill_viridis_d(alpha=0.3,option="H")+
  labs(x = "PC4")
```

```
#Plot centroid size
ggplot(PCA_neuro_sapiens_plotdf, aes(y = Centroid_size, x=Species))+
  geom_violin(aes(color=Species, fill = Species))+
  scale_colour_viridis_d(option="H")+
  geom_boxplot(width=0.1)+
  scale_fill_viridis_d(alpha=0.3,option="H")+
  labs(x = "Centroid Size")
```

The eigenvalues and percentage of variance explained for this analysis are reported here. This makes up part of Table S4.

```
PCA_neuro_sapiens$eigenvalues|>
  data.frame()|>
  slice(1:4)|>
  dplyr::rename(
    Eigenvalue = eigenvalues,
    `Variance %` = X..of.var,
    `Cumulative eigenvalue` = cum.evalues,
    `Cumulative Variance %` = cum...var
  )|>
  mutate(
    `Variance %` = round(`Variance %`,3),
    `Cumulative Variance %` = round(`Cumulative Variance %`,3)
  )|>
   kbl(caption = "Eigenvalues and variance explained for the neurocranial analysis of the H. sapiens analysis")|>
  kable_styling(
    bootstrap_options = c("striped", "hover", "condensed"),
    full_width = F)
```

Eigenvalues and variance explained for the neurocranial analysis of the H. sapiens analysis

|  | Eigenvalue | Variance % | Cumulative eigenvalue | Cumulative Variance % |
| --- | --- | --- | --- | --- |
| PC 1 | 0.0021177 | 27.585 | 0.0021177 | 27.585 |
| PC 2 | 0.0010838 | 14.118 | 0.0032015 | 41.703 |
| PC 3 | 0.0005632 | 7.336 | 0.0037647 | 49.039 |
| PC 4 | 0.0004855 | 6.325 | 0.0042502 | 55.364 |

#### ii. Evolutionary model testing

Next, we calculate the goodness of fit for different evolutionary models, following the method described in Hunt (2012). The analyses are done using package `EvoTS`, inside a wrapper function (`multiple_paleoTS`), which runs multiple models on the same dataset and generates a summary plot.

The text output from these analyses is presented in the article in Table 1 and Table S5, and the last plot generated is included in Figure 1 of the article.

```
#Create vector with species assignation
species_sapiens <- factor( neurocranium_data$SpeciesExpanded[sapiens_lineage_index]
)
                           
#load chronology data
chronology_sapiens <- taxa_chronology|>
  filter(
    !taxon %in% c(
      "early H. neanderthalensis", "H. neanderthalensis"
      )
  )

#This function prepares the data to be passed to PaleoTS 
PTS_PCS_sapiens <- dists2PaleoTS(data.frame(PCA_neuro_sapiens_plotdf[,c(1,3:7)]),species_sapiens,chronology_sapiens)

#This wrapper function will test the 6 evolutionary models considered in the article against each of the PCs and the centroid size. The final figure output is used to compile Figure 1 in the article.
PTS_PCS_models_sapiens <- multiple_paleoTS(PTS_PCS_sapiens,models=6)
```

```
Total # hypotheses:  3 
1  2  3  

Comparing 6 models [n = 6, method = Joint]

                    logL K      AICc     dAICc Akaike.wt
GRW            -1.636734 3  21.27347   6.56940     0.036
URW            -3.352034 2  14.70407   0.00000     0.961
Stasis         -9.076226 2  26.15245  11.44838     0.003
StrictStasis -265.323227 1 533.64645 518.94239     0.000
Punc-1         -3.291758 4  54.58352  39.87945     0.000
OU             -1.641080 4  51.28216  36.57809     0.000
```

```
Warning in compareModels(grw, urw, sta, strsta, pun2, ou): Optimization for the following model(s) did not converge: OU
These model fit(s) should not be considered reliable.
```

```
Total # hypotheses:  3 
1  2  3  

Comparing 6 models [n = 6, method = Joint]

                  logL K     AICc      dAICc Akaike.wt
GRW          -6.057627 3 30.11525  9.7795569     0.003
URW          -6.167848 2 20.33570  0.0000000     0.416
Stasis       -6.680280 2 21.36056  1.0248640     0.249
StrictStasis -8.896943 1 20.79389  0.4581889     0.331
Punc-1       -5.139925 4 58.27985 37.9441536     0.000
OU           -3.897264 4 55.79453 35.4588320     0.000
```

```
Total # hypotheses:  3 
1  2  3  

Comparing 6 models [n = 6, method = Joint]

                  logL K     AICc     dAICc Akaike.wt
GRW          -5.609644 3 29.21929 15.307976     0.000
URW          -5.662642 2 19.32528  5.413973     0.059
Stasis       -2.955656 2 13.91131  0.000000     0.889
StrictStasis -8.309024 1 19.61805  5.706736     0.051
Punc-1       -1.856833 4 51.71367 37.802354     0.000
OU           -2.557247 4 53.11449 39.203183     0.000
```

```
Total # hypotheses:  3 
1  2  3  

Comparing 6 models [n = 6, method = Joint]

                   logL K     AICc      dAICc Akaike.wt
GRW          -2.3330120 3 22.66602  7.5469347     0.010
URW          -3.8612555 2 15.72251  0.6034218     0.312
Stasis       -3.5595446 2 15.11909  0.0000000     0.421
StrictStasis -6.5520506 1 16.10410  0.9850119     0.257
Punc-1       -1.7837746 4 51.56755 36.4484600     0.000
OU           -0.9211818 4 49.84236 34.7232743     0.000
```

```
Total # hypotheses:  3 
1  2  3  

Comparing 6 models [n = 6, method = Joint]

                   logL K     AICc     dAICc Akaike.wt
GRW          -13.428797 3 44.85759 13.135193     0.001
URW          -13.492143 2 34.98429  3.261885     0.163
Stasis       -11.861200 2 31.72240  0.000000     0.835
StrictStasis -23.227883 1 49.45577 17.733365     0.000
Punc-1        -9.733192 4 67.46638 35.743984     0.000
OU           -10.296659 4 68.59332 36.870917     0.000
```

#### iii. PC wireframes

The final step in the analysis is to create the wireframe visualizations of the the principal components, to illustrate the shape changes over each PCA. The plots generated in this section are compiled in Figure 1 of the article.

```
#Extract the minimum and maximum of each of the four PCs analyzed
PCranges_neuro_sapiens <- data.frame(
  matrix(0,8,ncol(PCA_neuro_sapiens$PCS)+1))# 8 rows because we are getting minimum and maxium for each PC.

#Name the columns of the new dataframe
colnames(PCranges_neuro_sapiens) <- c("PC_limit",colnames(PCA_neuro_sapiens$PCS))

#In this loop, the minimum and maximum values for each PC are extracted from the PC data and scaled by the square root of the eigenvalues, as the first step in rotating the PC coordinate back into the original variables' coordinates
for(a in 1:4){
  
  minPCidx <- which(PCA_neuro_sapiens$PCS[,a]==min(PCA_neuro_sapiens$PCS[,a]))
  
  maxPCidx <- which(PCA_neuro_sapiens$PCS[,a]==max(PCA_neuro_sapiens$PCS[,a]))
  
  PCranges_neuro_sapiens[(a-1)*2+1,a+1] <- PCA_neuro_sapiens$PCS[minPCidx,a]*PCA_neuro_sapiens$eigenvalues[a,1]^0.5
  
  PCranges_neuro_sapiens[(a-1)*2+1,1] <- paste0("minPC",a)
  
  PCranges_neuro_sapiens[(a-1)*2+2,a+1] <- PCA_neuro_sapiens$PCS[maxPCidx,a]*PCA_neuro_sapiens$eigenvalues[a,1]^0.5
  
  PCranges_neuro_sapiens[(a-1)*2+2,1] <- paste0("maxPC",a)
  
}

#The PC coordinates are muliplied by the eigenvectors to complete the rotation to the origninal variable scales.

ranges_neuro_sapiens <- as.matrix(PCranges_neuro_sapiens[,2:ncol(PCranges_neuro_sapiens)])%*%t(PCA_neuro_sapiens$evectors)

means_neuro_sapiens <- apply(neuro_sapiens_nomiss[,2:ncol(neuro_sapiens_nomiss)],2,mean)

ranges_neuro_sapiens <- data.frame(t(apply(ranges_neuro_sapiens,1,function(x){x+means_neuro_sapiens})))

ranges_neuro_sapiens <- cbind(PCranges_neuro_sapiens$PC_limit,ranges_neuro_sapiens)

colnames(ranges_neuro_sapiens)[1] <- colnames(PCranges_neuro_sapiens)[1]

#Next,  the wireframe matrix is created this is a list table that identifies which landmarks are connected.
#1. Get the variable names
neuro_variables_name <- colnames(neuro_sapiens_nomiss)[-1]
neuro_variables_name <- neuro_variables_name[seq(1,length(neuro_variables_name),by=3)]|>
  str_replace("_X","")

#2.create the pairs of variables to be connected
wireframe_neuro <- tribble(
  ~start, ~end,
  "Inion", "Asterion R",
  "Inion", "Asterion L",
  "Inion", "Lambda", 
  "Stylomastoid For. R", "Porion R",
  "Stylomastoid For. L", "Porion L",
  "Porion R", "Lat. Glenoid R",
  "Porion L", "Lat. Glenoid L",
  "Asterion R", "Parietal Notch R",
  "Parietal Notch R", "Mastoidiale R",
  "Mastoidiale R", "Porion R",
  "Asterion L", "Parietal Notch L",
  "Parietal Notch L", "Mastoidiale L",
  "Mastoidiale L", "Porion L",
  "Bregma", "Lambda", 
  "Bregma", "Post-toral sulcus", 
  "Post-toral sulcus", "Glabella",
  "Bregma", "Stephanion R",
  "Stephanion R", "Auriculare R",
  "Auriculare R","Porion R",
  "Bregma", "Stephanion L",
  "Stephanion L", "Auriculare L",
  "Auriculare L", "Porion L"
)

#3. Match them with the variable number to pass as a numerical index to the plot
wireframe_neuro$start_idx<-NA
wireframe_neuro$end_idx<-NA
for(a in 1:nrow(wireframe_neuro)){
  wireframe_neuro$start_idx[a]<-which(neuro_variables_name == wireframe_neuro$start[a])
  wireframe_neuro$end_idx[a]<-which(neuro_variables_name  == wireframe_neuro$end[a])
}

#The rotated coordinates are ploted for each PC.
#1. Setup camera parameters and colors for plots
camera_lat<-list(eye=list(x=0,y=2,z=0))
camera_front<-list(eye=list(x=2,y=0,z=0))
colors_wire<-c("#d16111","#227081") #color for max and min, respectively

#PC1 - lateral view plot
plot3d_df(ranges_neuro_sapiens[1:2,],plot_labels = FALSE,frame=wireframe_neuro[,3:4],color=colors_wire,plot.centroid = FALSE)%>%
layout(scene=list(xaxis=list(visible=F),yaxis=list(visible=F),zaxis=list(visible=F),camera=camera_front))
```

```
#PC1 - frontal view plot
plot3d_df(ranges_neuro_sapiens[1:2,],plot_labels = FALSE,frame=wireframe_neuro[,3:4],color=colors_wire,plot.centroid = FALSE)%>%
layout(scene=list(xaxis=list(visible=F),yaxis=list(visible=F),zaxis=list(visible=F),camera=camera_lat))
```

```
#PC2 - lateral view plot
plot3d_df(ranges_neuro_sapiens[3:4,],plot_labels = FALSE,frame=wireframe_neuro[,3:4],color=colors_wire,plot.centroid = FALSE)%>%
layout(scene=list(xaxis=list(visible=F),yaxis=list(visible=F),zaxis=list(visible=F),camera=camera_front))
```

```
#PC2 - frontal view plot
plot3d_df(ranges_neuro_sapiens[3:4,],plot_labels = FALSE,frame=wireframe_neuro[,3:4],color=colors_wire,plot.centroid = FALSE)%>%
layout(scene=list(xaxis=list(visible=F),yaxis=list(visible=F),zaxis=list(visible=F),camera=camera_lat))
```

```
#PC3 - lateral view plot
plot3d_df(ranges_neuro_sapiens[5:6,],plot_labels = FALSE,frame=wireframe_neuro[,3:4],color=colors_wire,plot.centroid = FALSE)%>%
layout(scene=list(xaxis=list(visible=F),yaxis=list(visible=F),zaxis=list(visible=F),camera=camera_front))
```

```
#PC3 - frontal view plot
plot3d_df(ranges_neuro_sapiens[5:6,],plot_labels = FALSE,frame=wireframe_neuro[,3:4],color=colors_wire,plot.centroid = FALSE)%>%
layout(scene=list(xaxis=list(visible=F),yaxis=list(visible=F),zaxis=list(visible=F),camera=camera_lat))
```

```
#PC4 - lateral view plot
plot3d_df(ranges_neuro_sapiens[7:8,],plot_labels = FALSE,frame=wireframe_neuro[,3:4],color=colors_wire,plot.centroid = FALSE)%>%
layout(scene=list(xaxis=list(visible=F),yaxis=list(visible=F),zaxis=list(visible=F),camera=camera_front))
```

```
#PC4 - frontal view plot
plot3d_df(ranges_neuro_sapiens[7:8,],plot_labels = FALSE,frame=wireframe_neuro[,3:4],color=colors_wire,plot.centroid = FALSE)%>%
layout(scene=list(xaxis=list(visible=F),yaxis=list(visible=F),zaxis=list(visible=F),camera=camera_lat))
```

### b. Neurocranial analyses of the *Homo neanderthalensis* lineage

Following the same structure as the analyses for the *H. sapiens* lineage, the next sub-sections present the analyses for the neurocranial data of the *H. neanderthalensis* lineage. The analyses presented in this section generate Figure 2 and are compiled with the other analyses to generate Tables 1, S4, and S5.

Before the analyses, specimens that are not part of the *H. neanderthalensis* lineage are removed.

```
#create an index of which rows belong to the H. neanderthalensis lineage
neandertal_lineage_index <- !neurocranium_data$SpeciesExpanded %in% c(
      "early H. sapiens", "Upper Paleolithic H. sapiens","H. sapiens"
      )

neuro_neandertal_nomiss <- neuro_nomiss|>
  filter( #Filter out species not on H. neanderthalensis lineage
    neandertal_lineage_index
  )
```

#### i. Principal Components Analysis

PCA is used to explore the first four Principal Components through violin plots that are compiled in Figure 2 of the article.

The final violin plot presents the distribution of centroid size of specimens in each OTU, to explore specimen size.

```
#PCA is calculated using an imported custom function written by MH.
PCA_neuro_neandertal <- PCA(neuro_neandertal_nomiss[,2:ncol(neuro_nomiss)],neuro_neandertal_nomiss[,1])

#A dataframe is created to facilitate ploting via ggplot
PCA_neuro_neandertal_plotdf <- tibble(
  Specimen = neuro_neandertal_nomiss$Specimen,
  Species =  factor(neurocranium_data$SpeciesExpanded[neandertal_lineage_index],
                    levels = 
                       c("early Homo", "H. erectus",
                         "H. heidelbergensis s.l.", 
                         "early H. neanderthalensis",
                         "H. neanderthalensis")
  ),
  PC1 = PCA_neuro_neandertal$PCS[,1],
  PC2 = PCA_neuro_neandertal$PCS[,2],
  PC3 = PCA_neuro_neandertal$PCS[,3],
  PC4 = PCA_neuro_neandertal$PCS[,4],
  Centroid_size = neuro_gpa$Csize[neandertal_lineage_index]
)

#Plot PC1
ggplot(PCA_neuro_neandertal_plotdf, aes(y = PC1,x=Species))+
  geom_violin(aes(color=Species, fill = Species))+
  scale_colour_viridis_d(option="H")+
  geom_boxplot(width=0.1)+
  scale_fill_viridis_d(alpha=0.3,option="H")+
  labs(x = "PC1")
```

```
#Plot PC2
ggplot(PCA_neuro_neandertal_plotdf, aes(y = PC2,x=Species))+
  geom_violin(aes(color=Species, fill = Species))+
  scale_colour_viridis_d(option="H")+
  geom_boxplot(width=0.1)+
  scale_fill_viridis_d(alpha=0.3,option="H")+
  labs(x = "PC2")
```

```
#Plot PC3
ggplot(PCA_neuro_neandertal_plotdf, aes(y = PC3,x=Species))+
  geom_violin(aes(color=Species, fill = Species))+
  scale_colour_viridis_d(option="H")+
  geom_boxplot(width=0.1)+
  scale_fill_viridis_d(alpha=0.3,option="H")+
  labs(x = "PC3")
```

```
#Plot PC4
ggplot(PCA_neuro_neandertal_plotdf, aes(y = PC4,x=Species))+
  geom_violin(aes(color=Species, fill = Species))+
  scale_colour_viridis_d(option="H")+
  geom_boxplot(width=0.1)+
  scale_fill_viridis_d(alpha=0.3,option="H")+
  labs(x = "PC4")
```

```
#Plot centroid size
ggplot(PCA_neuro_neandertal_plotdf, aes(y = Centroid_size, x=Species))+
  geom_violin(aes(color=Species, fill = Species))+
  scale_colour_viridis_d(option="H")+
  geom_boxplot(width=0.1)+
  scale_fill_viridis_d(alpha=0.3,option="H")+
  labs(x = "Centroid Size")
```

The eigenvalues and percentage of variance explained for this analysis are reported here. This makes up part of Table S4.

```
PCA_neuro_neandertal$eigenvalues|>
  data.frame()|>
  slice(1:4)|>
  dplyr::rename(
    Eigenvalue = eigenvalues,
    `Variance %` = X..of.var,
    `Cumulative eigenvalue` = cum.evalues,
    `Cumulative Variance %` = cum...var
  )|>
  mutate(
    `Variance %` = round(`Variance %`,3),
    `Cumulative Variance %` = round(`Cumulative Variance %`,3)
  )|>
   kbl(caption = "Eigenvalues and variance explained for the neurocranial analysis of the H. neanderthalensis analysis.")|>
  kable_styling(
    bootstrap_options = c("striped", "hover", "condensed"),
    full_width = F)
```

Eigenvalues and variance explained for the neurocranial analysis of the H. neanderthalensis analysis.

|  | Eigenvalue | Variance % | Cumulative eigenvalue | Cumulative Variance % |
| --- | --- | --- | --- | --- |
| PC 1 | 0.0028512 | 33.860 | 0.0028512 | 33.860 |
| PC 2 | 0.0011894 | 14.125 | 0.0040406 | 47.985 |
| PC 3 | 0.0007426 | 8.819 | 0.0047832 | 56.804 |
| PC 4 | 0.0007050 | 8.372 | 0.0054882 | 65.176 |

#### ii. Evolutionary model testing

Next, we calculate the goodness of fit for different evolutionary models, as done in the analysis of the *H. sapiens* data.

The text output from this analyses is presented in the article in Table 1 and Table S5, and the last plot generated is included in Figure 2 of the article.

```
#Create vector with species assignation
species_neandertal <- factor( neurocranium_data$SpeciesExpanded[neandertal_lineage_index]
)
                           
#load chronology data
chronology_neandertal <- taxa_chronology|>
  filter(
    !taxon %in% c(
      "early H. sapiens", "Upper Paleolithic H. sapiens","H. sapiens"
      )
  )

#This function prepares the data to be passed to PaleoTS
PTS_PCS_neandertal <- dists2PaleoTS(data.frame(PCA_neuro_neandertal_plotdf[,c(1,3:7)]),species_neandertal,chronology_neandertal)

#This wrapper function will test the 6 evolutionary models considered in the article against each of the PCs and the centroid size. The final figure output is used to compile Figure 2 in the article.
PTS_PCS_models_neandertal <- multiple_paleoTS(PTS_PCS_neandertal,models=6)
```

```
Total # hypotheses:  2 
1  2  

Comparing 6 models [n = 5, method = Joint]

                   logL K     AICc     dAICc Akaike.wt
GRW            2.278425 3 25.44315  7.339272     0.024
URW           -4.051938 2 18.10388  0.000000     0.931
Stasis        -7.082330 2 24.16466  6.060783     0.045
StrictStasis -46.762130 1 96.85759 78.753717     0.000
Punc-1        -1.234952 4      Inf       Inf     0.000
OU             2.367156 4      Inf       Inf     0.000
```

```
Warning in compareModels(grw, urw, sta, strsta, pun2, ou): Optimization for the following model(s) did not converge: OU
These model fit(s) should not be considered reliable.
```

```
Total # hypotheses:  2 
1  2  

Comparing 6 models [n = 5, method = Joint]

                  logL K     AICc     dAICc Akaike.wt
GRW          -6.014745 3 42.02949 26.892209     0.000
URW          -6.030134 2 22.06027  6.922987     0.026
Stasis       -4.272037 2 18.54407  3.406793     0.150
StrictStasis -5.901974 1 15.13728  0.000000     0.824
Punc-1       -3.794462 4      Inf       Inf     0.000
OU           -3.994579 4      Inf       Inf     0.000
```

```
Total # hypotheses:  2 
1  2  

Comparing 6 models [n = 5, method = Joint]

                  logL K      AICc     dAICc Akaike.wt
GRW          -2.604279 3 35.208558 26.830897     0.000
URW          -2.610095 2 15.220190  6.842529     0.030
Stasis       -2.028179 2 14.056358  5.678697     0.054
StrictStasis -2.522164 1  8.377661  0.000000     0.916
Punc-1       -1.941583 4       Inf       Inf     0.000
OU           -1.768126 4       Inf       Inf     0.000
```

```
Total # hypotheses:  2 
1  2  

Comparing 6 models [n = 5, method = Joint]

                  logL K     AICc     dAICc Akaike.wt
GRW          -5.747880 3 41.49576 25.405329     0.000
URW          -6.874295 2 23.74859  7.658158     0.017
Stasis       -4.512877 2 19.02575  2.935321     0.184
StrictStasis -6.378549 1 16.09043  0.000000     0.799
Punc-1       -3.624104 4      Inf       Inf     0.000
OU           -4.307308 4      Inf       Inf     0.000
```

```
Total # hypotheses:  2 
1  2  

Comparing 6 models [n = 5, method = Joint]

                   logL K     AICc     dAICc Akaike.wt
GRW           -6.805559 3 43.61112 19.865914     0.000
URW           -9.504215 2 29.00843  5.263226     0.064
Stasis        -9.683583 2 29.36717  5.621962     0.053
StrictStasis -10.205935 1 23.74520  0.000000     0.883
Punc-1        -6.569014 4      Inf       Inf     0.000
OU            -6.106033 4      Inf       Inf     0.000
```

#### iii. PC wireframes

The final step in the analysis is to create the wireframe visualizations of the the principal components, to illustrate the shape changes over each PCA. The plots generated in this section are compiled in Figure 2 of the article.

```
#Extract the minimum and maximum of each of the four PCs analyzed
PCranges_neuro_neandertal <- data.frame(
  matrix(0,8,ncol(PCA_neuro_neandertal$PCS)+1))# 8 rows because we are getting minimum and maxium for each PC.

#Name the columns of the new dataframe
colnames(PCranges_neuro_neandertal) <- c("PC_limit",colnames(PCA_neuro_neandertal$PCS))

#In this loop, the minimum and maximum values for each PC are extracted from the PC data and scaled by the square root of the eigenvalues, as the first step in rotating this PC coordinate back into the original variables' coordinates
for(a in 1:4){
  
  minPCidx <- which(PCA_neuro_neandertal$PCS[,a]==min(PCA_neuro_neandertal$PCS[,a]))
  
  maxPCidx <- which(PCA_neuro_neandertal$PCS[,a]==max(PCA_neuro_neandertal$PCS[,a]))
  
  PCranges_neuro_neandertal[(a-1)*2+1,a+1] <- PCA_neuro_neandertal$PCS[minPCidx,a]*PCA_neuro_neandertal$eigenvalues[a,1]^0.5
  
  PCranges_neuro_neandertal[(a-1)*2+1,1] <- paste0("minPC",a)
  
  PCranges_neuro_neandertal[(a-1)*2+2,a+1] <- PCA_neuro_neandertal$PCS[maxPCidx,a]*PCA_neuro_neandertal$eigenvalues[a,1]^0.5
  
  PCranges_neuro_neandertal[(a-1)*2+2,1] <- paste0("maxPC",a)
  
}

#The PC coordinates are muliplied by the eigenvectors to complete the rotation to the origninal variable scales.

ranges_neuro_neandertal <- as.matrix(PCranges_neuro_neandertal[,2:ncol(PCranges_neuro_neandertal)])%*%t(PCA_neuro_neandertal$evectors)

means_neuro_neandertal <- apply(neuro_neandertal_nomiss[,2:ncol(neuro_neandertal_nomiss)],2,mean)

ranges_neuro_neandertal <- data.frame(t(apply(ranges_neuro_neandertal,1,function(x){x+means_neuro_neandertal})))

ranges_neuro_neandertal <- cbind(PCranges_neuro_neandertal$PC_limit,ranges_neuro_neandertal)

colnames(ranges_neuro_neandertal)[1] <- colnames(PCranges_neuro_neandertal)[1]


#The rotated coordinates are plotted for each PC.
#1. Setup camera parameters and colors for plots
camera_lat<-list(eye=list(x=0,y=2,z=0))
camera_front<-list(eye=list(x=2,y=0,z=0))
colors_wire<-c("#d16111","#227081") #color for max and min, respectively

#PC1 - lateral view plot
plot3d_df(ranges_neuro_neandertal[1:2,],plot_labels = FALSE,frame=wireframe_neuro[,3:4],color=colors_wire,plot.centroid = FALSE)%>%
layout(scene=list(xaxis=list(visible=F),yaxis=list(visible=F),zaxis=list(visible=F),camera=camera_front))
```

```
#PC1 - frontal view plot
plot3d_df(ranges_neuro_neandertal[1:2,],plot_labels = FALSE,frame=wireframe_neuro[,3:4],color=colors_wire,plot.centroid = FALSE)%>%
layout(scene=list(xaxis=list(visible=F),yaxis=list(visible=F),zaxis=list(visible=F),camera=camera_lat))
```

```
#PC2 - lateral view plot
plot3d_df(ranges_neuro_neandertal[3:4,],plot_labels = FALSE,frame=wireframe_neuro[,3:4],color=colors_wire,plot.centroid = FALSE)%>%
layout(scene=list(xaxis=list(visible=F),yaxis=list(visible=F),zaxis=list(visible=F),camera=camera_front))
```

```
#PC2 - frontal view plot
plot3d_df(ranges_neuro_neandertal[3:4,],plot_labels = FALSE,frame=wireframe_neuro[,3:4],color=colors_wire,plot.centroid = FALSE)%>%
layout(scene=list(xaxis=list(visible=F),yaxis=list(visible=F),zaxis=list(visible=F),camera=camera_lat))
```

```
#PC3 - lateral view plot
plot3d_df(ranges_neuro_neandertal[5:6,],plot_labels = FALSE,frame=wireframe_neuro[,3:4],color=colors_wire,plot.centroid = FALSE)%>%
layout(scene=list(xaxis=list(visible=F),yaxis=list(visible=F),zaxis=list(visible=F),camera=camera_front))
```

```
#PC3 - frontal view plot
plot3d_df(ranges_neuro_neandertal[5:6,],plot_labels = FALSE,frame=wireframe_neuro[,3:4],color=colors_wire,plot.centroid = FALSE)%>%
layout(scene=list(xaxis=list(visible=F),yaxis=list(visible=F),zaxis=list(visible=F),camera=camera_lat))
```

```
#PC4 - lateral view plot
plot3d_df(ranges_neuro_neandertal[7:8,],plot_labels = FALSE,frame=wireframe_neuro[,3:4],color=colors_wire,plot.centroid = FALSE)%>%
layout(scene=list(xaxis=list(visible=F),yaxis=list(visible=F),zaxis=list(visible=F),camera=camera_front))
```

```
#PC4 - frontal view plot
plot3d_df(ranges_neuro_neandertal[7:8,],plot_labels = FALSE,frame=wireframe_neuro[,3:4],color=colors_wire,plot.centroid = FALSE)%>%
layout(scene=list(xaxis=list(visible=F),yaxis=list(visible=F),zaxis=list(visible=F),camera=camera_lat))
```

### c. Facial analyses of the *Homo sapiens* lineage

This is the third set of analyses that are compiled with the other analyses to generate Tables 1, S4, and S5. The analyses are separated into Principal Components Analysis, evolutionary model testing, and finally the generation of the wireframes for the extreme values of the Principal Components to illustrate the morphological changes across each axis.

Before the analyses, specimens that are not part of the *H. sapiens* lineage are removed.

```
#create an index of which rows belong to the H. sapiens lineage
sapiens_lineage_index <- !face_data$SpeciesExpanded %in% c(
      "early H. neanderthalensis", "H. neanderthalensis"
      )

face_sapiens_nomiss <- face_nomiss|>
  filter( #Filter out species not on H. sapiens lineage
    sapiens_lineage_index
  )
```

#### i. Principal Components Analysis

PCA is used to explore the first four Principal Components through violin plots that are compiled in Figure 3 of the article.

The final violin plot presents the distribution of centroid size of specimens in each OTU, to explore specimen size.

```
#PCA is calculated using an imported custom function written by MH.
PCA_face_sapiens <- PCA(face_sapiens_nomiss[,2:ncol(face_nomiss)],face_sapiens_nomiss[,1])

#A dataframe is created to facilitate ploting via ggplot
PCA_face_sapiens_plotdf <- tibble(
  Specimen = face_sapiens_nomiss$Specimen,
  Species =  factor(face_data$SpeciesExpanded[sapiens_lineage_index],
                    levels = 
                       c("early Homo", "H. erectus",
                         "H. heidelbergensis s.l.", 
                         "early H. sapiens",
                         "Upper Paleolithic H. sapiens",
                         "H. sapiens")
  ),
  PC1 = PCA_face_sapiens$PCS[,1],
  PC2 = PCA_face_sapiens$PCS[,2],
  PC3 = PCA_face_sapiens$PCS[,3],
  PC4 = PCA_face_sapiens$PCS[,4],
  Centroid_size = face_gpa$Csize[sapiens_lineage_index]
)

#Plot PC1
ggplot(PCA_face_sapiens_plotdf, aes(y = PC1,x=Species))+
  geom_violin(aes(color=Species, fill = Species))+
  scale_colour_viridis_d(option="H")+
  geom_boxplot(width=0.1)+
  scale_fill_viridis_d(alpha=0.3,option="H")+
  labs(x = "PC1")
```

```
#Plot PC2
ggplot(PCA_face_sapiens_plotdf, aes(y = PC2,x=Species))+
  geom_violin(aes(color=Species, fill = Species))+
  scale_colour_viridis_d(option="H")+
  geom_boxplot(width=0.1)+
  scale_fill_viridis_d(alpha=0.3,option="H")+
  labs(x = "PC2")
```

```
#Plot PC3
ggplot(PCA_face_sapiens_plotdf, aes(y = PC3,x=Species))+
  geom_violin(aes(color=Species, fill = Species))+
  scale_colour_viridis_d(option="H")+
  geom_boxplot(width=0.1)+
  scale_fill_viridis_d(alpha=0.3,option="H")+
  labs(x = "PC3")
```

```
#Plot PC4
ggplot(PCA_face_sapiens_plotdf, aes(y = PC4,x=Species))+
  geom_violin(aes(color=Species, fill = Species))+
  scale_colour_viridis_d(option="H")+
  geom_boxplot(width=0.1)+
  scale_fill_viridis_d(alpha=0.3,option="H")+
  labs(x = "PC4")
```

```
#Plot centroid size
ggplot(PCA_face_sapiens_plotdf, aes(y = Centroid_size, x=Species))+
  geom_violin(aes(color=Species, fill = Species))+
  scale_colour_viridis_d(option="H")+
  geom_boxplot(width=0.1)+
  scale_fill_viridis_d(alpha=0.3,option="H")+
  labs(x = "Centroid Size")
```

The eigenvalues and percentage of variance explained for this analysis are reported here. This makes up part of Table S4.

```
PCA_face_sapiens$eigenvalues|>
  data.frame()|>
  slice(1:4)|>
  dplyr::rename(
    Eigenvalue = eigenvalues,
    `Variance %` = X..of.var,
    `Cumulative eigenvalue` = cum.evalues,
    `Cumulative Variance %` = cum...var
  )|>
  mutate(
    `Variance %` = round(`Variance %`,3),
    `Cumulative Variance %` = round(`Cumulative Variance %`,3)
  )|>
   kbl(caption = "Eigenvalues and variance explained for the facial analysis of the H. sapiens analysis")|>
  kable_styling(
    bootstrap_options = c("striped", "hover", "condensed"),
    full_width = F)
```

Eigenvalues and variance explained for the facial analysis of the H. sapiens analysis

|  | Eigenvalue | Variance % | Cumulative eigenvalue | Cumulative Variance % |
| --- | --- | --- | --- | --- |
| PC 1 | 0.0023441 | 22.122 | 0.0023441 | 22.122 |
| PC 2 | 0.0012312 | 11.619 | 0.0035753 | 33.741 |
| PC 3 | 0.0011311 | 10.674 | 0.0047063 | 44.415 |
| PC 4 | 0.0007030 | 6.634 | 0.0054093 | 51.049 |

#### ii. Evolutionary model testing

Next, we calculate the goodness of fit for different evolutionary models, following the method described in Hunt (2012). The analyses are done using package `EvoTS`, inside a wrapper function (`multiple_paleoTS`), which tests multiple models on the same dataset and generate a summary plot.

The text output from this analyses is presented in the article in Table 1 and Table S5, and the last plot generated is included in Figure 3 of the article.

```
#Create vector with species assignation
species_sapiens <- factor( face_data$SpeciesExpanded[sapiens_lineage_index]
)
                           
#load chronology data
chronology_sapiens <- taxa_chronology|>
  filter(
    !taxon %in% c(
      "early H. neanderthalensis", "H. neanderthalensis"
      )
  )

#This function prepares the data to be passed to PaleoTS
PTS_PCS_sapiens_face <- dists2PaleoTS(data.frame(PCA_face_sapiens_plotdf[,c(1,3:7)]),species_sapiens,chronology_sapiens)

#This wrapper function will test the 6 evolutionary models considered in the article against each of the PCs and the centroid size. The final figure output is used to compile Figure 3 in the article.
PTS_PCS_models_sapiens <- multiple_paleoTS(PTS_PCS_sapiens_face,models=6)
```

```
Total # hypotheses:  3 
1  2  3  

Comparing 6 models [n = 6, method = Joint]

                   logL K     AICc      dAICc Akaike.wt
GRW           -4.432408 3 26.86482  5.2486936     0.041
URW           -7.197961 2 22.39592  0.7797998     0.387
Stasis        -6.808061 2 21.61612  0.0000000     0.571
StrictStasis -16.371406 1 35.74281 14.1266902     0.000
Punc-1        -3.110836 4 54.22167 32.6055499     0.000
OU            -5.625006 4 59.25001 37.6338898     0.000
```

```
Total # hypotheses:  3 
1  2  3  

Comparing 6 models [n = 6, method = Joint]

                  logL K     AICc     dAICc Akaike.wt
GRW          -4.510591 3 27.02118 13.176204     0.001
URW          -5.458583 2 18.91717  5.072188     0.062
Stasis       -4.590183 2 17.18037  3.335386     0.149
StrictStasis -5.422490 1 13.84498  0.000000     0.788
Punc-1       -4.119220 4 56.23844 42.393461     0.000
OU           -2.696187 4 53.39237 39.547395     0.000
```

```
Total # hypotheses:  3 
1  2  3  

Comparing 6 models [n = 6, method = Joint]

                   logL K      AICc       dAICc Akaike.wt
GRW           -7.304073 3  32.60815   9.5179023     0.005
URW           -7.803298 2  23.60660   0.5163525     0.434
Stasis        -7.545122 2  23.09024   0.0000000     0.561
StrictStasis -66.169025 1 135.33805 112.2478061     0.000
Punc-1        -1.498696 4  50.99739  27.9071491     0.000
OU            -6.810413 4  61.62083  38.5305820     0.000
```

```
Total # hypotheses:  3 
1  2  3  

Comparing 6 models [n = 6, method = Joint]

                   logL K     AICc       dAICc Akaike.wt
GRW           -6.963567 3 31.92713  9.92584932     0.003
URW           -7.000642 2 22.00128  0.00000000     0.461
Stasis        -7.045228 2 22.09046  0.08917273     0.441
StrictStasis -11.073748 1 25.14750  3.14621229     0.096
Punc-1        -4.479903 4 56.95981 34.95852296     0.000
OU            -5.807727 4 59.61545 37.61417031     0.000
```

```
Total # hypotheses:  3 
1  2  3  

Comparing 6 models [n = 6, method = Joint]

                   logL K      AICc      dAICc Akaike.wt
GRW          -12.503944 3  43.00789 10.0533279     0.003
URW          -12.562212 2  33.12442  0.1698642     0.477
Stasis       -12.477280 2  32.95456  0.0000000     0.519
StrictStasis -52.586388 1 108.17278 75.2182170     0.000
Punc-1        -7.379996 4  62.75999 29.8054319     0.000
OU           -11.297935 4  70.59587 37.6413107     0.000
```

#### iii. PC wireframes

The final step in the analysis is to create the wireframe visualizations of the the principal components, to illustrate the shape changes over each PCA. The plots generated in this section are compiled in Figure 3 of the article.

```
#Extract the minimum and maximum of each of the four PCs analyzed
PCranges_face_sapiens <- data.frame(
  matrix(0,8,ncol(PCA_face_sapiens$PCS)+1))# 8 rows because we are getting minimum and maxium for each PC.

#Name the columns as of the new dataframe
colnames(PCranges_face_sapiens) <- c("PC_limit",colnames(PCA_face_sapiens$PCS))

#In this loop, the minimum and maximum values for each PC are extracted from the PC data and scaled by the square root of the eigenvalues, as the first step in rotating this PC coordinate back into the original variables' coordinates
for(a in 1:4){
  
  minPCidx <- which(PCA_face_sapiens$PCS[,a]==min(PCA_face_sapiens$PCS[,a]))
  
  maxPCidx <- which(PCA_face_sapiens$PCS[,a]==max(PCA_face_sapiens$PCS[,a]))
  
  PCranges_face_sapiens[(a-1)*2+1,a+1] <- PCA_face_sapiens$PCS[minPCidx,a]*PCA_face_sapiens$eigenvalues[a,1]^0.5
  
  PCranges_face_sapiens[(a-1)*2+1,1] <- paste0("minPC",a)
  
  PCranges_face_sapiens[(a-1)*2+2,a+1] <- PCA_face_sapiens$PCS[maxPCidx,a]*PCA_face_sapiens$eigenvalues[a,1]^0.5
  
  PCranges_face_sapiens[(a-1)*2+2,1] <- paste0("maxPC",a)
  
}

#The PC coordinates are muliplied by the eigenvectors to complete the rotation to the origninal variable scales.

ranges_face_sapiens <- as.matrix(PCranges_face_sapiens[,2:ncol(PCranges_face_sapiens)])%*%t(PCA_face_sapiens$evectors)

means_face_sapiens <- apply(face_sapiens_nomiss[,2:ncol(face_sapiens_nomiss)],2,mean)

ranges_face_sapiens <- data.frame(t(apply(ranges_face_sapiens,1,function(x){x+means_face_sapiens})))

ranges_face_sapiens <- cbind(PCranges_face_sapiens$PC_limit,ranges_face_sapiens)

colnames(ranges_face_sapiens)[1] <- colnames(PCranges_face_sapiens)[1]

#next step the wireframe matrix is created this is a list table that ids which landmarks connect to which in the wireframe plot.
#1. Get the variable names
face_variables_name <- colnames(face_sapiens_nomiss)[-1]
face_variables_name <- face_variables_name[seq(1,length(face_variables_name),by=3)]|>
  str_replace("_X","")

#2.create the pairs of variables to be connected
wireframe_face <- tribble(
  ~start, ~end,
  "Distal M3 R",    "Canine-Premolar contact R",
  "Canine-Premolar contact R", "Prosthion",
  "Distal M3 L",    "Canine-Premolar contact L",
  "Canine-Premolar contact L",  "Prosthion",
  "Nasospinale",    "Prosthion",
  "Nasion", "Mid-orbit torus superior R",
  "Nasion", "Dacryon R",
  "Nasion", "Alare R",
  "Nasion", "Alare L",
  "Dacryon R",  "Zygoorbitale R",
  "Zygoorbitale R", "Frontomalare Orb R",
  "Mid-orbit torus superior R", "Frontomalare Orb R",
  "Nasospinale",    "Alare R",
  "Nasion", "Mid-orbit torus superior L",
  "Nasion", "Dacryon L",
  "Dacryon L",  "Zygoorbitale L",
  "Zygoorbitale L", "Frontomalare Orb L",
  "Mid-orbit torus superior L", "Frontomalare Orb L",
  "Nasospinale",    "Alare L"

)

#3. Match them with the variable number to pass as a numerical index to the plot
wireframe_face$start_idx<-NA
wireframe_face$end_idx<-NA
for(a in 1:nrow(wireframe_face)){
  wireframe_face$start_idx[a]<-which(face_variables_name == wireframe_face$start[a])
  wireframe_face$end_idx[a]<-which(face_variables_name  == wireframe_face$end[a])
}

#The rotated coordinates are ploted for each PC.
#1. Setup camera parameters and colors for plots
camera_lat<-list(eye=list(x=0,y=0,z=2))
camera_front<-list(eye=list(x=2,y=0,z=0))
colors_wire<-c("#d16111","#227081") #color for max and min, respectively

#PC1 - lateral view plot
plot3d_df(ranges_face_sapiens[1:2,],plot_labels = FALSE,frame=wireframe_face[,3:4],color=colors_wire,plot.centroid = FALSE)%>%
layout(scene=list(xaxis=list(visible=F),yaxis=list(visible=F),zaxis=list(visible=F),camera=camera_front))
```

```
#PC1 - frontal view plot
plot3d_df(ranges_face_sapiens[1:2,],plot_labels = FALSE,frame=wireframe_face[,3:4],color=colors_wire,plot.centroid = FALSE)%>%
layout(scene=list(xaxis=list(visible=F),yaxis=list(visible=F),zaxis=list(visible=F),camera=camera_lat))
```

```
#PC2 - lateral view plot
plot3d_df(ranges_face_sapiens[3:4,],plot_labels = FALSE,frame=wireframe_face[,3:4],color=colors_wire,plot.centroid = FALSE)%>%
layout(scene=list(xaxis=list(visible=F),yaxis=list(visible=F),zaxis=list(visible=F),camera=camera_front))
```

```
#PC2 - frontal view plot
plot3d_df(ranges_face_sapiens[3:4,],plot_labels = FALSE,frame=wireframe_face[,3:4],color=colors_wire,plot.centroid = FALSE)%>%
layout(scene=list(xaxis=list(visible=F),yaxis=list(visible=F),zaxis=list(visible=F),camera=camera_lat))
```

```
#PC3 - lateral view plot
plot3d_df(ranges_face_sapiens[5:6,],plot_labels = FALSE,frame=wireframe_face[,3:4],color=colors_wire,plot.centroid = FALSE)%>%
layout(scene=list(xaxis=list(visible=F),yaxis=list(visible=F),zaxis=list(visible=F),camera=camera_front))
```

```
#PC3 - frontal view plot
plot3d_df(ranges_face_sapiens[5:6,],plot_labels = FALSE,frame=wireframe_face[,3:4],color=colors_wire,plot.centroid = FALSE)%>%
layout(scene=list(xaxis=list(visible=F),yaxis=list(visible=F),zaxis=list(visible=F),camera=camera_lat))
```

```
#PC4 - lateral view plot
plot3d_df(ranges_face_sapiens[7:8,],plot_labels = FALSE,frame=wireframe_face[,3:4],color=colors_wire,plot.centroid = FALSE)%>%
layout(scene=list(xaxis=list(visible=F),yaxis=list(visible=F),zaxis=list(visible=F),camera=camera_front))
```

```
#PC4 - frontal view plot
plot3d_df(ranges_face_sapiens[7:8,],plot_labels = FALSE,frame=wireframe_face[,3:4],color=colors_wire,plot.centroid = FALSE)%>%
layout(scene=list(xaxis=list(visible=F),yaxis=list(visible=F),zaxis=list(visible=F),camera=camera_lat))
```

### d. Facial analyses of the *Homo neanderthalensis* lineage

This fourth block of analyses completes the set of results that generate Figure 4 and are compiled with the other analyses to generate Tables 1, S4, and S5.

Before the analyses, specimens that are not part of the *H. neanderthalensis* lineage are removed.

```
#create an index of which rows belong to the H. sapiens lineage
neandertal_lineage_index <- !face_data$SpeciesExpanded %in% c(
      "early H. sapiens", "Upper Paleolithic H. sapiens","H. sapiens"
      )

face_neandertal_nomiss <- face_nomiss|>
  filter( #Filter out species not on H. neanderthalensis lineage
    neandertal_lineage_index
  )
```

#### i. Principal Components Analysis

PCA is used to explore the first four Principal Components through violin plots that are compiled in Figure 4 in the article.

The final violin plot presents the distribution of centroid size of specimens in each OTU, to explore specimen size.

```
#PCA is calculated using an imported custom function written by MH.
PCA_face_neandertal <- PCA(face_neandertal_nomiss[,2:ncol(face_nomiss)],face_neandertal_nomiss[,1])

#A dataframe is created to facilitate ploting via ggplot
PCA_face_neandertal_plotdf <- tibble(
  Specimen = face_neandertal_nomiss$Specimen,
  Species =  factor(face_data$SpeciesExpanded[neandertal_lineage_index],
                    levels = 
                       c("early Homo", "H. erectus",
                         "H. heidelbergensis s.l.", 
                         "early H. neanderthalensis",
                         "H. neanderthalensis")
  ),
  PC1 = PCA_face_neandertal$PCS[,1],
  PC2 = PCA_face_neandertal$PCS[,2],
  PC3 = PCA_face_neandertal$PCS[,3],
  PC4 = PCA_face_neandertal$PCS[,4],
  Centroid_size = face_gpa$Csize[neandertal_lineage_index]
)

#Plot PC1
ggplot(PCA_face_neandertal_plotdf, aes(y = PC1,x=Species))+
  geom_violin(aes(color=Species, fill = Species))+
  scale_colour_viridis_d(option="H")+
  geom_boxplot(width=0.1)+
  scale_fill_viridis_d(alpha=0.3,option="H")+
  labs(x = "PC1")
```

```
#Plot PC2
ggplot(PCA_face_neandertal_plotdf, aes(y = PC2,x=Species))+
  geom_violin(aes(color=Species, fill = Species))+
  scale_colour_viridis_d(option="H")+
  geom_boxplot(width=0.1)+
  scale_fill_viridis_d(alpha=0.3,option="H")+
  labs(x = "PC2")
```

```
#Plot PC3
ggplot(PCA_face_neandertal_plotdf, aes(y = PC3,x=Species))+
  geom_violin(aes(color=Species, fill = Species))+
  scale_colour_viridis_d(option="H")+
  geom_boxplot(width=0.1)+
  scale_fill_viridis_d(alpha=0.3,option="H")+
  labs(x = "PC3")
```

```
#Plot PC4
ggplot(PCA_face_neandertal_plotdf, aes(y = PC4,x=Species))+
  geom_violin(aes(color=Species, fill = Species))+
  scale_colour_viridis_d(option="H")+
  geom_boxplot(width=0.1)+
  scale_fill_viridis_d(alpha=0.3,option="H")+
  labs(x = "PC4")
```

```
#Plot centroid size
ggplot(PCA_face_neandertal_plotdf, aes(y = Centroid_size, x=Species))+
  geom_violin(aes(color=Species, fill = Species))+
  scale_colour_viridis_d(option="H")+
  geom_boxplot(width=0.1)+
  scale_fill_viridis_d(alpha=0.3,option="H")+
  labs(x = "Centroid Size")
```

The eigenvalues and percentage of variance explained for this analysis are reported here. This makes up part of Table S4.

```
PCA_face_sapiens$eigenvalues|>
  data.frame()|>
  slice(1:4)|>
  dplyr::rename(
    Eigenvalue = eigenvalues,
    `Variance %` = X..of.var,
    `Cumulative eigenvalue` = cum.evalues,
    `Cumulative Variance %` = cum...var
  )|>
  mutate(
    `Variance %` = round(`Variance %`,3),
    `Cumulative Variance %` = round(`Cumulative Variance %`,3)
  )|>
   kbl(caption = "Eigenvalues and variance explained for the facial analysis of the H. sapiens analysis")|>
  kable_styling(
    bootstrap_options = c("striped", "hover", "condensed"),
    full_width = F)
```

Eigenvalues and variance explained for the facial analysis of the H. sapiens analysis

|  | Eigenvalue | Variance % | Cumulative eigenvalue | Cumulative Variance % |
| --- | --- | --- | --- | --- |
| PC 1 | 0.0023441 | 22.122 | 0.0023441 | 22.122 |
| PC 2 | 0.0012312 | 11.619 | 0.0035753 | 33.741 |
| PC 3 | 0.0011311 | 10.674 | 0.0047063 | 44.415 |
| PC 4 | 0.0007030 | 6.634 | 0.0054093 | 51.049 |

#### ii. Evolutionary model testing

Next, we calculate the goodness of fit for different evolutionary models, following the method described in Hunt (2012). The analyses are done using package `EvoTS`, inside a wrapper function (`multiple_paleoTS`), which tests multiple models on the same dataset and generate a summary plot.

The text output from these analyses is presented in the article in Table 1 and Table S5, and the last plot generated is included in Figure 4 of the article.

```
#Create vector with species assignation
species_neandertal <- factor( face_data$SpeciesExpanded[neandertal_lineage_index]
)
                           
#load chronology data
chronology_neandertal <- taxa_chronology|>
  filter(
    !taxon %in% c(
      "early H. sapiens", "Upper Paleolithic H. sapiens","H. sapiens"
      )
  )

#This function prepares the data to be passed to PaleoTS
PTS_PCS_neandertal <- dists2PaleoTS(data.frame(PCA_face_neandertal_plotdf[,c(1,3:7)]),species_neandertal,chronology_neandertal)

#This wrapper function will  test the 6 evolutionary models considered in the article against each of the PCs and the centroid size. The final figure output is used to compile Figure 1 in the article.
PTS_PCS_models_neandertal <- multiple_paleoTS(PTS_PCS_neandertal,models=6)
```

```
Total # hypotheses:  2 
1  2  

Comparing 6 models [n = 5, method = Joint]

                  logL K     AICc     dAICc Akaike.wt
GRW          -2.393729 3 34.78746 26.283298     0.000
URW          -2.615183 2 15.23037  6.726206     0.032
Stasis       -2.585423 2 15.17085  6.666685     0.033
StrictStasis -2.585413 1  8.50416  0.000000     0.934
Punc-1       -2.401739 4      Inf       Inf     0.000
OU           -2.364121 4      Inf       Inf     0.000
```

```
Warning in compareModels(grw, urw, sta, strsta, pun2, ou): Optimization for the following model(s) did not converge: OU
These model fit(s) should not be considered reliable.
```

```
Total # hypotheses:  2 
1  2  

Comparing 6 models [n = 5, method = Joint]

                     logL K     AICc     dAICc Akaike.wt
GRW            1.17763133 3 27.64474 10.088663     0.006
URW           -3.77803710 2 17.55607  0.000000     0.951
Stasis        -6.88401131 2 23.76802  6.211948     0.043
StrictStasis -23.63602708 1 50.60539 33.049313     0.000
Punc-1         0.01665848 4      Inf       Inf     0.000
OU             1.24310603 4      Inf       Inf     0.000
```

```
Warning in compareModels(grw, urw, sta, strsta, pun2, ou): Optimization for the following model(s) did not converge: OU
These model fit(s) should not be considered reliable.
```

```
Total # hypotheses:  2 
1  2  

Comparing 6 models [n = 5, method = Joint]

                  logL K     AICc     dAICc Akaike.wt
GRW          -4.393047 3 38.78609 26.584258     0.000
URW          -4.454267 2 18.90853  6.706697     0.032
Stasis       -3.947095 2 17.89419  5.692352     0.053
StrictStasis -4.434252 1 12.20184  0.000000     0.915
Punc-1       -3.038820 4      Inf       Inf     0.000
OU           -3.839638 4      Inf       Inf     0.000
```

```
Total # hypotheses:  2 
1  2  

Comparing 6 models [n = 5, method = Joint]

                  logL K     AICc      dAICc Akaike.wt
GRW          -3.464950 3 36.92990 16.3263122     0.000
URW          -5.701441 2 21.40288  0.7992943     0.267
Stasis       -5.301794 2 20.60359  0.0000000     0.398
StrictStasis -8.804773 1 20.94288  0.3392920     0.336
Punc-1       -4.308985 4      Inf        Inf     0.000
OU           -2.560997 4      Inf        Inf     0.000
```

```
Total # hypotheses:  2 
1  2  

Comparing 6 models [n = 5, method = Joint]

                  logL K     AICc     dAICc Akaike.wt
GRW          -6.054239 3 42.10848 25.582857     0.000
URW          -6.606905 2 23.21381  6.688189     0.033
Stasis       -6.596146 2 23.19229  6.666671     0.033
StrictStasis -6.596143 1 16.52562  0.000000     0.934
Punc-1       -5.741705 4      Inf       Inf     0.000
OU           -5.399597 4      Inf       Inf     0.000
```

#### iii. PC wireframes

The final step in the analysis is to create the wireframe visualizations of the the principal components, to illustrate the shape changes over each PCA. The plots generated in this section are compiled in Figure 1 of the article.

```
#Extract the minimum and maximum of each of the four PCs analyzed
PCranges_face_neandertal <- data.frame(
  matrix(0,8,ncol(PCA_face_neandertal$PCS)+1))# 8 rows because we are getting minimum and maxium for each PC.

#Name the columns as of the new dataframe
colnames(PCranges_face_neandertal) <- c("PC_limit",colnames(PCA_face_neandertal$PCS))

#In this loop, the minimum and maximum values for each PC are extracted from the PC data and scaled by the square root of the eigenvalues, as the first step in rotating this PC coordinate back into the original variables' coordinates
for(a in 1:4){
  
  minPCidx <- which(PCA_face_neandertal$PCS[,a]==min(PCA_face_neandertal$PCS[,a]))
  
  maxPCidx <- which(PCA_face_neandertal$PCS[,a]==max(PCA_face_neandertal$PCS[,a]))
  
  PCranges_face_neandertal[(a-1)*2+1,a+1] <- PCA_face_neandertal$PCS[minPCidx,a]*PCA_face_neandertal$eigenvalues[a,1]^0.5
  
  PCranges_face_neandertal[(a-1)*2+1,1] <- paste0("minPC",a)
  
  PCranges_face_neandertal[(a-1)*2+2,a+1] <- PCA_face_neandertal$PCS[maxPCidx,a]*PCA_face_neandertal$eigenvalues[a,1]^0.5
  
  PCranges_face_neandertal[(a-1)*2+2,1] <- paste0("maxPC",a)
  
}

#The PC coordinates are muliplied by the eigenvectors to complete the rotation to the origninal variable scales.

ranges_face_neandertal <- as.matrix(PCranges_face_neandertal[,2:ncol(PCranges_face_neandertal)])%*%t(PCA_face_neandertal$evectors)

means_face_neandertal <- apply(face_neandertal_nomiss[,2:ncol(face_neandertal_nomiss)],2,mean)

ranges_face_neandertal <- data.frame(t(apply(ranges_face_neandertal,1,function(x){x+means_face_neandertal})))

ranges_face_neandertal <- cbind(PCranges_face_neandertal$PC_limit,ranges_face_neandertal)

colnames(ranges_face_neandertal)[1] <- colnames(PCranges_face_neandertal)[1]


#The rotated coordinates are plotted for each PC.
#1. Setup camera parameters and colors for plots
camera_lat<-list(eye=list(x=0,y=0,z=2))
camera_front<-list(eye=list(x=2,y=0,z=0))
colors_wire<-c("#d16111","#227081") #color for max and min, respectively

#PC1 - lateral view plot
plot3d_df(ranges_face_neandertal[1:2,],plot_labels = FALSE,frame=wireframe_face[,3:4],color=colors_wire,plot.centroid = FALSE)%>%
layout(scene=list(xaxis=list(visible=F),yaxis=list(visible=F),zaxis=list(visible=F),camera=camera_front))
```

```
#PC1 - frontal view plot
plot3d_df(ranges_face_neandertal[1:2,],plot_labels = FALSE,frame=wireframe_face[,3:4],color=colors_wire,plot.centroid = FALSE)%>%
layout(scene=list(xaxis=list(visible=F),yaxis=list(visible=F),zaxis=list(visible=F),camera=camera_lat))
```

```
#PC2 - lateral view plot
plot3d_df(ranges_face_neandertal[3:4,],plot_labels = FALSE,frame=wireframe_face[,3:4],color=colors_wire,plot.centroid = FALSE)%>%
layout(scene=list(xaxis=list(visible=F),yaxis=list(visible=F),zaxis=list(visible=F),camera=camera_front))
```

```
#PC2 - frontal view plot
plot3d_df(ranges_face_neandertal[3:4,],plot_labels = FALSE,frame=wireframe_face[,3:4],color=colors_wire,plot.centroid = FALSE)%>%
layout(scene=list(xaxis=list(visible=F),yaxis=list(visible=F),zaxis=list(visible=F),camera=camera_lat))
```

```
#PC3 - lateral view plot
plot3d_df(ranges_face_sapiens[5:6,],plot_labels = FALSE,frame=wireframe_face[,3:4],color=colors_wire,plot.centroid = FALSE)%>%
layout(scene=list(xaxis=list(visible=F),yaxis=list(visible=F),zaxis=list(visible=F),camera=camera_front))
```

```
#PC3 - frontal view plot
plot3d_df(ranges_face_neandertal[5:6,],plot_labels = FALSE,frame=wireframe_face[,3:4],color=colors_wire,plot.centroid = FALSE)%>%
layout(scene=list(xaxis=list(visible=F),yaxis=list(visible=F),zaxis=list(visible=F),camera=camera_lat))
```

```
#PC4 - lateral view plot
plot3d_df(ranges_face_sapiens[7:8,],plot_labels = FALSE,frame=wireframe_face[,3:4],color=colors_wire,plot.centroid = FALSE)%>%
layout(scene=list(xaxis=list(visible=F),yaxis=list(visible=F),zaxis=list(visible=F),camera=camera_front))
```

```
#PC4 - frontal view plot
plot3d_df(ranges_face_neandertal[7:8,],plot_labels = FALSE,frame=wireframe_face[,3:4],color=colors_wire,plot.centroid = FALSE)%>%
layout(scene=list(xaxis=list(visible=F),yaxis=list(visible=F),zaxis=list(visible=F),camera=camera_lat))
```

## 2. Supplementary analyses

The supplementary analyses presented in the article are replicated here. They represent different combinations of specimens into OTUs to explore the five alternative scenarios detailed in the article. All analyses follow the same structure of the main article analyses (Section 1 of this document), and their results are compiled in Tables S7-S11.

### 2a. Lineages starting with *Homo erectus*

Given the debate about the phylogenetic position of early *Homo* (*H. habilis* and *H. rudolphensis*) in the *Homo* lineage, and the fact that we had to group fossils assigned to these two species into an early *Homo* OTU, this section presents the eresults for lineages that do not include this OTU.

#### i. Neurocranial analyses of the *Homo sapiens* lineage

All analyses are presented inside a single code chunk. PC plots and Evolutionary model summary plots are generated here, but in the article only the summary table for evolutionary models is presented in Table S7.

```
#1. Remove early Homo from dataset
sapiens_lineage_index_2ai <- !neurocranium_data$SpeciesExpanded %in% c(
      "early Homo",
      "early H. neanderthalensis",
      "H. neanderthalensis"
      )

neuro_sapiens_nomiss_2ai <- neuro_nomiss|>
  filter( #Filter out species not on H. sapiens lineage
    sapiens_lineage_index_2ai
  )

#2. Calculate PCA
PCA_neuro_sapiens_2ai <- PCA(neuro_sapiens_nomiss_2ai[,2:ncol(neuro_nomiss)],neuro_sapiens_nomiss_2ai[,1])

#A dataframe is created to facilitate ploting via ggplot
PCA_neuro_sapiens_plotdf_2ai <- tibble(
  Specimen = neuro_sapiens_nomiss_2ai$Specimen,
  Species =  factor(neurocranium_data$SpeciesExpanded[sapiens_lineage_index_2ai],
                    levels = 
                       c("H. erectus",
                         "H. heidelbergensis s.l.", 
                         "early H. sapiens",
                         "Upper Paleolithic H. sapiens",
                         "H. sapiens")
  ),
  PC1 = PCA_neuro_sapiens_2ai$PCS[,1],
  PC2 = PCA_neuro_sapiens_2ai$PCS[,2],
  PC3 = PCA_neuro_sapiens_2ai$PCS[,3],
  PC4 = PCA_neuro_sapiens_2ai$PCS[,4],
  Centroid_size = neuro_gpa$Csize[sapiens_lineage_index_2ai]
)

#Plot PC1
ggplot(PCA_neuro_sapiens_plotdf_2ai, aes(y = PC1,x=Species))+
  geom_violin(aes(color=Species, fill = Species))+
  scale_colour_viridis_d(option="H")+
  geom_boxplot(width=0.1)+
  scale_fill_viridis_d(alpha=0.3,option="H")+
  labs(x = "PC1")
```

```
#Plot PC2
ggplot(PCA_neuro_sapiens_plotdf_2ai, aes(y = PC2,x=Species))+
  geom_violin(aes(color=Species, fill = Species))+
  scale_colour_viridis_d(option="H")+
  geom_boxplot(width=0.1)+
  scale_fill_viridis_d(alpha=0.3,option="H")+
  labs(x = "PC2")
```

```
#Plot PC3
ggplot(PCA_neuro_sapiens_plotdf_2ai, aes(y = PC3,x=Species))+
  geom_violin(aes(color=Species, fill = Species))+
  scale_colour_viridis_d(option="H")+
  geom_boxplot(width=0.1)+
  scale_fill_viridis_d(alpha=0.3,option="H")+
  labs(x = "PC3")
```

```
#Plot PC4
ggplot(PCA_neuro_sapiens_plotdf_2ai, aes(y = PC4,x=Species))+
  geom_violin(aes(color=Species, fill = Species))+
  scale_colour_viridis_d(option="H")+
  geom_boxplot(width=0.1)+
  scale_fill_viridis_d(alpha=0.3,option="H")+
  labs(x = "PC4")
```

```
#Plot centroid size
ggplot(PCA_neuro_sapiens_plotdf_2ai, aes(y = Centroid_size, x=Species))+
  geom_violin(aes(color=Species, fill = Species))+
  scale_colour_viridis_d(option="H")+
  geom_boxplot(width=0.1)+
  scale_fill_viridis_d(alpha=0.3,option="H")+
  labs(x = "Centroid Size")
```

```
#3.Evolutionary models

#Create vector with species assignation
species_sapiens_2ai <- factor( neurocranium_data$SpeciesExpanded[sapiens_lineage_index_2ai]
)
                           
#load chronology data
chronology_sapiens_2ai <- taxa_chronology|>
  filter(
    !taxon %in% c(
      "early Homo",
      "early H. neanderthalensis", 
      "H. neanderthalensis"
      )
  )

#This function prepares the data to be passed to PaleoTS
PTS_PCS_sapiens_2ai <- dists2PaleoTS(data.frame(PCA_neuro_sapiens_plotdf_2ai[,c(1,3:7)]),species_sapiens_2ai,chronology_sapiens_2ai)

#This wrapper function will run test the 6 evolutionary models considered in the article against each of the PCs and the centroid size.
PTS_PCS_models_sapiens_2ai <- multiple_paleoTS(PTS_PCS_sapiens_2ai,models=6)
```

```
Total # hypotheses:  2 
1  2  

Comparing 6 models [n = 5, method = Joint]

                   logL K      AICc      dAICc Akaike.wt
GRW           -2.645860 3  35.29172  18.194549     0.000
URW           -3.548585 2  17.09717   0.000000     0.969
Stasis        -7.008926 2  24.01785   6.920681     0.030
StrictStasis -91.855386 1 187.04411 169.946935     0.000
Punc-1        -1.532557 4       Inf        Inf     0.000
OU            -2.647496 4       Inf        Inf     0.000
```

```
Warning in compareModels(grw, urw, sta, strsta, pun2, ou): Optimization for the following model(s) did not converge: OU
These model fit(s) should not be considered reliable.
```

```
Total # hypotheses:  2 
1  2  

Comparing 6 models [n = 5, method = Joint]

                   logL K     AICc     dAICc Akaike.wt
GRW          -3.0721584 3 36.14432 19.362903     0.000
URW          -3.3907070 2 16.78141  0.000000     0.614
Stasis       -4.8567313 2 19.71346  2.932049     0.142
StrictStasis -7.6440442 1 18.62142  1.840008     0.245
Punc-1       -4.7848653 4      Inf       Inf     0.000
OU           -0.7839199 4      Inf       Inf     0.000
```

```
Total # hypotheses:  2 
1  2  

Comparing 6 models [n = 5, method = Joint]

                  logL K     AICc     dAICc Akaike.wt
GRW          -3.051098 3 36.10220 21.455295     0.000
URW          -4.492048 2 18.98410  4.337195     0.085
Stasis       -2.323450 2 14.64690  0.000000     0.742
StrictStasis -7.108826 1 17.55099  2.904086     0.174
Punc-1       -1.017176 4      Inf       Inf     0.000
OU           -2.067198 4      Inf       Inf     0.000
```

```
Total # hypotheses:  2 
1  2  

Comparing 6 models [n = 5, method = Joint]

                   logL K      AICc     dAICc Akaike.wt
GRW          -1.9798596 3 33.959719 26.739396     0.000
URW          -1.9954863 2 13.990973  6.770649     0.031
Stasis       -1.5767172 2 13.153434  5.933111     0.047
StrictStasis -1.9434951 1  7.220324  0.000000     0.921
Punc-1       -0.9198867 4       Inf       Inf     0.000
OU           -1.4663532 4       Inf       Inf     0.000
```

```
Total # hypotheses:  2 
1  2  

Comparing 6 models [n = 5, method = Joint]

                   logL K     AICc     dAICc Akaike.wt
GRW          -10.390225 3 50.78045 23.762482     0.000
URW          -10.395697 2 30.79139  3.773427     0.132
Stasis        -8.508983 2 27.01797  0.000000     0.868
StrictStasis -19.578523 1 42.49038 15.472413     0.000
Punc-1        -8.197793 4      Inf       Inf     0.000
OU            -7.348953 4      Inf       Inf     0.000
```

#### ii. Neurocranial analysis of the *Homo neanderthalensis* lineage

This follows the same structure as the previous analysis.

```
#1. Remove early Homo from dataset
neandertal_lineage_index_2aii <- !neurocranium_data$SpeciesExpanded %in% c(
  "early Homo",
  "early H. sapiens",
  "Upper Paleolithic H. sapiens",
  "H. sapiens"
      )

neuro_neandertal_nomiss_2aii <- neuro_nomiss|>
  filter( #Filter out species not on H. neanderthalensis lineage
    neandertal_lineage_index_2aii
  )

#2. Calculate PCA
PCA_neuro_neandertal_2aii <- PCA(neuro_neandertal_nomiss_2aii[,2:ncol(neuro_nomiss)],neuro_neandertal_nomiss_2aii[,1])

#A dataframe is created to facilitate ploting via ggplot
PCA_neuro_neandertal_plotdf_2aii <- tibble(
  Specimen = neuro_neandertal_nomiss_2aii$Specimen,
  Species =  factor(neurocranium_data$SpeciesExpanded[neandertal_lineage_index_2aii],
                    levels = 
                       c("H. erectus",
                         "H. heidelbergensis s.l.", 
                         "early H. neanderthalensis",
                         "H. neanderthalensis")
  ),
  PC1 = PCA_neuro_neandertal_2aii$PCS[,1],
  PC2 = PCA_neuro_neandertal_2aii$PCS[,2],
  PC3 = PCA_neuro_neandertal_2aii$PCS[,3],
  PC4 = PCA_neuro_neandertal_2aii$PCS[,4],
  Centroid_size = neuro_gpa$Csize[neandertal_lineage_index_2aii]
)

#Plot  PC1
ggplot(PCA_neuro_neandertal_plotdf_2aii, aes(y = PC1,x=Species))+
  geom_violin(aes(color=Species, fill = Species))+
  scale_colour_viridis_d(option="H")+
  geom_boxplot(width=0.1)+
  scale_fill_viridis_d(alpha=0.3,option="H")+
  labs(x = "PC1")
```

```
#Plot  PC2
ggplot(PCA_neuro_neandertal_plotdf_2aii, aes(y = PC2,x=Species))+
  geom_violin(aes(color=Species, fill = Species))+
  scale_colour_viridis_d(option="H")+
  geom_boxplot(width=0.1)+
  scale_fill_viridis_d(alpha=0.3,option="H")+
  labs(x = "PC2")
```

```
#Plot  PC3
ggplot(PCA_neuro_neandertal_plotdf_2aii, aes(y = PC3,x=Species))+
  geom_violin(aes(color=Species, fill = Species))+
  scale_colour_viridis_d(option="H")+
  geom_boxplot(width=0.1)+
  scale_fill_viridis_d(alpha=0.3,option="H")+
  labs(x = "PC3")
```

```
#Plot PC4
ggplot(PCA_neuro_neandertal_plotdf_2aii, aes(y = PC4,x=Species))+
  geom_violin(aes(color=Species, fill = Species))+
  scale_colour_viridis_d(option="H")+
  geom_boxplot(width=0.1)+
  scale_fill_viridis_d(alpha=0.3,option="H")+
  labs(x = "PC4")
```

```
#Plot centroid size
ggplot(PCA_neuro_neandertal_plotdf_2aii, aes(y = Centroid_size, x=Species))+
  geom_violin(aes(color=Species, fill = Species))+
  scale_colour_viridis_d(option="H")+
  geom_boxplot(width=0.1)+
  scale_fill_viridis_d(alpha=0.3,option="H")+
  labs(x = "Centroid Size")
```

```
#3 Evolutionary models

#Create vector with species assignation
species_neandertal_2aii <- factor( neurocranium_data$SpeciesExpanded[neandertal_lineage_index_2aii]
)
                           
#load chronology data
chronology_neandertal_2aii <- taxa_chronology|>
  filter(
    !taxon %in% c(
      "early Homo",
      "early H. sapiens",
      "Upper Paleolithic H. sapiens",
      "H. sapiens"
      )
  )

#This function prepares the data to be passed to PaleoTS
PTS_PCS_neandertal_2aii <- dists2PaleoTS(data.frame(PCA_neuro_neandertal_plotdf_2aii[,c(1,3:7)]),species_neandertal_2aii,chronology_neandertal_2aii)

#This wrapper function will test the 6 evolutionary models considered in the article against each of the PCs and the centroid size. 
PTS_PCS_models_neandertal_2aii <- multiple_paleoTS(PTS_PCS_neandertal_2aii,models=6)
```

```
Total # hypotheses:  1 
1  

Comparing 6 models [n = 4, method = Joint]

                   logL K      AICc     dAICc Akaike.wt
GRW            1.311474 3       Inf       Inf     0.000
URW           -2.750760 2  21.50152 56.139219     0.000
Stasis        -4.856809 2  25.71362 60.351318     0.000
StrictStasis -29.350856 1  62.70171 97.339412     0.000
Punc-1        -3.079986 4 -25.84003  8.797672     0.012
OU             1.318850 4 -34.63770  0.000000     0.988
```

```
Warning in compareModels(grw, urw, sta, strsta, pun2, ou): Optimization for the following model(s) did not converge: OU
These model fit(s) should not be considered reliable.
```

```
Total # hypotheses:  1 
1  

Comparing 6 models [n = 4, method = Joint]

                  logL K      AICc     dAICc Akaike.wt
GRW          -4.081278 3       Inf       Inf     0.000
URW          -4.090390 2  24.18078 51.905027     0.000
Stasis       -2.687149 2  21.37430 49.098545     0.000
StrictStasis -3.441416 1  10.88283 38.607079     0.000
Punc-1       -2.652483 4 -26.69503  1.029213     0.374
OU           -2.137876 4 -27.72425  0.000000     0.626
```

```
Total # hypotheses:  1 
1  

Comparing 6 models [n = 4, method = Joint]

                  logL K      AICc      dAICc Akaike.wt
GRW          -5.582037 3       Inf        Inf     0.000
URW          -5.589709 2  27.17942 53.3201715     0.000
Stasis       -3.593932 2  23.18786 49.3286176     0.000
StrictStasis -5.045351 1  14.09070 40.2314552     0.000
Punc-1       -2.929623 4 -26.14075  0.0000000     0.547
OU           -3.119443 4 -25.76111  0.3796397     0.453
```

```
Total # hypotheses:  1 
1  

Comparing 6 models [n = 4, method = Joint]

                    logL K       AICc     dAICc Akaike.wt
GRW           0.40152787 3        Inf       Inf     0.000
URW          -0.09249480 2  16.184990 49.371294     0.000
Stasis       -0.06070167 2  16.121403 49.307708     0.000
StrictStasis -0.06068035 1   4.121361 37.307666     0.000
Punc-1        0.09143985 4 -32.182880  1.003425     0.377
OU            0.59315240 4 -33.186305  0.000000     0.623
```

```
Total # hypotheses:  1 
1  

Comparing 6 models [n = 4, method = Joint]

                  logL K      AICc     dAICc Akaike.wt
GRW          -4.611405 3       Inf       Inf     0.000
URW          -6.627231 2  29.25446 52.693249     0.000
Stasis       -6.182646 2  28.36529 51.804079     0.000
StrictStasis -6.463189 1  16.92638 40.365165     0.000
Punc-1       -6.049755 4 -19.90049  3.538296     0.146
OU           -4.280607 4 -23.43879  0.000000     0.854
```

#### iii. Facial analyses of the *Homo sapiens* lineage

This follows the same structure as the previous analysis.

```
#1. Remove early Homo from dataset
sapiens_lineage_index_2aiii <- !face_data$SpeciesExpanded %in% c(
      "early Homo",
      "early H. neanderthalensis",
      "H. neanderthalensis"
      )

face_sapiens_nomiss_2aiii <- face_nomiss|>
  filter( #Filter out species not on H. sapiens lineage
    sapiens_lineage_index_2aiii
  )

#2. Calculate PCA
PCA_face_sapiens_2aiii <- PCA(face_sapiens_nomiss_2aiii[,2:ncol(face_nomiss)],face_sapiens_nomiss_2aiii[,1])

#A dataframe is created to facilitate ploting via ggplot
PCA_face_sapiens_plotdf_2aiii <- tibble(
  Specimen = face_sapiens_nomiss_2aiii$Specimen,
  Species =  factor(face_data$SpeciesExpanded[sapiens_lineage_index_2aiii],
                    levels = 
                       c("H. erectus",
                         "H. heidelbergensis s.l.", 
                         "early H. sapiens",
                         "Upper Paleolithic H. sapiens",
                         "H. sapiens")
  ),
  PC1 = PCA_face_sapiens_2aiii$PCS[,1],
  PC2 = PCA_face_sapiens_2aiii$PCS[,2],
  PC3 = PCA_face_sapiens_2aiii$PCS[,3],
  PC4 = PCA_face_sapiens_2aiii$PCS[,4],
  Centroid_size = face_gpa$Csize[sapiens_lineage_index_2aiii]
)

#Plot PC1
ggplot(PCA_face_sapiens_plotdf_2aiii, aes(y = PC1,x=Species))+
  geom_violin(aes(color=Species, fill = Species))+
  scale_colour_viridis_d(option="H")+
  geom_boxplot(width=0.1)+
  scale_fill_viridis_d(alpha=0.3,option="H")+
  labs(x = "PC1")
```

```
#Plot PC2
ggplot(PCA_face_sapiens_plotdf_2aiii, aes(y = PC2,x=Species))+
  geom_violin(aes(color=Species, fill = Species))+
  scale_colour_viridis_d(option="H")+
  geom_boxplot(width=0.1)+
  scale_fill_viridis_d(alpha=0.3,option="H")+
  labs(x = "PC2")
```

```
#Plot PC3
ggplot(PCA_face_sapiens_plotdf_2aiii, aes(y = PC3,x=Species))+
  geom_violin(aes(color=Species, fill = Species))+
  scale_colour_viridis_d(option="H")+
  geom_boxplot(width=0.1)+
  scale_fill_viridis_d(alpha=0.3,option="H")+
  labs(x = "PC3")
```

```
#Plot PC4
ggplot(PCA_face_sapiens_plotdf_2aiii, aes(y = PC4,x=Species))+
  geom_violin(aes(color=Species, fill = Species))+
  scale_colour_viridis_d(option="H")+
  geom_boxplot(width=0.1)+
  scale_fill_viridis_d(alpha=0.3,option="H")+
  labs(x = "PC4")
```

```
#Plot centroid size
ggplot(PCA_face_sapiens_plotdf_2aiii, aes(y = Centroid_size, x=Species))+
  geom_violin(aes(color=Species, fill = Species))+
  scale_colour_viridis_d(option="H")+
  geom_boxplot(width=0.1)+
  scale_fill_viridis_d(alpha=0.3,option="H")+
  labs(x = "Centroid Size")
```

```
#3 Evolutionary models

#Create vector with species assignation
species_sapiens_2aiii <- factor( face_data$SpeciesExpanded[sapiens_lineage_index_2aiii]
)
                           
#load chronology data
chronology_sapiens_2aiii <- taxa_chronology|>
  filter(
    !taxon %in% c(
      "early Homo",
      "early H. neanderthalensis", 
      "H. neanderthalensis"
      )
  )

#This function prepares the data to be passed to PaleoTS
PTS_PCS_sapiens_2aiii <- dists2PaleoTS(data.frame(PCA_face_sapiens_plotdf_2aiii[,c(1,3:7)]),species_sapiens_2aiii,chronology_sapiens_2aiii)

#This wrapper function will test the 6 evolutionary models considered in the article against each of the PCs and the centroid size. 
PTS_PCS_models_sapiens_2aiii <- multiple_paleoTS(PTS_PCS_sapiens_2aiii,models=6)
```

```
Total # hypotheses:  2 
1  2  

Comparing 6 models [n = 5, method = Joint]

                   logL K     AICc       dAICc Akaike.wt
GRW           -3.816371 3 37.63274 16.61690740     0.000
URW           -5.533252 2 21.06650  0.05066862     0.494
Stasis        -5.507917 2 21.01583  0.00000000     0.506
StrictStasis -17.739288 1 38.81191 17.79607476     0.000
Punc-1        -2.114328 4      Inf         Inf     0.000
OU            -2.780192 4      Inf         Inf     0.000
```

```
Total # hypotheses:  2 
1  2  

Comparing 6 models [n = 5, method = Joint]

                  logL K      AICc     dAICc Akaike.wt
GRW          -2.673380 3 35.346760 25.543917     0.000
URW          -3.297927 2 16.595854  6.793012     0.031
Stasis       -3.222004 2 16.444008  6.641165     0.034
StrictStasis -3.234755 1  9.802843  0.000000     0.935
Punc-1       -3.128528 4       Inf       Inf     0.000
OU           -2.119508 4       Inf       Inf     0.000
```

```
Total # hypotheses:  2 
1  2  

Comparing 6 models [n = 5, method = Joint]

                   logL K      AICc       dAICc Akaike.wt
GRW           -5.875607 3  41.75121  20.3156988     0.000
URW           -6.078168 2  22.15634   0.7208211     0.411
Stasis        -5.717758 2  21.43552   0.0000000     0.589
StrictStasis -67.112248 1 137.55783 116.1223137     0.000
Punc-1        -1.101722 4       Inf         Inf     0.000
OU            -4.461507 4       Inf         Inf     0.000
```

```
Total # hypotheses:  2 
1  2  

Comparing 6 models [n = 5, method = Joint]

                  logL K     AICc      dAICc Akaike.wt
GRW          -5.848845 3 41.69769 20.4880470     0.000
URW          -6.051933 2 22.10387  0.8942222     0.260
Stasis       -5.604822 2 21.20964  0.0000000     0.407
StrictStasis -9.138695 1 21.61072  0.4010797     0.333
Punc-1       -4.180659 4      Inf        Inf     0.000
OU           -3.696102 4      Inf        Inf     0.000
```

```
Total # hypotheses:  2 
1  2  

Comparing 6 models [n = 5, method = Joint]

                   logL K      AICc      dAICc Akaike.wt
GRW           -9.835542 3  49.67108 19.6228262     0.000
URW          -10.024129 2  30.04826  0.0000000     0.616
Stasis       -10.497410 2  30.99482  0.9465624     0.384
StrictStasis -50.913499 1 105.16033 75.1120734     0.000
Punc-1        -5.159774 4       Inf        Inf     0.000
OU            -9.148658 4       Inf        Inf     0.000
```

#### iv. Facial analysis of the *Homo neanderthalensis* lineage

This follows the same structure as the previous analysis.

```
#1. Remove early Homo from dataset
neandertal_lineage_index_2aiv <- !face_data$SpeciesExpanded %in% c(
  "early Homo",
  "early H. sapiens",
  "Upper Paleolithic H. sapiens",
  "H. sapiens"
      )

face_neandertal_nomiss_2aiv <- face_nomiss|>
  filter( #Filter out species not on H. neanderthalensis lineage
    neandertal_lineage_index_2aiv
  )

#2. Calculate PCA
PCA_face_neandertal_2aiv <- PCA(face_neandertal_nomiss_2aiv[,2:ncol(face_nomiss)],face_neandertal_nomiss_2aiv[,1])

#A dataframe is created to facilitate ploting via ggplot
PCA_face_neandertal_plotdf_2aiv <- tibble(
  Specimen = face_neandertal_nomiss_2aiv$Specimen,
  Species =  factor(face_data$SpeciesExpanded[neandertal_lineage_index_2aiv],
                    levels = 
                       c("H. erectus",
                         "H. heidelbergensis s.l.", 
                         "early H. neanderthalensis",
                         "H. neanderthalensis")
  ),
  PC1 = PCA_face_neandertal_2aiv$PCS[,1],
  PC2 = PCA_face_neandertal_2aiv$PCS[,2],
  PC3 = PCA_face_neandertal_2aiv$PCS[,3],
  PC4 = PCA_face_neandertal_2aiv$PCS[,4],
  Centroid_size = face_gpa$Csize[neandertal_lineage_index_2aiv]
)

#Plot  PC1
ggplot(PCA_face_neandertal_plotdf_2aiv, aes(y = PC1,x=Species))+
  geom_violin(aes(color=Species, fill = Species))+
  scale_colour_viridis_d(option="H")+
  geom_boxplot(width=0.1)+
  scale_fill_viridis_d(alpha=0.3,option="H")+
  labs(x = "PC1")
```

```
#Plot PC2
ggplot(PCA_face_neandertal_plotdf_2aiv, aes(y = PC2,x=Species))+
  geom_violin(aes(color=Species, fill = Species))+
  scale_colour_viridis_d(option="H")+
  geom_boxplot(width=0.1)+
  scale_fill_viridis_d(alpha=0.3,option="H")+
  labs(x = "PC2")
```

```
#Plot PC3
ggplot(PCA_face_neandertal_plotdf_2aiv, aes(y = PC3,x=Species))+
  geom_violin(aes(color=Species, fill = Species))+
  scale_colour_viridis_d(option="H")+
  geom_boxplot(width=0.1)+
  scale_fill_viridis_d(alpha=0.3,option="H")+
  labs(x = "PC3")
```

```
#Plot PC4
ggplot(PCA_face_neandertal_plotdf_2aiv, aes(y = PC4,x=Species))+
  geom_violin(aes(color=Species, fill = Species))+
  scale_colour_viridis_d(option="H")+
  geom_boxplot(width=0.1)+
  scale_fill_viridis_d(alpha=0.3,option="H")+
  labs(x = "PC4")
```

```
#Plot centroid size
ggplot(PCA_face_neandertal_plotdf_2aiv, aes(y = Centroid_size, x=Species))+
  geom_violin(aes(color=Species, fill = Species))+
  scale_colour_viridis_d(option="H")+
  geom_boxplot(width=0.1)+
  scale_fill_viridis_d(alpha=0.3,option="H")+
  labs(x = "Centroid Size")
```

```
#3 Evolutionary models

#Create vector with species assignation
species_neandertal_2aiv <- factor( face_data$SpeciesExpanded[neandertal_lineage_index_2aiv]
)
                           
#load chronology data
chronology_neandertal_2aiv <- taxa_chronology|>
  filter(
    !taxon %in% c(
      "early Homo",
      "early H. sapiens",
      "Upper Paleolithic H. sapiens",
      "H. sapiens"
      )
  )

#This function prepares the data to be passed to PaleoTS

####PCs 1 and 3 ARE RETURNING CALCULATION ERRORS IN THE EVOLUTIONARY MODEL TESTS, SO IT WAS REMOVED FROM THIS ANALYSIS.

PTS_PCS_neandertal_2aiv <- dists2PaleoTS(data.frame(PCA_face_neandertal_plotdf_2aiv[,c(1,4:5,7)]),species_neandertal_2aiv,chronology_neandertal_2aiv)

#This wrapper function will test the 6 evolutionary models considered in the article against each of the PCs and the centroid size. 
PTS_PCS_models_neandertal_2aiv <- multiple_paleoTS(PTS_PCS_neandertal_2aiv,models=6)
```

```
Total # hypotheses:  1 
1  

Comparing 6 models [n = 4, method = Joint]

                   logL K      AICc     dAICc Akaike.wt
GRW          -2.0039058 3       Inf       Inf     0.000
URW          -3.7741266 2  23.54825 56.246493     0.000
Stasis       -4.7502853 2  25.50057 58.198810     0.000
StrictStasis -6.8416831 1  17.68337 50.381606     0.000
Punc-1       -4.3638880 4 -23.27222  9.426016     0.009
OU            0.3491198 4 -32.69824  0.000000     0.991
```

```
Total # hypotheses:  1 
1  

Comparing 6 models [n = 4, method = Joint]

                  logL K       AICc      dAICc Akaike.wt
GRW          -1.700541 3        Inf        Inf      0.00
URW          -1.738160 2  19.476320 49.0083724      0.00
Stasis       -1.722117 2  19.444234 48.9762856      0.00
StrictStasis -1.722112 1   7.444225 36.9762768      0.00
Punc-1       -1.233974 4 -29.532052  0.0000000      0.59
OU           -1.596880 4 -28.806239  0.7258128      0.41
```

```
Total # hypotheses:  1 
1  

Comparing 6 models [n = 4, method = Joint]

                  logL K      AICc      dAICc Akaike.wt
GRW          -3.906665 3       Inf        Inf      0.00
URW          -3.925331 2  23.85066 48.6496725      0.00
Stasis       -3.916021 2  23.83204 48.6310513      0.00
StrictStasis -3.916018 1  11.83204 36.6310457      0.00
Punc-1       -3.600495 4 -24.79901  0.0000000      0.54
OU           -3.759965 4 -24.48007  0.3189395      0.46
```

### 2b. early *Homo* only includes *H. habilis* specimen

In this scenario, the *H. habilis* specimen available for analysis (KNM ER 1813) is assumed to represent the average morphology of the early *Homo* OTU.

#### i. Neurocranial analyses of the *Homo sapiens* lineage

The same set of analyses done in section 2a are replicated here, starting with the removal of *H. rudolphensis*, followed by PCA, and evolutionary models.

```
#1. Remove H. rudolphensis from dataset
sapiens_lineage_index_2bi <-  !neurocranium_data$SpeciesExpanded %in% c(
      "early H. neanderthalensis",
      "H. neanderthalensis"
      ) & neurocranium_data$Specimen != "KNM ER 1813"


neuro_sapiens_nomiss_2bi <- neuro_nomiss|>
  filter( #Filter out species not on H. sapiens lineage
    sapiens_lineage_index_2bi
  )

#2. Calculate PCA
PCA_neuro_sapiens_2bi <- PCA(neuro_sapiens_nomiss_2bi[,2:ncol(neuro_nomiss)],neuro_sapiens_nomiss_2bi[,1])

#A dataframe is created to facilitate ploting via ggplot
PCA_neuro_sapiens_plotdf_2bi <- tibble(
  Specimen = neuro_sapiens_nomiss_2bi$Specimen,
  Species =  factor(neurocranium_data$SpeciesExpanded[sapiens_lineage_index_2bi],
                    levels = 
                       c("early Homo",
                         "H. erectus",
                         "H. heidelbergensis s.l.", 
                         "early H. sapiens",
                         "Upper Paleolithic H. sapiens",
                         "H. sapiens")
  ),
  PC1 = PCA_neuro_sapiens_2bi$PCS[,1],
  PC2 = PCA_neuro_sapiens_2bi$PCS[,2],
  PC3 = PCA_neuro_sapiens_2bi$PCS[,3],
  PC4 = PCA_neuro_sapiens_2bi$PCS[,4],
  Centroid_size = neuro_gpa$Csize[sapiens_lineage_index_2bi]
)

#Plot PC1
ggplot(PCA_neuro_sapiens_plotdf_2bi, aes(y = PC1,x=Species))+
  geom_violin(aes(color=Species, fill = Species))+
  scale_colour_viridis_d(option="H")+
  geom_boxplot(width=0.1)+
  scale_fill_viridis_d(alpha=0.3,option="H")+
  labs(x = "PC1")
```

```
Warning: Groups with fewer than two datapoints have been dropped.
ℹ Set `drop = FALSE` to consider such groups for position adjustment purposes.
```

```
#Plot PC2
ggplot(PCA_neuro_sapiens_plotdf_2bi, aes(y = PC2,x=Species))+
  geom_violin(aes(color=Species, fill = Species))+
  scale_colour_viridis_d(option="H")+
  geom_boxplot(width=0.1)+
  scale_fill_viridis_d(alpha=0.3,option="H")+
  labs(x = "PC2")
```

```
Warning: Groups with fewer than two datapoints have been dropped.
ℹ Set `drop = FALSE` to consider such groups for position adjustment purposes.
```

```
#Plot PC3
ggplot(PCA_neuro_sapiens_plotdf_2bi, aes(y = PC3,x=Species))+
  geom_violin(aes(color=Species, fill = Species))+
  scale_colour_viridis_d(option="H")+
  geom_boxplot(width=0.1)+
  scale_fill_viridis_d(alpha=0.3,option="H")+
  labs(x = "PC3")
```

```
Warning: Groups with fewer than two datapoints have been dropped.
ℹ Set `drop = FALSE` to consider such groups for position adjustment purposes.
```

```
#Plot PC4
ggplot(PCA_neuro_sapiens_plotdf_2bi, aes(y = PC4,x=Species))+
  geom_violin(aes(color=Species, fill = Species))+
  scale_colour_viridis_d(option="H")+
  geom_boxplot(width=0.1)+
  scale_fill_viridis_d(alpha=0.3,option="H")+
  labs(x = "PC4")
```

```
Warning: Groups with fewer than two datapoints have been dropped.
ℹ Set `drop = FALSE` to consider such groups for position adjustment purposes.
```

```
#Plot centroid size
ggplot(PCA_neuro_sapiens_plotdf_2bi, aes(y = Centroid_size, x=Species))+
  geom_violin(aes(color=Species, fill = Species))+
  scale_colour_viridis_d(option="H")+
  geom_boxplot(width=0.1)+
  scale_fill_viridis_d(alpha=0.3,option="H")+
  labs(x = "Centroid Size")
```

```
Warning: Groups with fewer than two datapoints have been dropped.
ℹ Set `drop = FALSE` to consider such groups for position adjustment purposes.
```

```
#3 Evolutionary models

#Create vector with species assignation
species_sapiens_2bi <- factor( neurocranium_data$SpeciesExpanded[sapiens_lineage_index_2bi]
)
                           
#load chronology data
chronology_sapiens_2bi <- taxa_chronology|>
  filter(
    !taxon %in% c(
      "early H. neanderthalensis", 
      "H. neanderthalensis"
      )
  )

#This function prepares the data to be passed to PaleoTS
PTS_PCS_sapiens_2bi <- dists2PaleoTS(data.frame(PCA_neuro_sapiens_plotdf_2bi[,c(1,3:7)]),species_sapiens_2bi,chronology_sapiens_2bi)
```

```
Warning in dists2PaleoTS(data.frame(PCA_neuro_sapiens_plotdf_2bi[, c(1, : early
Homo has N=1. Weigthed average variance used for it.
```

```
Warning in dists2PaleoTS(data.frame(PCA_neuro_sapiens_plotdf_2bi[, c(1, : early
Homo has N=1. Weigthed average variance used for it.
Warning in dists2PaleoTS(data.frame(PCA_neuro_sapiens_plotdf_2bi[, c(1, : early
Homo has N=1. Weigthed average variance used for it.
Warning in dists2PaleoTS(data.frame(PCA_neuro_sapiens_plotdf_2bi[, c(1, : early
Homo has N=1. Weigthed average variance used for it.
Warning in dists2PaleoTS(data.frame(PCA_neuro_sapiens_plotdf_2bi[, c(1, : early
Homo has N=1. Weigthed average variance used for it.
```

```
#This wrapper function will test the 6 evolutionary models considered in the article against each of the PCs and the centroid size.
PTS_PCS_models_sapiens_2bi <- multiple_paleoTS(PTS_PCS_sapiens_2bi,models=6)
```

```
Total # hypotheses:  3 
1  2  3  

Comparing 6 models [n = 6, method = Joint]

                    logL K      AICc      dAICc Akaike.wt
GRW            -3.585481 3  25.17096   6.805233     0.032
URW            -5.182865 2  18.36573   0.000000     0.951
Stasis         -9.201186 2  26.40237   8.036643     0.017
StrictStasis -125.180263 1 253.36053 234.994795     0.000
Punc-1         -3.113674 4  54.22735  35.861617     0.000
OU             -3.588502 4  55.17700  36.811274     0.000
```

```
Warning in compareModels(grw, urw, sta, strsta, pun2, ou): Optimization for the following model(s) did not converge: OU
These model fit(s) should not be considered reliable.
```

```
Total # hypotheses:  3 
1  2  3  

Comparing 6 models [n = 6, method = Joint]

                   logL K     AICc     dAICc Akaike.wt
GRW           -7.488687 3 32.97737  9.203625     0.009
URW           -7.886875 2 23.77375  0.000000     0.858
Stasis        -9.841408 2 27.68282  3.909067     0.121
StrictStasis -14.630611 1 32.26122  8.487471     0.012
Punc-1        -7.072959 4 62.14592 38.372168     0.000
OU            -3.168744 4 54.33749 30.563738     0.000
```

```
Total # hypotheses:  3 
1  2  3  

Comparing 6 models [n = 6, method = Joint]

                  logL K     AICc     dAICc Akaike.wt
GRW          -4.447433 3 26.89487 12.419548     0.002
URW          -5.832074 2 19.66415  5.188829     0.062
Stasis       -3.237659 2 14.47532  0.000000     0.824
StrictStasis -7.724409 1 18.44882  3.973499     0.113
Punc-1       -1.970557 4 51.94111 37.465794     0.000
OU           -3.183893 4 54.36779 39.892467     0.000
```

```
Total # hypotheses:  3 
1  2  3  

Comparing 6 models [n = 6, method = Joint]

                  logL K     AICc     dAICc Akaike.wt
GRW          -3.592742 3 25.18548 15.071299     0.000
URW          -3.620619 2 15.24124  5.127053     0.064
Stasis       -3.117240 2 14.23448  4.120294     0.106
StrictStasis -3.557093 1 10.11419  0.000000     0.830
Punc-1       -2.470977 4 52.94195 42.827768     0.000
OU           -2.516338 4 53.03268 42.918491     0.000
```

```
Total # hypotheses:  3 
1  2  3  

Comparing 6 models [n = 6, method = Joint]

                   logL K     AICc     dAICc Akaike.wt
GRW          -12.821195 3 43.64239 14.210656     0.001
URW          -12.833135 2 33.66627  4.234535     0.107
Stasis       -10.715867 2 29.43173  0.000000     0.892
StrictStasis -21.932586 1 46.86517 17.433437     0.000
Punc-1        -9.014697 4 66.02939 36.597659     0.000
OU           -10.094479 4 68.18896 38.757224     0.000
```

#### ii. Neurocranial analysis of the *Homo neanderthalensis* lineage

This follows the same structure as the previous analysis.

```
#1. Remove H. rudolphensis from dataset
neandertal_lineage_index_2bii <- !neurocranium_data$SpeciesExpanded %in% c(
  "early H. sapiens",
  "Upper Paleolithic H. sapiens",
  "H. sapiens"
      ) & neurocranium_data$Specimen != "KNM ER 1813"

neuro_neandertal_nomiss_2bii <- neuro_nomiss|>
  filter( #Filter out species not on H. neanderthalensis lineage
    neandertal_lineage_index_2bii
  )

#2. Calculate PCA
PCA_neuro_neandertal_2bii <- PCA(neuro_neandertal_nomiss_2bii[,2:ncol(neuro_nomiss)],neuro_neandertal_nomiss_2bii[,1])

#A dataframe is created to facilitate ploting via ggplot
PCA_neuro_neandertal_plotdf_2bii <- tibble(
  Specimen = neuro_neandertal_nomiss_2bii$Specimen,
  Species =  factor(neurocranium_data$SpeciesExpanded[neandertal_lineage_index_2bii],
                    levels = 
                       c("early Homo",
                         "H. erectus",
                         "H. heidelbergensis s.l.", 
                         "early H. neanderthalensis",
                         "H. neanderthalensis")
  ),
  PC1 = PCA_neuro_neandertal_2bii$PCS[,1],
  PC2 = PCA_neuro_neandertal_2bii$PCS[,2],
  PC3 = PCA_neuro_neandertal_2bii$PCS[,3],
  PC4 = PCA_neuro_neandertal_2bii$PCS[,4],
  Centroid_size = neuro_gpa$Csize[neandertal_lineage_index_2bii]
)

#Plot PC1
ggplot(PCA_neuro_neandertal_plotdf_2bii, aes(y = PC1,x=Species))+
  geom_violin(aes(color=Species, fill = Species))+
  scale_colour_viridis_d(option="H")+
  geom_boxplot(width=0.1)+
  scale_fill_viridis_d(alpha=0.3,option="H")+
  labs(x = "PC1")
```

```
Warning: Groups with fewer than two datapoints have been dropped.
ℹ Set `drop = FALSE` to consider such groups for position adjustment purposes.
```

```
#Plot PC2
ggplot(PCA_neuro_neandertal_plotdf_2bii, aes(y = PC2,x=Species))+
  geom_violin(aes(color=Species, fill = Species))+
  scale_colour_viridis_d(option="H")+
  geom_boxplot(width=0.1)+
  scale_fill_viridis_d(alpha=0.3,option="H")+
  labs(x = "PC2")
```

```
Warning: Groups with fewer than two datapoints have been dropped.
ℹ Set `drop = FALSE` to consider such groups for position adjustment purposes.
```

```
#Plot PC3
ggplot(PCA_neuro_neandertal_plotdf_2bii, aes(y = PC3,x=Species))+
  geom_violin(aes(color=Species, fill = Species))+
  scale_colour_viridis_d(option="H")+
  geom_boxplot(width=0.1)+
  scale_fill_viridis_d(alpha=0.3,option="H")+
  labs(x = "PC3")
```

```
Warning: Groups with fewer than two datapoints have been dropped.
ℹ Set `drop = FALSE` to consider such groups for position adjustment purposes.
```

```
#Plot PC4
ggplot(PCA_neuro_neandertal_plotdf_2bii, aes(y = PC4,x=Species))+
  geom_violin(aes(color=Species, fill = Species))+
  scale_colour_viridis_d(option="H")+
  geom_boxplot(width=0.1)+
  scale_fill_viridis_d(alpha=0.3,option="H")+
  labs(x = "PC4")
```

```
Warning: Groups with fewer than two datapoints have been dropped.
ℹ Set `drop = FALSE` to consider such groups for position adjustment purposes.
```

```
#Plot centroid size
ggplot(PCA_neuro_neandertal_plotdf_2bii, aes(y = Centroid_size, x=Species))+
  geom_violin(aes(color=Species, fill = Species))+
  scale_colour_viridis_d(option="H")+
  geom_boxplot(width=0.1)+
  scale_fill_viridis_d(alpha=0.3,option="H")+
  labs(x = "Centroid Size")
```

```
Warning: Groups with fewer than two datapoints have been dropped.
ℹ Set `drop = FALSE` to consider such groups for position adjustment purposes.
```

```
#3 Evolutionary models

#Create vector with species assignation
species_neandertal_2bii <- factor( neurocranium_data$SpeciesExpanded[neandertal_lineage_index_2bii]
)
                           
#load chronology data
chronology_neandertal_2bii <- taxa_chronology|>
  filter(
    !taxon %in% c(
      "early H. sapiens",
      "Upper Paleolithic H. sapiens",
      "H. sapiens"
      )
  )

#This function prepares the data to be passed to PaleoTS
PTS_PCS_neandertal_2bii <- dists2PaleoTS(data.frame(PCA_neuro_neandertal_plotdf_2bii[,c(1,3:7)]),species_neandertal_2bii,chronology_neandertal_2bii)
```

```
Warning in dists2PaleoTS(data.frame(PCA_neuro_neandertal_plotdf_2bii[, c(1, :
early Homo has N=1. Weigthed average variance used for it.
```

```
Warning in dists2PaleoTS(data.frame(PCA_neuro_neandertal_plotdf_2bii[, c(1, :
early Homo has N=1. Weigthed average variance used for it.
Warning in dists2PaleoTS(data.frame(PCA_neuro_neandertal_plotdf_2bii[, c(1, :
early Homo has N=1. Weigthed average variance used for it.
Warning in dists2PaleoTS(data.frame(PCA_neuro_neandertal_plotdf_2bii[, c(1, :
early Homo has N=1. Weigthed average variance used for it.
Warning in dists2PaleoTS(data.frame(PCA_neuro_neandertal_plotdf_2bii[, c(1, :
early Homo has N=1. Weigthed average variance used for it.
```

```
#This wrapper function will test the 6 evolutionary models considered in the article against each of the PCs and the centroid size. 
PTS_PCS_models_neandertal_2bii <- multiple_paleoTS(PTS_PCS_neandertal_2bii,models=6)
```

```
Total # hypotheses:  2 
1  2  

Comparing 6 models [n = 5, method = Joint]

                    logL K      AICc      dAICc Akaike.wt
GRW           -0.8823501 3  31.76470  11.377513     0.003
URW           -5.1935935 2  20.38719   0.000000     0.947
Stasis        -8.1390755 2  26.27815   5.890964     0.050
StrictStasis -62.2360007 1 127.80533 107.418148     0.000
Punc-1        -3.5980693 4       Inf        Inf     0.000
OU             0.8606699 4       Inf        Inf     0.000
```

```
Total # hypotheses:  2 
1  2  

Comparing 6 models [n = 5, method = Joint]

                  logL K     AICc     dAICc Akaike.wt
GRW          -6.670858 3 43.34172 25.337260     0.000
URW          -6.685766 2 23.37153  5.367076     0.050
Stasis       -5.237511 2 20.47502  2.470567     0.214
StrictStasis -7.335561 1 18.00446  0.000000     0.736
Punc-1       -4.903853 4      Inf       Inf     0.000
OU           -3.754430 4      Inf       Inf     0.000
```

```
Total # hypotheses:  2 
1  2  

Comparing 6 models [n = 5, method = Joint]

                  logL K     AICc     dAICc Akaike.wt
GRW          -4.643696 3 39.28739 26.418645     0.000
URW          -4.855949 2 19.71190  6.843152     0.028
Stasis       -3.512377 2 17.02475  4.156008     0.108
StrictStasis -4.767706 1 12.86875  0.000000     0.864
Punc-1       -3.496014 4      Inf       Inf     0.000
OU           -2.134763 4      Inf       Inf     0.000
```

```
Total # hypotheses:  2 
1  2  

Comparing 6 models [n = 5, method = Joint]

                  logL K     AICc     dAICc Akaike.wt
GRW          -4.486515 3 38.97303 25.258220     0.000
URW          -5.207422 2 20.41484  6.700033     0.032
Stasis       -4.571803 2 19.14361  5.428795     0.060
StrictStasis -5.190739 1 13.71481  0.000000     0.908
Punc-1       -3.693892 4      Inf       Inf     0.000
OU           -4.467870 4      Inf       Inf     0.000
```

```
Warning in compareModels(grw, urw, sta, strsta, pun2, ou): Optimization for the following model(s) did not converge: OU
These model fit(s) should not be considered reliable.
```

```
Total # hypotheses:  2 
1  2  

Comparing 6 models [n = 5, method = Joint]

                  logL K     AICc     dAICc Akaike.wt
GRW          -6.231582 3 42.46316 21.605924     0.000
URW          -8.612142 2 27.22428  6.367044     0.038
Stasis       -8.411578 2 26.82316  5.965916     0.046
StrictStasis -8.761953 1 20.85724  0.000000     0.916
Punc-1       -5.905099 4      Inf       Inf     0.000
OU           -6.082105 4      Inf       Inf     0.000
```

#### iii. Facial analyses of the *Homo sapiens* lineage

This follows the same structure as the previous analysis.

```
#1. Remove Homo rudolphensis from dataset
sapiens_lineage_index_2biii <- !face_data$SpeciesExpanded %in% c(
      "early H. neanderthalensis",
      "H. neanderthalensis"
      )& face_data$Specimen != "KNM ER 1813"

face_sapiens_nomiss_2biii <- face_nomiss|>
  filter( #Filter out species not on H. sapiens lineage
    sapiens_lineage_index_2biii
  )

#2. Calculate PCA
PCA_face_sapiens_2biii <- PCA(face_sapiens_nomiss_2biii[,2:ncol(face_nomiss)],face_sapiens_nomiss_2biii[,1])

#A dataframe is created to facilitate ploting via ggplot
PCA_face_sapiens_plotdf_2biii <- tibble(
  Specimen = face_sapiens_nomiss_2biii$Specimen,
  Species =  factor(face_data$SpeciesExpanded[sapiens_lineage_index_2biii],
                    levels = 
                       c("early Homo",
                         "H. erectus",
                         "H. heidelbergensis s.l.", 
                         "early H. sapiens",
                         "Upper Paleolithic H. sapiens",
                         "H. sapiens")
  ),
  PC1 = PCA_face_sapiens_2biii$PCS[,1],
  PC2 = PCA_face_sapiens_2biii$PCS[,2],
  PC3 = PCA_face_sapiens_2biii$PCS[,3],
  PC4 = PCA_face_sapiens_2biii$PCS[,4],
  Centroid_size = face_gpa$Csize[sapiens_lineage_index_2biii]
)

#Plot PC1
ggplot(PCA_face_sapiens_plotdf_2biii, aes(y = PC1,x=Species))+
  geom_violin(aes(color=Species, fill = Species))+
  scale_colour_viridis_d(option="H")+
  geom_boxplot(width=0.1)+
  scale_fill_viridis_d(alpha=0.3,option="H")+
  labs(x = "PC1")
```

```
Warning: Groups with fewer than two datapoints have been dropped.
ℹ Set `drop = FALSE` to consider such groups for position adjustment purposes.
```

```
#Plot PC2
ggplot(PCA_face_sapiens_plotdf_2biii, aes(y = PC2,x=Species))+
  geom_violin(aes(color=Species, fill = Species))+
  scale_colour_viridis_d(option="H")+
  geom_boxplot(width=0.1)+
  scale_fill_viridis_d(alpha=0.3,option="H")+
  labs(x = "PC2")
```

```
Warning: Groups with fewer than two datapoints have been dropped.
ℹ Set `drop = FALSE` to consider such groups for position adjustment purposes.
```

```
#Plot PC3
ggplot(PCA_face_sapiens_plotdf_2biii, aes(y = PC3,x=Species))+
  geom_violin(aes(color=Species, fill = Species))+
  scale_colour_viridis_d(option="H")+
  geom_boxplot(width=0.1)+
  scale_fill_viridis_d(alpha=0.3,option="H")+
  labs(x = "PC3")
```

```
Warning: Groups with fewer than two datapoints have been dropped.
ℹ Set `drop = FALSE` to consider such groups for position adjustment purposes.
```

```
#Plot PC4
ggplot(PCA_face_sapiens_plotdf_2biii, aes(y = PC4,x=Species))+
  geom_violin(aes(color=Species, fill = Species))+
  scale_colour_viridis_d(option="H")+
  geom_boxplot(width=0.1)+
  scale_fill_viridis_d(alpha=0.3,option="H")+
  labs(x = "PC4")
```

```
Warning: Groups with fewer than two datapoints have been dropped.
ℹ Set `drop = FALSE` to consider such groups for position adjustment purposes.
```

```
#Plot centroid size
ggplot(PCA_face_sapiens_plotdf_2biii, aes(y = Centroid_size, x=Species))+
  geom_violin(aes(color=Species, fill = Species))+
  scale_colour_viridis_d(option="H")+
  geom_boxplot(width=0.1)+
  scale_fill_viridis_d(alpha=0.3,option="H")+
  labs(x = "Centroid Size")
```

```
Warning: Groups with fewer than two datapoints have been dropped.
ℹ Set `drop = FALSE` to consider such groups for position adjustment purposes.
```

```
#3 Evolutionary models

#Create vector with species assignation
species_sapiens_2biii <- factor( face_data$SpeciesExpanded[sapiens_lineage_index_2biii]
)
                           
#load chronology data
chronology_sapiens_2biii <- taxa_chronology|>
  filter(
    !taxon %in% c(
      "early H. neanderthalensis", 
      "H. neanderthalensis"
      )
  )

#This function prepares the data to be passed to PaleoTS
PTS_PCS_sapiens_2biii <- dists2PaleoTS(data.frame(PCA_face_sapiens_plotdf_2biii[,c(1,3:7)]),species_sapiens_2biii,chronology_sapiens_2biii)
```

```
Warning in dists2PaleoTS(data.frame(PCA_face_sapiens_plotdf_2biii[, c(1, :
early Homo has N=1. Weigthed average variance used for it.
```

```
Warning in dists2PaleoTS(data.frame(PCA_face_sapiens_plotdf_2biii[, c(1, :
early Homo has N=1. Weigthed average variance used for it.
Warning in dists2PaleoTS(data.frame(PCA_face_sapiens_plotdf_2biii[, c(1, :
early Homo has N=1. Weigthed average variance used for it.
Warning in dists2PaleoTS(data.frame(PCA_face_sapiens_plotdf_2biii[, c(1, :
early Homo has N=1. Weigthed average variance used for it.
Warning in dists2PaleoTS(data.frame(PCA_face_sapiens_plotdf_2biii[, c(1, :
early Homo has N=1. Weigthed average variance used for it.
```

```
#This wrapper function will test the 6 evolutionary models considered in the article against each of the PCs and the centroid size.
PTS_PCS_models_sapiens_2biii <- multiple_paleoTS(PTS_PCS_sapiens_2biii,models=6)
```

```
Total # hypotheses:  3 
1  2  3  

Comparing 6 models [n = 6, method = Joint]

                   logL K     AICc     dAICc Akaike.wt
GRW           -4.764861 3 27.52972  4.042749     0.079
URW           -7.743486 2 23.48697  0.000000     0.593
Stasis        -8.335998 2 24.67200  1.185023     0.328
StrictStasis -19.286035 1 41.57207 18.085097     0.000
Punc-1        -4.405536 4 56.81107 33.324098     0.000
OU            -5.189738 4 58.37948 34.892503     0.000
```

```
Total # hypotheses:  3 
1  2  3  

Comparing 6 models [n = 6, method = Joint]

                  logL K     AICc     dAICc Akaike.wt
GRW          -4.143415 3 26.28683 13.583372     0.001
URW          -4.922644 2 17.84529  5.141829     0.066
Stasis       -4.837600 2 17.67520  4.971742     0.072
StrictStasis -4.851729 1 12.70346  0.000000     0.861
Punc-1       -3.676730 4 55.35346 42.650001     0.000
OU           -4.062677 4 56.12535 43.421896     0.000
```

```
Total # hypotheses:  3 
1  2  3  

Comparing 6 models [n = 6, method = Joint]

                   logL K      AICc       dAICc Akaike.wt
GRW           -9.582121 3  37.16424  10.4236478     0.003
URW           -9.614407 2  27.22881   0.4882206     0.438
Stasis        -9.370297 2  26.74059   0.0000000     0.559
StrictStasis -90.723506 1 184.44701 157.7064178     0.000
Punc-1        -8.780396 4  65.56079  38.8201984     0.000
OU            -5.914794 4  59.82959  33.0889946     0.000
```

```
Total # hypotheses:  3 
1  2  3  

Comparing 6 models [n = 6, method = Joint]

                  logL K     AICc     dAICc Akaike.wt
GRW          -7.443415 3 32.88683 11.439869     0.002
URW          -7.443499 2 22.88700  1.440038     0.240
Stasis       -6.723480 2 21.44696  0.000000     0.493
StrictStasis -9.843752 1 22.68750  1.240543     0.265
Punc-1       -4.248584 4 56.49717 35.050209     0.000
OU           -6.192206 4 60.38441 38.937452     0.000
```

```
Total # hypotheses:  3 
1  2  3  

Comparing 6 models [n = 6, method = Joint]

                   logL K      AICc      dAICc Akaike.wt
GRW          -11.665626 3  41.33125  9.6769287     0.005
URW          -11.827162 2  31.65432  0.0000000     0.614
Stasis       -12.304410 2  32.60882  0.9544957     0.381
StrictStasis -54.370506 1 111.74101 80.0866871     0.000
Punc-1        -6.303181 4  60.60636 28.9520380     0.000
OU           -10.740801 4  69.48160 37.8272772     0.000
```

#### iv. Facial analysis of the *Homo neanderthalensis* lineage

This follows the same structure as the previous analysis.

```
#1. Remove Homo rudolphensis from the dataset
neandertal_lineage_index_2biv <- !face_data$SpeciesExpanded %in% c(
  "early H. sapiens",
  "Upper Paleolithic H. sapiens",
  "H. sapiens"
      )& face_data$Specimen != "KNM ER 1813"

face_neandertal_nomiss_2biv <- face_nomiss|>
  filter( #Filter out species not on H. neanderthalensis lineage
    neandertal_lineage_index_2biv
  )

#2. Calculate PCA
PCA_face_neandertal_2biv <- PCA(face_neandertal_nomiss_2biv[,2:ncol(face_nomiss)],face_neandertal_nomiss_2biv[,1])

#A dataframe is created to facilitate ploting via ggplot
PCA_face_neandertal_plotdf_2biv <- tibble(
  Specimen = face_neandertal_nomiss_2biv$Specimen,
  Species =  factor(face_data$SpeciesExpanded[neandertal_lineage_index_2biv],
                    levels = 
                       c("early Homo",
                         "H. erectus",
                         "H. heidelbergensis s.l.", 
                         "early H. neanderthalensis",
                         "H. neanderthalensis")
  ),
  PC1 = PCA_face_neandertal_2biv$PCS[,1],
  PC2 = PCA_face_neandertal_2biv$PCS[,2],
  PC3 = PCA_face_neandertal_2biv$PCS[,3],
  PC4 = PCA_face_neandertal_2biv$PCS[,4],
  Centroid_size = face_gpa$Csize[neandertal_lineage_index_2biv]
)

#Plot PC1
ggplot(PCA_face_neandertal_plotdf_2biv, aes(y = PC1,x=Species))+
  geom_violin(aes(color=Species, fill = Species))+
  scale_colour_viridis_d(option="H")+
  geom_boxplot(width=0.1)+
  scale_fill_viridis_d(alpha=0.3,option="H")+
  labs(x = "PC1")
```

```
Warning: Groups with fewer than two datapoints have been dropped.
ℹ Set `drop = FALSE` to consider such groups for position adjustment purposes.
```

```
#Plot PC2
ggplot(PCA_face_neandertal_plotdf_2biv, aes(y = PC2,x=Species))+
  geom_violin(aes(color=Species, fill = Species))+
  scale_colour_viridis_d(option="H")+
  geom_boxplot(width=0.1)+
  scale_fill_viridis_d(alpha=0.3,option="H")+
  labs(x = "PC2")
```

```
Warning: Groups with fewer than two datapoints have been dropped.
ℹ Set `drop = FALSE` to consider such groups for position adjustment purposes.
```

```
#Plot PC3
ggplot(PCA_face_neandertal_plotdf_2biv, aes(y = PC3,x=Species))+
  geom_violin(aes(color=Species, fill = Species))+
  scale_colour_viridis_d(option="H")+
  geom_boxplot(width=0.1)+
  scale_fill_viridis_d(alpha=0.3,option="H")+
  labs(x = "PC3")
```

```
Warning: Groups with fewer than two datapoints have been dropped.
ℹ Set `drop = FALSE` to consider such groups for position adjustment purposes.
```

```
#Plot PC4
ggplot(PCA_face_neandertal_plotdf_2biv, aes(y = PC4,x=Species))+
  geom_violin(aes(color=Species, fill = Species))+
  scale_colour_viridis_d(option="H")+
  geom_boxplot(width=0.1)+
  scale_fill_viridis_d(alpha=0.3,option="H")+
  labs(x = "PC4")
```

```
Warning: Groups with fewer than two datapoints have been dropped.
ℹ Set `drop = FALSE` to consider such groups for position adjustment purposes.
```

```
#Plot centroid size
ggplot(PCA_face_neandertal_plotdf_2biv, aes(y = Centroid_size, x=Species))+
  geom_violin(aes(color=Species, fill = Species))+
  scale_colour_viridis_d(option="H")+
  geom_boxplot(width=0.1)+
  scale_fill_viridis_d(alpha=0.3,option="H")+
  labs(x = "Centroid Size")
```

```
Warning: Groups with fewer than two datapoints have been dropped.
ℹ Set `drop = FALSE` to consider such groups for position adjustment purposes.
```

```
#3 Evolutionary models

#Create vector with species assignation
species_neandertal_2biv <- factor( face_data$SpeciesExpanded[neandertal_lineage_index_2biv]
)
                           
#load chronology data
chronology_neandertal_2biv <- taxa_chronology|>
  filter(
    !taxon %in% c(
      "early H. sapiens",
      "Upper Paleolithic H. sapiens",
      "H. sapiens"
      )
  )

#This function prepares the data to be passed to PaleoTS

####Geometric Mean IS RETURNING CALCULATION ERRORS IN THE EVOLUTIONARY MODEL TESTS, SO IT WAS REMOVED FROM THIS ANALYSIS.

PTS_PCS_neandertal_2biv <- dists2PaleoTS(data.frame(PCA_face_neandertal_plotdf_2biv[,c(1,3:6)]),species_neandertal_2biv,chronology_neandertal_2biv)
```

```
Warning in dists2PaleoTS(data.frame(PCA_face_neandertal_plotdf_2biv[, c(1, :
early Homo has N=1. Weigthed average variance used for it.
```

```
Warning in dists2PaleoTS(data.frame(PCA_face_neandertal_plotdf_2biv[, c(1, :
early Homo has N=1. Weigthed average variance used for it.
Warning in dists2PaleoTS(data.frame(PCA_face_neandertal_plotdf_2biv[, c(1, :
early Homo has N=1. Weigthed average variance used for it.
Warning in dists2PaleoTS(data.frame(PCA_face_neandertal_plotdf_2biv[, c(1, :
early Homo has N=1. Weigthed average variance used for it.
```

```
#This wrapper function will test the 6 evolutionary models considered in the article against each of the PCs and the centroid size.
PTS_PCS_models_neandertal_2biv <- multiple_paleoTS(PTS_PCS_neandertal_2biv,models=6)
```

```
Total # hypotheses:  2 
1  2  

Comparing 6 models [n = 5, method = Joint]

                  logL K     AICc     dAICc Akaike.wt
GRW          -7.909955 3 45.81991 24.545601     0.000
URW          -8.114694 2 26.22939  4.955079     0.068
Stasis       -7.560909 2 25.12182  3.847509     0.119
StrictStasis -8.970488 1 21.27431  0.000000     0.813
Punc-1       -6.640697 4      Inf       Inf     0.000
OU           -1.128282 4      Inf       Inf     0.000
```

```
Total # hypotheses:  2 
1  2  

Comparing 6 models [n = 5, method = Joint]

                    logL K     AICc     dAICc Akaike.wt
GRW           -0.4602687 3 30.92054 11.003918     0.003
URW           -4.9583096 2 19.91662  0.000000     0.841
Stasis        -6.6593399 2 23.31868  3.402061     0.153
StrictStasis -14.1548147 1 31.64296 11.726343     0.002
Punc-1        -1.8095586 4      Inf       Inf     0.000
OU            -0.4388617 4      Inf       Inf     0.000
```

```
Warning in compareModels(grw, urw, sta, strsta, pun2, ou): Optimization for the following model(s) did not converge: OU
These model fit(s) should not be considered reliable.
```

```
Total # hypotheses:  2 
1  2  

Comparing 6 models [n = 5, method = Joint]

                  logL K     AICc     dAICc Akaike.wt
GRW          -4.606814 3 39.21363 25.422721     0.000
URW          -5.251781 2 20.50356  6.712655     0.031
Stasis       -4.324582 2 18.64916  4.858258     0.078
StrictStasis -5.228787 1 13.79091  0.000000     0.890
Punc-1       -2.999390 4      Inf       Inf     0.000
OU           -4.049781 4      Inf       Inf     0.000
```

```
Total # hypotheses:  2 
1  2  

Comparing 6 models [n = 5, method = Joint]

                  logL K     AICc     dAICc Akaike.wt
GRW          -3.529365 3 37.05873 25.234802     0.000
URW          -4.273853 2 18.54771  6.723777     0.032
Stasis       -4.228005 2 18.45601  6.632081     0.034
StrictStasis -4.245298 1 11.82393  0.000000     0.934
Punc-1       -3.893203 4      Inf       Inf     0.000
OU           -3.044149 4      Inf       Inf     0.000
```

### 2c. early *Homo* only includes *H. rudolphensis* specimen

In this scenario, the *H. rudolphensis* specimen available for analysis (KNM ER 1470) is assumed to represent the average morphology of the early *Homo* OTU.

#### i. Neurocranial analyses of the *Homo sapiens* lineage

The same set of analyses in section 2b are replicated here, starting with the removal of the *Homo habilis* specimen, followed by PCA, and evolutionary models.

```
#1. Remove Homo habilis from dataset
sapiens_lineage_index_2ci <-  !neurocranium_data$SpeciesExpanded %in% c(
      "early H. neanderthalensis",
      "H. neanderthalensis"
      ) & neurocranium_data$Specimen != "KNM ER 1470"


neuro_sapiens_nomiss_2ci <- neuro_nomiss|>
  filter( #Filter out species not on H. sapiens lineage
    sapiens_lineage_index_2ci
  )

#2. Calculate PCA
PCA_neuro_sapiens_2ci <- PCA(neuro_sapiens_nomiss_2ci[,2:ncol(neuro_nomiss)],neuro_sapiens_nomiss_2ci[,1])

#A dataframe is created to facilitate ploting via ggplot
PCA_neuro_sapiens_plotdf_2ci <- tibble(
  Specimen = neuro_sapiens_nomiss_2ci$Specimen,
  Species =  factor(neurocranium_data$SpeciesExpanded[sapiens_lineage_index_2ci],
                    levels = 
                       c("early Homo",
                         "H. erectus",
                         "H. heidelbergensis s.l.", 
                         "early H. sapiens",
                         "Upper Paleolithic H. sapiens",
                         "H. sapiens")
  ),
  PC1 = PCA_neuro_sapiens_2ci$PCS[,1],
  PC2 = PCA_neuro_sapiens_2ci$PCS[,2],
  PC3 = PCA_neuro_sapiens_2ci$PCS[,3],
  PC4 = PCA_neuro_sapiens_2ci$PCS[,4],
  Centroid_size = neuro_gpa$Csize[sapiens_lineage_index_2ci]
)

#Plot PC1
ggplot(PCA_neuro_sapiens_plotdf_2ci, aes(y = PC1,x=Species))+
  geom_violin(aes(color=Species, fill = Species))+
  scale_colour_viridis_d(option="H")+
  geom_boxplot(width=0.1)+
  scale_fill_viridis_d(alpha=0.3,option="H")+
  labs(x = "PC1")
```

```
Warning: Groups with fewer than two datapoints have been dropped.
ℹ Set `drop = FALSE` to consider such groups for position adjustment purposes.
```

```
#Plot PC2
ggplot(PCA_neuro_sapiens_plotdf_2ci, aes(y = PC2,x=Species))+
  geom_violin(aes(color=Species, fill = Species))+
  scale_colour_viridis_d(option="H")+
  geom_boxplot(width=0.1)+
  scale_fill_viridis_d(alpha=0.3,option="H")+
  labs(x = "PC2")
```

```
Warning: Groups with fewer than two datapoints have been dropped.
ℹ Set `drop = FALSE` to consider such groups for position adjustment purposes.
```

```
#Plot PC3
ggplot(PCA_neuro_sapiens_plotdf_2ci, aes(y = PC3,x=Species))+
  geom_violin(aes(color=Species, fill = Species))+
  scale_colour_viridis_d(option="H")+
  geom_boxplot(width=0.1)+
  scale_fill_viridis_d(alpha=0.3,option="H")+
  labs(x = "PC3")
```

```
Warning: Groups with fewer than two datapoints have been dropped.
ℹ Set `drop = FALSE` to consider such groups for position adjustment purposes.
```

```
#Plot PC4
ggplot(PCA_neuro_sapiens_plotdf_2ci, aes(y = PC4,x=Species))+
  geom_violin(aes(color=Species, fill = Species))+
  scale_colour_viridis_d(option="H")+
  geom_boxplot(width=0.1)+
  scale_fill_viridis_d(alpha=0.3,option="H")+
  labs(x = "PC4")
```

```
Warning: Groups with fewer than two datapoints have been dropped.
ℹ Set `drop = FALSE` to consider such groups for position adjustment purposes.
```

```
#Plot centroid size
ggplot(PCA_neuro_sapiens_plotdf_2ci, aes(y = Centroid_size, x=Species))+
  geom_violin(aes(color=Species, fill = Species))+
  scale_colour_viridis_d(option="H")+
  geom_boxplot(width=0.1)+
  scale_fill_viridis_d(alpha=0.3,option="H")+
  labs(x = "Centroid Size")
```

```
Warning: Groups with fewer than two datapoints have been dropped.
ℹ Set `drop = FALSE` to consider such groups for position adjustment purposes.
```

```
#3 Evolutionary models

#Create vector with species assignation
species_sapiens_2ci <- factor( neurocranium_data$SpeciesExpanded[sapiens_lineage_index_2ci]
)
                           
#load chronology data
chronology_sapiens_2ci <- taxa_chronology|>
  filter(
    !taxon %in% c(
      "early H. neanderthalensis", 
      "H. neanderthalensis"
      )
  )

#This function prepares the data to be passed to PaleoTS
PTS_PCS_sapiens_2ci <- dists2PaleoTS(data.frame(PCA_neuro_sapiens_plotdf_2ci[,c(1,3:7)]),species_sapiens_2ci,chronology_sapiens_2ci)
```

```
Warning in dists2PaleoTS(data.frame(PCA_neuro_sapiens_plotdf_2ci[, c(1, : early
Homo has N=1. Weigthed average variance used for it.
```

```
Warning in dists2PaleoTS(data.frame(PCA_neuro_sapiens_plotdf_2ci[, c(1, : early
Homo has N=1. Weigthed average variance used for it.
Warning in dists2PaleoTS(data.frame(PCA_neuro_sapiens_plotdf_2ci[, c(1, : early
Homo has N=1. Weigthed average variance used for it.
Warning in dists2PaleoTS(data.frame(PCA_neuro_sapiens_plotdf_2ci[, c(1, : early
Homo has N=1. Weigthed average variance used for it.
Warning in dists2PaleoTS(data.frame(PCA_neuro_sapiens_plotdf_2ci[, c(1, : early
Homo has N=1. Weigthed average variance used for it.
```

```
#This wrapper function will test the 6 evolutionary models considered in the article against each of the PCs and the centroid size.
PTS_PCS_models_sapiens_2ci <- multiple_paleoTS(PTS_PCS_sapiens_2ci,models=6)
```

```
Total # hypotheses:  3 
1  2  3  

Comparing 6 models [n = 6, method = Joint]

                    logL K      AICc      dAICc Akaike.wt
GRW            -3.956252 3  25.91250   7.403152     0.023
URW            -5.254676 2  18.50935   0.000000     0.951
Stasis         -8.889958 2  25.77992   7.270565     0.025
StrictStasis -107.479576 1 217.95915 199.449800     0.000
Punc-1         -2.426927 4  52.85385  34.344502     0.000
OU             -3.959003 4  55.91801  37.408655     0.000
```

```
Warning in compareModels(grw, urw, sta, strsta, pun2, ou): Optimization for the following model(s) did not converge: OU
These model fit(s) should not be considered reliable.
```

```
Total # hypotheses:  3 
1  2  3  

Comparing 6 models [n = 6, method = Joint]

                  logL K     AICc     dAICc Akaike.wt
GRW          -4.973330 3 27.94666  9.381776     0.005
URW          -5.282442 2 18.56488  0.000000     0.547
Stasis       -6.485279 2 20.97056  2.405674     0.164
StrictStasis -8.439816 1 19.87963  1.314748     0.284
Punc-1       -2.885062 4 53.77012 35.205241     0.000
OU           -3.211468 4 54.42294 35.858051     0.000
```

```
Total # hypotheses:  3 
1  2  3  

Comparing 6 models [n = 6, method = Joint]

                  logL K     AICc     dAICc Akaike.wt
GRW          -6.230683 3 30.46137 15.035404     0.000
URW          -6.515412 2 21.03082  5.604862     0.056
Stasis       -3.712981 2 15.42596  0.000000     0.917
StrictStasis -9.753378 1 22.50676  7.080794     0.027
Punc-1       -2.422621 4 52.84524 37.419280     0.000
OU           -3.463899 4 54.92780 39.501835     0.000
```

```
Total # hypotheses:  3 
1  2  3  

Comparing 6 models [n = 6, method = Joint]

                  logL K      AICc     dAICc Akaike.wt
GRW          -3.010358 3 24.020716 15.090594     0.000
URW          -3.027310 2 14.054620  5.124498     0.065
Stasis       -2.741559 2 13.483119  4.552996     0.087
StrictStasis -2.965061 1  8.930122  0.000000     0.847
Punc-1       -2.125768 4 52.251536 43.321414     0.000
OU           -2.642529 4 53.285058 44.354936     0.000
```

```
Total # hypotheses:  3 
1  2  3  

Comparing 6 models [n = 6, method = Joint]

                   logL K     AICc     dAICc Akaike.wt
GRW          -13.662490 3 45.32498  9.631531     0.005
URW          -13.846724 2 35.69345  0.000000     0.639
Stasis       -14.433676 2 36.86735  1.173903     0.355
StrictStasis -30.546117 1 64.09223 28.398786     0.000
Punc-1       -11.131530 4 70.26306 34.569612     0.000
OU            -9.890604 4 67.78121 32.087759     0.000
```

#### ii. Neurocranial analysis of the *Homo neanderthalensis* lineage

This follows the same structure as the previous analysis.

```
#1. Remove Homo habilis from dataset
neandertal_lineage_index_2cii <- !neurocranium_data$SpeciesExpanded %in% c(
  "early H. sapiens",
  "Upper Paleolithic H. sapiens",
  "H. sapiens"
      ) & neurocranium_data$Specimen != "KNM ER 1470"

neuro_neandertal_nomiss_2cii <- neuro_nomiss|>
  filter( #Filter out species not on H. neanderthalensis lineage
    neandertal_lineage_index_2cii
  )

#2. Calculate PCA
PCA_neuro_neandertal_2cii <- PCA(neuro_neandertal_nomiss_2cii[,2:ncol(neuro_nomiss)],neuro_neandertal_nomiss_2cii[,1])

#A dataframe is created to facilitate ploting via ggplot
PCA_neuro_neandertal_plotdf_2cii <- tibble(
  Specimen = neuro_neandertal_nomiss_2cii$Specimen,
  Species =  factor(neurocranium_data$SpeciesExpanded[neandertal_lineage_index_2cii],
                    levels = 
                       c("early Homo",
                         "H. erectus",
                         "H. heidelbergensis s.l.", 
                         "early H. neanderthalensis",
                         "H. neanderthalensis")
  ),
  PC1 = PCA_neuro_neandertal_2cii$PCS[,1],
  PC2 = PCA_neuro_neandertal_2cii$PCS[,2],
  PC3 = PCA_neuro_neandertal_2cii$PCS[,3],
  PC4 = PCA_neuro_neandertal_2cii$PCS[,4],
  Centroid_size = neuro_gpa$Csize[neandertal_lineage_index_2cii]
)

#Plot PC1
ggplot(PCA_neuro_neandertal_plotdf_2cii, aes(y = PC1,x=Species))+
  geom_violin(aes(color=Species, fill = Species))+
  scale_colour_viridis_d(option="H")+
  geom_boxplot(width=0.1)+
  scale_fill_viridis_d(alpha=0.3,option="H")+
  labs(x = "PC1")
```

```
Warning: Groups with fewer than two datapoints have been dropped.
ℹ Set `drop = FALSE` to consider such groups for position adjustment purposes.
```

```
#Plot PC2
ggplot(PCA_neuro_neandertal_plotdf_2cii, aes(y = PC2,x=Species))+
  geom_violin(aes(color=Species, fill = Species))+
  scale_colour_viridis_d(option="H")+
  geom_boxplot(width=0.1)+
  scale_fill_viridis_d(alpha=0.3,option="H")+
  labs(x = "PC2")
```

```
Warning: Groups with fewer than two datapoints have been dropped.
ℹ Set `drop = FALSE` to consider such groups for position adjustment purposes.
```

```
#Plot PC3
ggplot(PCA_neuro_neandertal_plotdf_2cii, aes(y = PC3,x=Species))+
  geom_violin(aes(color=Species, fill = Species))+
  scale_colour_viridis_d(option="H")+
  geom_boxplot(width=0.1)+
  scale_fill_viridis_d(alpha=0.3,option="H")+
  labs(x = "PC3")
```

```
Warning: Groups with fewer than two datapoints have been dropped.
ℹ Set `drop = FALSE` to consider such groups for position adjustment purposes.
```

```
#Plot PC4
ggplot(PCA_neuro_neandertal_plotdf_2cii, aes(y = PC4,x=Species))+
  geom_violin(aes(color=Species, fill = Species))+
  scale_colour_viridis_d(option="H")+
  geom_boxplot(width=0.1)+
  scale_fill_viridis_d(alpha=0.3,option="H")+
  labs(x = "PC4")
```

```
Warning: Groups with fewer than two datapoints have been dropped.
ℹ Set `drop = FALSE` to consider such groups for position adjustment purposes.
```

```
#Plot centroid size
ggplot(PCA_neuro_neandertal_plotdf_2cii, aes(y = Centroid_size, x=Species))+
  geom_violin(aes(color=Species, fill = Species))+
  scale_colour_viridis_d(option="H")+
  geom_boxplot(width=0.1)+
  scale_fill_viridis_d(alpha=0.3,option="H")+
  labs(x = "Centroid Size")
```

```
Warning: Groups with fewer than two datapoints have been dropped.
ℹ Set `drop = FALSE` to consider such groups for position adjustment purposes.
```

```
#3 Evolutionary models

#Create vector with species assignation
species_neandertal_2cii <- factor( neurocranium_data$SpeciesExpanded[neandertal_lineage_index_2cii]
)
                           
#load chronology data
chronology_neandertal_2cii <- taxa_chronology|>
  filter(
    !taxon %in% c(
      "early H. sapiens",
      "Upper Paleolithic H. sapiens",
      "H. sapiens"
      )
  )

#This function prepares the data to be passed to PaleoTS
PTS_PCS_neandertal_2cii <- dists2PaleoTS(data.frame(PCA_neuro_neandertal_plotdf_2cii[,c(1,c(3,5:7))]),species_neandertal_2cii,chronology_neandertal_2cii)
```

```
Warning in dists2PaleoTS(data.frame(PCA_neuro_neandertal_plotdf_2cii[, c(1, :
early Homo has N=1. Weigthed average variance used for it.
```

```
Warning in dists2PaleoTS(data.frame(PCA_neuro_neandertal_plotdf_2cii[, c(1, :
early Homo has N=1. Weigthed average variance used for it.
Warning in dists2PaleoTS(data.frame(PCA_neuro_neandertal_plotdf_2cii[, c(1, :
early Homo has N=1. Weigthed average variance used for it.
Warning in dists2PaleoTS(data.frame(PCA_neuro_neandertal_plotdf_2cii[, c(1, :
early Homo has N=1. Weigthed average variance used for it.
```

```
#This wrapper function will test the 6 evolutionary models considered in the article against each of the PCs and the centroid size.
PTS_PCS_models_neandertal_2cii <- multiple_paleoTS(PTS_PCS_neandertal_2cii,models=6)
```

```
Total # hypotheses:  2 
1  2  

Comparing 6 models [n = 5, method = Joint]

                   logL K     AICc     dAICc Akaike.wt
GRW            1.452319 3 27.09536  8.873461     0.011
URW           -4.110951 2 18.22190  0.000000     0.934
Stasis        -6.944458 2 23.88892  5.667015     0.055
StrictStasis -38.230481 1 79.79429 61.572393     0.000
Punc-1        -1.674687 4      Inf       Inf     0.000
OU             1.475499 4      Inf       Inf     0.000
```

```
Warning in compareModels(grw, urw, sta, strsta, pun2, ou): Optimization for the following model(s) did not converge: OU
These model fit(s) should not be considered reliable.
```

```
Total # hypotheses:  2 
1  2  

Comparing 6 models [n = 5, method = Joint]

                  logL K     AICc     dAICc Akaike.wt
GRW          -7.642624 3 45.28525 26.509412     0.000
URW          -7.648394 2 25.29679  6.520952     0.023
Stasis       -4.907649 2 19.81530  1.039462     0.364
StrictStasis -7.721252 1 18.77584  0.000000     0.612
Punc-1       -4.341048 4      Inf       Inf     0.000
OU           -4.656548 4      Inf       Inf     0.000
```

```
Total # hypotheses:  2 
1  2  

Comparing 6 models [n = 5, method = Joint]

                   logL K      AICc     dAICc Akaike.wt
GRW          -1.1596539 3 32.319308 25.615654     0.000
URW          -1.7131858 2 13.426372  6.722717     0.032
Stasis       -1.6851743 2 13.370349  6.666694     0.033
StrictStasis -1.6851604 1  6.703654  0.000000     0.934
Punc-1       -0.9743775 4       Inf       Inf     0.000
OU           -1.0803625 4       Inf       Inf     0.000
```

```
Total # hypotheses:  2 
1  2  

Comparing 6 models [n = 5, method = Joint]

                   logL K     AICc     dAICc Akaike.wt
GRW           -8.276043 3 46.55209 14.856097     0.000
URW          -11.026499 2 32.05300  0.357008     0.386
Stasis       -11.956163 2 33.91233  2.216336     0.152
StrictStasis -14.181328 1 31.69599  0.000000     0.461
Punc-1        -8.246495 4      Inf       Inf     0.000
OU            -5.911439 4      Inf       Inf     0.000
```

#### iii. Facial analyses of the *Homo sapiens* lineage

This follows the same structure as the previous analysis.

```
#1. Remove Homo habilis from the dataset
sapiens_lineage_index_2ciii <- !face_data$SpeciesExpanded %in% c(
      "early H. neanderthalensis",
      "H. neanderthalensis"
      )& face_data$Specimen != "KNM ER 1470"

face_sapiens_nomiss_2ciii <- face_nomiss|>
  filter( #Filter out species not on H. sapiens lineage
    sapiens_lineage_index_2ciii
  )

#2. Calculate PCA
PCA_face_sapiens_2ciii <- PCA(face_sapiens_nomiss_2ciii[,2:ncol(face_nomiss)],face_sapiens_nomiss_2ciii[,1])

#A dataframe is created to facilitate ploting via ggplot
PCA_face_sapiens_plotdf_2ciii <- tibble(
  Specimen = face_sapiens_nomiss_2ciii$Specimen,
  Species =  factor(face_data$SpeciesExpanded[sapiens_lineage_index_2ciii],
                    levels = 
                       c("early Homo",
                         "H. erectus",
                         "H. heidelbergensis s.l.", 
                         "early H. sapiens",
                         "Upper Paleolithic H. sapiens",
                         "H. sapiens")
  ),
  PC1 = PCA_face_sapiens_2ciii$PCS[,1],
  PC2 = PCA_face_sapiens_2ciii$PCS[,2],
  PC3 = PCA_face_sapiens_2ciii$PCS[,3],
  PC4 = PCA_face_sapiens_2ciii$PCS[,4],
  Centroid_size = face_gpa$Csize[sapiens_lineage_index_2ciii]
)

#Plot PC1
ggplot(PCA_face_sapiens_plotdf_2ciii, aes(y = PC1,x=Species))+
  geom_violin(aes(color=Species, fill = Species))+
  scale_colour_viridis_d(option="H")+
  geom_boxplot(width=0.1)+
  scale_fill_viridis_d(alpha=0.3,option="H")+
  labs(x = "PC1")
```

```
Warning: Groups with fewer than two datapoints have been dropped.
ℹ Set `drop = FALSE` to consider such groups for position adjustment purposes.
```

```
#Plot PC2
ggplot(PCA_face_sapiens_plotdf_2ciii, aes(y = PC2,x=Species))+
  geom_violin(aes(color=Species, fill = Species))+
  scale_colour_viridis_d(option="H")+
  geom_boxplot(width=0.1)+
  scale_fill_viridis_d(alpha=0.3,option="H")+
  labs(x = "PC2")
```

```
Warning: Groups with fewer than two datapoints have been dropped.
ℹ Set `drop = FALSE` to consider such groups for position adjustment purposes.
```

```
#Plot PC3
ggplot(PCA_face_sapiens_plotdf_2ciii, aes(y = PC3,x=Species))+
  geom_violin(aes(color=Species, fill = Species))+
  scale_colour_viridis_d(option="H")+
  geom_boxplot(width=0.1)+
  scale_fill_viridis_d(alpha=0.3,option="H")+
  labs(x = "PC3")
```

```
Warning: Groups with fewer than two datapoints have been dropped.
ℹ Set `drop = FALSE` to consider such groups for position adjustment purposes.
```

```
#Plot PC4
ggplot(PCA_face_sapiens_plotdf_2ciii, aes(y = PC4,x=Species))+
  geom_violin(aes(color=Species, fill = Species))+
  scale_colour_viridis_d(option="H")+
  geom_boxplot(width=0.1)+
  scale_fill_viridis_d(alpha=0.3,option="H")+
  labs(x = "PC4")
```

```
Warning: Groups with fewer than two datapoints have been dropped.
ℹ Set `drop = FALSE` to consider such groups for position adjustment purposes.
```

```
#Plot centroid size
ggplot(PCA_face_sapiens_plotdf_2ciii, aes(y = Centroid_size, x=Species))+
  geom_violin(aes(color=Species, fill = Species))+
  scale_colour_viridis_d(option="H")+
  geom_boxplot(width=0.1)+
  scale_fill_viridis_d(alpha=0.3,option="H")+
  labs(x = "Centroid Size")
```

```
Warning: Groups with fewer than two datapoints have been dropped.
ℹ Set `drop = FALSE` to consider such groups for position adjustment purposes.
```

```
#3 Evolutionary models

#Create vector with species assignation
species_sapiens_2ciii <- factor( face_data$SpeciesExpanded[sapiens_lineage_index_2ciii]
)
                           
#load chronology data
chronology_sapiens_2ciii <- taxa_chronology|>
  filter(
    !taxon %in% c(
      "early H. neanderthalensis", 
      "H. neanderthalensis"
      )
  )

#This function prepares the data to be passed to PaleoTS
PTS_PCS_sapiens_2ciii <- dists2PaleoTS(data.frame(PCA_face_sapiens_plotdf_2ciii[,c(1,3:7)]),species_sapiens_2ciii,chronology_sapiens_2ciii)
```

```
Warning in dists2PaleoTS(data.frame(PCA_face_sapiens_plotdf_2ciii[, c(1, :
early Homo has N=1. Weigthed average variance used for it.
```

```
Warning in dists2PaleoTS(data.frame(PCA_face_sapiens_plotdf_2ciii[, c(1, :
early Homo has N=1. Weigthed average variance used for it.
Warning in dists2PaleoTS(data.frame(PCA_face_sapiens_plotdf_2ciii[, c(1, :
early Homo has N=1. Weigthed average variance used for it.
Warning in dists2PaleoTS(data.frame(PCA_face_sapiens_plotdf_2ciii[, c(1, :
early Homo has N=1. Weigthed average variance used for it.
Warning in dists2PaleoTS(data.frame(PCA_face_sapiens_plotdf_2ciii[, c(1, :
early Homo has N=1. Weigthed average variance used for it.
```

```
#This wrapper function will  test the 6 evolutionary models considered in the article against each of the PCs and the centroid size. 
PTS_PCS_models_sapiens_2ciii <- multiple_paleoTS(PTS_PCS_sapiens_2ciii,models=6)
```

```
Total # hypotheses:  3 
1  2  3  

Comparing 6 models [n = 6, method = Joint]

                   logL K     AICc      dAICc Akaike.wt
GRW           -5.307035 3 28.61407  7.0423384     0.018
URW           -7.249790 2 22.49958  0.9278492     0.379
Stasis        -6.785865 2 21.57173  0.0000000     0.603
StrictStasis -20.162984 1 43.32597 21.7542378     0.000
Punc-1        -2.932041 4 53.86408 32.2923521     0.000
OU            -6.059047 4 60.11809 38.5463628     0.000
```

```
Total # hypotheses:  3 
1  2  3  

Comparing 6 models [n = 6, method = Joint]

                  logL K     AICc     dAICc Akaike.wt
GRW          -3.847354 3 25.69471 14.774285     0.001
URW          -4.041481 2 16.08296  5.162538     0.065
Stasis       -3.960220 2 15.92044  5.000016     0.071
StrictStasis -3.960212 1 10.92042  0.000000     0.863
Punc-1       -3.417165 4 54.83433 43.913906     0.000
OU           -3.713688 4 55.42738 44.506952     0.000
```

```
Total # hypotheses:  3 
1  2  3  

Comparing 6 models [n = 6, method = Joint]

                   logL K     AICc     dAICc Akaike.wt
GRW           -7.444195 3 32.88839 10.671026     0.003
URW           -7.824068 2 23.64814  1.430771     0.327
Stasis        -7.108682 2 22.21736  0.000000     0.669
StrictStasis -48.261615 1 99.52323 77.305866     0.000
Punc-1        -2.568617 4 53.13723 30.919869     0.000
OU            -5.901136 4 59.80227 37.584907     0.000
```

```
Total # hypotheses:  3 
1  2  3  

Comparing 6 models [n = 6, method = Joint]

                   logL K     AICc     dAICc Akaike.wt
GRW           -6.830637 3 31.66127  9.557436     0.006
URW           -7.051919 2 22.10384  0.000000     0.670
Stasis        -7.921500 2 23.84300  1.739162     0.281
StrictStasis -12.301901 1 27.60380  5.499965     0.043
Punc-1        -5.367229 4 58.73446 36.630621     0.000
OU            -5.326131 4 58.65226 36.548425     0.000
```

```
Total # hypotheses:  3 
1  2  3  

Comparing 6 models [n = 6, method = Joint]

                  logL K      AICc      dAICc Akaike.wt
GRW          -12.68625 3  43.37250  9.9994081     0.004
URW          -12.68654 2  33.37309  0.0000000     0.574
Stasis       -12.99255 2  33.98510  0.6120129     0.422
StrictStasis -52.76026 1 108.52051 75.1474253     0.000
Punc-1       -11.32350 4  70.64699 37.2739036     0.000
OU           -10.78614 4  69.57229 36.1991971     0.000
```

#### iv. Facial analysis of the *Homo neanderthalensis* lineage

This follows the same structure as the previous analysis.

```
#1. Remove Homo habilis from the dataset
neandertal_lineage_index_2civ <- !face_data$SpeciesExpanded %in% c(
  "early H. sapiens",
  "Upper Paleolithic H. sapiens",
  "H. sapiens"
      )& face_data$Specimen != "KNM ER 1470"

face_neandertal_nomiss_2civ <- face_nomiss|>
  filter( #Filter out species not on H. neanderthalensis lineage
    neandertal_lineage_index_2civ
  )

#2. Calculate PCA
PCA_face_neandertal_2civ <- PCA(face_neandertal_nomiss_2civ[,2:ncol(face_nomiss)],face_neandertal_nomiss_2civ[,1])

#A dataframe is created to facilitate ploting via ggplot
PCA_face_neandertal_plotdf_2civ <- tibble(
  Specimen = face_neandertal_nomiss_2civ$Specimen,
  Species =  factor(face_data$SpeciesExpanded[neandertal_lineage_index_2civ],
                    levels = 
                       c("early Homo",
                         "H. erectus",
                         "H. heidelbergensis s.l.", 
                         "early H. neanderthalensis",
                         "H. neanderthalensis")
  ),
  PC1 = PCA_face_neandertal_2civ$PCS[,1],
  PC2 = PCA_face_neandertal_2civ$PCS[,2],
  PC3 = PCA_face_neandertal_2civ$PCS[,3],
  PC4 = PCA_face_neandertal_2civ$PCS[,4],
  Centroid_size = face_gpa$Csize[neandertal_lineage_index_2civ]
)

#Plot PC1
ggplot(PCA_face_neandertal_plotdf_2civ, aes(y = PC1,x=Species))+
  geom_violin(aes(color=Species, fill = Species))+
  scale_colour_viridis_d(option="H")+
  geom_boxplot(width=0.1)+
  scale_fill_viridis_d(alpha=0.3,option="H")+
  labs(x = "PC1")
```

```
Warning: Groups with fewer than two datapoints have been dropped.
ℹ Set `drop = FALSE` to consider such groups for position adjustment purposes.
```

```
#Plot PC2
ggplot(PCA_face_neandertal_plotdf_2civ, aes(y = PC2,x=Species))+
  geom_violin(aes(color=Species, fill = Species))+
  scale_colour_viridis_d(option="H")+
  geom_boxplot(width=0.1)+
  scale_fill_viridis_d(alpha=0.3,option="H")+
  labs(x = "PC2")
```

```
Warning: Groups with fewer than two datapoints have been dropped.
ℹ Set `drop = FALSE` to consider such groups for position adjustment purposes.
```

```
#Plot PC3
ggplot(PCA_face_neandertal_plotdf_2civ, aes(y = PC3,x=Species))+
  geom_violin(aes(color=Species, fill = Species))+
  scale_colour_viridis_d(option="H")+
  geom_boxplot(width=0.1)+
  scale_fill_viridis_d(alpha=0.3,option="H")+
  labs(x = "PC3")
```

```
Warning: Groups with fewer than two datapoints have been dropped.
ℹ Set `drop = FALSE` to consider such groups for position adjustment purposes.
```

```
#Plot PC4
ggplot(PCA_face_neandertal_plotdf_2civ, aes(y = PC4,x=Species))+
  geom_violin(aes(color=Species, fill = Species))+
  scale_colour_viridis_d(option="H")+
  geom_boxplot(width=0.1)+
  scale_fill_viridis_d(alpha=0.3,option="H")+
  labs(x = "PC4")
```

```
Warning: Groups with fewer than two datapoints have been dropped.
ℹ Set `drop = FALSE` to consider such groups for position adjustment purposes.
```

```
#Plot centroid size
ggplot(PCA_face_neandertal_plotdf_2civ, aes(y = Centroid_size, x=Species))+
  geom_violin(aes(color=Species, fill = Species))+
  scale_colour_viridis_d(option="H")+
  geom_boxplot(width=0.1)+
  scale_fill_viridis_d(alpha=0.3,option="H")+
  labs(x = "Centroid Size")
```

```
Warning: Groups with fewer than two datapoints have been dropped.
ℹ Set `drop = FALSE` to consider such groups for position adjustment purposes.
```

```
#3 Evolutionary models

#Create vector with species assignation
species_neandertal_2civ <- factor( face_data$SpeciesExpanded[neandertal_lineage_index_2civ]
)
                           
#load chronology data
chronology_neandertal_2civ <- taxa_chronology|>
  filter(
    !taxon %in% c(
      "early H. sapiens",
      "Upper Paleolithic H. sapiens",
      "H. sapiens"
      )
  )

#This function prepares the data to be passed to PaleoTS

####Geometric Mean IS RETURNING CALCULATION ERRORS IN THE EVOLUTIONARY MODEL TESTS, SO IT WAS REMOVED FROM THIS ANALYSIS.

PTS_PCS_neandertal_2civ <- dists2PaleoTS(data.frame(PCA_face_neandertal_plotdf_2civ[,c(1,3:5)]),species_neandertal_2civ,chronology_neandertal_2civ)
```

```
Warning in dists2PaleoTS(data.frame(PCA_face_neandertal_plotdf_2civ[, c(1, :
early Homo has N=1. Weigthed average variance used for it.
```

```
Warning in dists2PaleoTS(data.frame(PCA_face_neandertal_plotdf_2civ[, c(1, :
early Homo has N=1. Weigthed average variance used for it.
Warning in dists2PaleoTS(data.frame(PCA_face_neandertal_plotdf_2civ[, c(1, :
early Homo has N=1. Weigthed average variance used for it.
```

```
#This wrapper function will test the 6 evolutionary models considered in the article against each of the PCs and the centroid size. 
PTS_PCS_models_neandertal_2civ <- multiple_paleoTS(PTS_PCS_neandertal_2civ,models=6)
```

```
Total # hypotheses:  2 
1  2  

Comparing 6 models [n = 5, method = Joint]

                  logL K     AICc     dAICc Akaike.wt
GRW          -2.153486 3 34.30697 16.152946     0.000
URW          -5.486027 2 20.97205  2.818029     0.173
Stasis       -5.845116 2 21.69023  3.536208     0.121
StrictStasis -7.410346 1 18.15402  0.000000     0.707
Punc-1       -2.874586 4      Inf       Inf     0.000
OU           -2.129966 4      Inf       Inf     0.000
```

```
Warning in compareModels(grw, urw, sta, strsta, pun2, ou): Optimization for the following model(s) did not converge: OU
These model fit(s) should not be considered reliable.
```

```
Total # hypotheses:  2 
1  2  

Comparing 6 models [n = 5, method = Joint]

                  logL K     AICc     dAICc Akaike.wt
GRW          -3.549893 3 37.09979 19.573584     0.000
URW          -5.299083 2 20.59817  3.071965     0.158
Stasis       -5.668429 2 21.33686  3.810656     0.109
StrictStasis -7.096434 1 17.52620  0.000000     0.733
Punc-1       -1.402997 4      Inf       Inf     0.000
OU           -2.831789 4      Inf       Inf     0.000
```

```
Total # hypotheses:  2 
1  2  

Comparing 6 models [n = 5, method = Joint]

                  logL K     AICc     dAICc Akaike.wt
GRW          -3.325862 3 36.65172 26.654625     0.000
URW          -3.348750 2 16.69750  6.700400     0.033
Stasis       -3.331887 2 16.66377  6.666674     0.033
StrictStasis -3.331883 1  9.99710  0.000000     0.934
Punc-1       -3.022851 4      Inf       Inf     0.000
OU           -2.599629 4      Inf       Inf     0.000
```

### 2d. *Homo sapiens* lineages excluding modern *Homo sapiens*

Given the recent morphological changes experienced by *Homo sapiens* in the Holocene, this section presents the evolutionary model results for the *Homo sapiens* lineages that do not include this most recent OTU. This test also makes the analysis more comparable to the *Homo neanderthalensis* lineage as it presents similar OTU numbers and sample sizes.

#### i. Neurocranial analyses of the *Homo sapiens* lineage

The same set of analyses done in section 2a are replicated here, starting with the removal of modern *Homo sapiens*, followed by PCA, and evolutionary models.

```
#1. Remove recent Homo sapiens from dataset
sapiens_lineage_index_2di <- !neurocranium_data$SpeciesExpanded %in% c(
      "early H. neanderthalensis",
      "H. neanderthalensis",
      "H. sapiens"
      )

neuro_sapiens_nomiss_2di <- neuro_nomiss|>
  filter( #Filter out species not on H. sapiens lineage
    sapiens_lineage_index_2di
  )

#2. Calculate PCA
PCA_neuro_sapiens_2di <- PCA(neuro_sapiens_nomiss_2di[,2:ncol(neuro_nomiss)],neuro_sapiens_nomiss_2di[,1])

#A dataframe is created to facilitate ploting via ggplot
PCA_neuro_sapiens_plotdf_2di <- tibble(
  Specimen = neuro_sapiens_nomiss_2di$Specimen,
  Species =  factor(neurocranium_data$SpeciesExpanded[sapiens_lineage_index_2di],
                    levels = 
                       c("early Homo",
                         "H. erectus",
                         "H. heidelbergensis s.l.", 
                         "early H. sapiens",
                         "Upper Paleolithic H. sapiens"
                         )
  ),
  PC1 = PCA_neuro_sapiens_2di$PCS[,1],
  PC2 = PCA_neuro_sapiens_2di$PCS[,2],
  PC3 = PCA_neuro_sapiens_2di$PCS[,3],
  PC4 = PCA_neuro_sapiens_2di$PCS[,4],
  Centroid_size = neuro_gpa$Csize[sapiens_lineage_index_2di]
)

#Plot PC1
ggplot(PCA_neuro_sapiens_plotdf_2di, aes(y = PC1,x=Species))+
  geom_violin(aes(color=Species, fill = Species))+
  scale_colour_viridis_d(option="H")+
  geom_boxplot(width=0.1)+
  scale_fill_viridis_d(alpha=0.3,option="H")+
  labs(x = "PC1")
```

```
#Plot PC2
ggplot(PCA_neuro_sapiens_plotdf_2di, aes(y = PC2,x=Species))+
  geom_violin(aes(color=Species, fill = Species))+
  scale_colour_viridis_d(option="H")+
  geom_boxplot(width=0.1)+
  scale_fill_viridis_d(alpha=0.3,option="H")+
  labs(x = "PC2")
```

```
#Plot PC3
ggplot(PCA_neuro_sapiens_plotdf_2di, aes(y = PC3,x=Species))+
  geom_violin(aes(color=Species, fill = Species))+
  scale_colour_viridis_d(option="H")+
  geom_boxplot(width=0.1)+
  scale_fill_viridis_d(alpha=0.3,option="H")+
  labs(x = "PC3")
```

```
#Plot PC4
ggplot(PCA_neuro_sapiens_plotdf_2di, aes(y = PC4,x=Species))+
  geom_violin(aes(color=Species, fill = Species))+
  scale_colour_viridis_d(option="H")+
  geom_boxplot(width=0.1)+
  scale_fill_viridis_d(alpha=0.3,option="H")+
  labs(x = "PC4")
```

```
#Plot centroid size
ggplot(PCA_neuro_sapiens_plotdf_2di, aes(y = Centroid_size, x=Species))+
  geom_violin(aes(color=Species, fill = Species))+
  scale_colour_viridis_d(option="H")+
  geom_boxplot(width=0.1)+
  scale_fill_viridis_d(alpha=0.3,option="H")+
  labs(x = "Centroid Size")
```

```
#3 Evolutionary models

#Create vector with species assignation
species_sapiens_2di <- factor( neurocranium_data$SpeciesExpanded[sapiens_lineage_index_2di]
)
                           
#load chronology data
chronology_sapiens_2di <- taxa_chronology|>
  filter(
    !taxon %in% c(
      "early H. neanderthalensis", 
      "H. neanderthalensis",
      "H. sapiens"
      )
  )

#This function prepares the data to be passed to PaleoTS
PTS_PCS_sapiens_2di <- dists2PaleoTS(data.frame(PCA_neuro_sapiens_plotdf_2di[,c(1,3:7)]),species_sapiens_2di,chronology_sapiens_2di)

#This wrapper function will run test the 6 evolutionary models considered in the article against each of the PCs and the centroid size.
PTS_PCS_models_sapiens_2di <- multiple_paleoTS(PTS_PCS_sapiens_2di,models=6)
```

```
Total # hypotheses:  2 
1  2  

Comparing 6 models [n = 5, method = Joint]

                    logL K      AICc      dAICc Akaike.wt
GRW            -1.497780 3  32.99556  16.581214     0.000
URW            -3.207173 2  16.41435   0.000000     0.974
Stasis         -6.821722 2  23.64344   7.229098     0.026
StrictStasis -140.070668 1 283.47467 267.060322     0.000
Punc-1         -2.998032 4       Inf        Inf     0.000
OU             -1.505890 4       Inf        Inf     0.000
```

```
Warning in compareModels(grw, urw, sta, strsta, pun2, ou): Optimization for the following model(s) did not converge: OU
These model fit(s) should not be considered reliable.
```

```
Total # hypotheses:  2 
1  2  

Comparing 6 models [n = 5, method = Joint]

                  logL K     AICc     dAICc Akaike.wt
GRW          -5.724440 3 41.44888 22.747511     0.000
URW          -5.814289 2 21.62858  2.927208     0.150
Stasis       -5.523370 2 21.04674  2.345369     0.201
StrictStasis -7.684018 1 18.70137  0.000000     0.649
Punc-1       -4.236991 4      Inf       Inf     0.000
OU           -3.604746 4      Inf       Inf     0.000
```

```
Total # hypotheses:  2 
1  2  

Comparing 6 models [n = 5, method = Joint]

                   logL K      AICc     dAICc Akaike.wt
GRW          -2.1321862 3 34.264372 25.256575     0.000
URW          -2.9841799 2 15.968360  6.960562     0.028
Stasis       -2.2780515 2 14.556103  5.548305     0.057
StrictStasis -2.8372321 1  9.007798  0.000000     0.915
Punc-1        0.2834333 4       Inf       Inf     0.000
OU           -1.8327636 4       Inf       Inf     0.000
```

```
Total # hypotheses:  2 
1  2  

Comparing 6 models [n = 5, method = Joint]

                   logL K      AICc     dAICc Akaike.wt
GRW           0.6718661 3 28.656268 21.907794     0.000
URW          -1.5506582 2 13.101316  6.352843     0.039
Stasis       -1.5501582 2 13.100316  6.351843     0.039
StrictStasis -1.7075703 1  6.748474  0.000000     0.923
Punc-1        0.9832658 4       Inf       Inf     0.000
OU            1.3455447 4       Inf       Inf     0.000
```

```
Total # hypotheses:  2 
1  2  

Comparing 6 models [n = 5, method = Joint]

                   logL K     AICc     dAICc Akaike.wt
GRW           -6.452939 3 42.90588 19.036938     0.000
URW           -9.163202 2 28.32640  4.457463     0.094
Stasis       -10.145312 2 30.29062  6.421683     0.035
StrictStasis -10.267804 1 23.86894  0.000000     0.871
Punc-1        -5.447168 4      Inf       Inf     0.000
OU            -5.030212 4      Inf       Inf     0.000
```

#### ii. Facial analyses of the *Homo sapiens* lineage

This follows the same structure as the previous analysis, now for the facial dataset.

```
#1. Remove recent Homo sapiens from dataset
sapiens_lineage_index_2dii <- !face_data$SpeciesExpanded %in% c(
      "early H. neanderthalensis",
      "H. neanderthalensis",
      "H. sapiens"
      )

face_sapiens_nomiss_2dii <- face_nomiss|>
  filter( #Filter out species not on H. sapiens lineage
    sapiens_lineage_index_2dii
  )

#2. Calculate PCA
PCA_face_sapiens_2dii <- PCA(face_sapiens_nomiss_2dii[,2:ncol(face_nomiss)],face_sapiens_nomiss_2dii[,1])

#A dataframe is created to facilitate ploting via ggplot
PCA_face_sapiens_plotdf_2dii <- tibble(
  Specimen = face_sapiens_nomiss_2dii$Specimen,
  Species =  factor(face_data$SpeciesExpanded[sapiens_lineage_index_2dii],
                    levels = 
                       c("early Homo",
                         "H. erectus",
                         "H. heidelbergensis s.l.", 
                         "early H. sapiens",
                         "Upper Paleolithic H. sapiens")
  ),
  PC1 = PCA_face_sapiens_2dii$PCS[,1],
  PC2 = PCA_face_sapiens_2dii$PCS[,2],
  PC3 = PCA_face_sapiens_2dii$PCS[,3],
  PC4 = PCA_face_sapiens_2dii$PCS[,4],
  Centroid_size = face_gpa$Csize[sapiens_lineage_index_2dii]
)

#Plot PC1
ggplot(PCA_face_sapiens_plotdf_2dii, aes(y = PC1,x=Species))+
  geom_violin(aes(color=Species, fill = Species))+
  scale_colour_viridis_d(option="H")+
  geom_boxplot(width=0.1)+
  scale_fill_viridis_d(alpha=0.3,option="H")+
  labs(x = "PC1")
```

```
#Plot PC2
ggplot(PCA_face_sapiens_plotdf_2dii, aes(y = PC2,x=Species))+
  geom_violin(aes(color=Species, fill = Species))+
  scale_colour_viridis_d(option="H")+
  geom_boxplot(width=0.1)+
  scale_fill_viridis_d(alpha=0.3,option="H")+
  labs(x = "PC2")
```

```
#Plot PC3
ggplot(PCA_face_sapiens_plotdf_2dii, aes(y = PC3,x=Species))+
  geom_violin(aes(color=Species, fill = Species))+
  scale_colour_viridis_d(option="H")+
  geom_boxplot(width=0.1)+
  scale_fill_viridis_d(alpha=0.3,option="H")+
  labs(x = "PC3")
```

```
#Plot PC4
ggplot(PCA_face_sapiens_plotdf_2dii, aes(y = PC4,x=Species))+
  geom_violin(aes(color=Species, fill = Species))+
  scale_colour_viridis_d(option="H")+
  geom_boxplot(width=0.1)+
  scale_fill_viridis_d(alpha=0.3,option="H")+
  labs(x = "PC4")
```

```
#Plot centroid size
ggplot(PCA_face_sapiens_plotdf_2dii, aes(y = Centroid_size, x=Species))+
  geom_violin(aes(color=Species, fill = Species))+
  scale_colour_viridis_d(option="H")+
  geom_boxplot(width=0.1)+
  scale_fill_viridis_d(alpha=0.3,option="H")+
  labs(x = "Centroid Size")
```

```
#3 Evolutionary models

#Create vector with species assignation
species_sapiens_2dii <- factor( face_data$SpeciesExpanded[sapiens_lineage_index_2dii]
)
                           
#load chronology data
chronology_sapiens_2dii <- taxa_chronology|>
  filter(
    !taxon %in% c(
      "early H. neanderthalensis", 
      "H. neanderthalensis",
      "H. sapiens"
      )
  )

#This function prepares the data to be passed to PaleoTS
PTS_PCS_sapiens_2dii <- dists2PaleoTS(data.frame(PCA_face_sapiens_plotdf_2dii[,c(1,3:7)]),species_sapiens_2dii,chronology_sapiens_2dii)

#This wrapper function will run test the 6 evolutionary models considered in the article against each of the PCs and the centroid size.
PTS_PCS_models_sapiens_2dii <- multiple_paleoTS(PTS_PCS_sapiens_2dii,models=6)
```

```
Total # hypotheses:  2 
1  2  

Comparing 6 models [n = 5, method = Joint]

                   logL K     AICc     dAICc Akaike.wt
GRW           -2.959613 3 35.91923 16.378021     0.000
URW           -4.770602 2 19.54120  0.000000     0.747
Stasis        -5.851773 2 21.70355  2.162341     0.253
StrictStasis -17.956479 1 39.24629 19.705088     0.000
Punc-1        -1.108530 4      Inf       Inf     0.000
OU            -3.226821 4      Inf       Inf     0.000
```

```
Warning in compareModels(grw, urw, sta, strsta, pun2, ou): Optimization for the following model(s) did not converge: OU
These model fit(s) should not be considered reliable.
```

```
Total # hypotheses:  2 
1  2  

Comparing 6 models [n = 5, method = Joint]

                  logL K     AICc     dAICc Akaike.wt
GRW          -4.574922 3 39.14984 21.852893     0.000
URW          -4.752275 2 19.50455  2.207599     0.197
Stasis       -3.648476 2 17.29695  0.000000     0.593
StrictStasis -8.020156 1 19.37364  2.076693     0.210
Punc-1       -3.110795 4      Inf       Inf     0.000
OU           -2.160141 4      Inf       Inf     0.000
```

```
Total # hypotheses:  2 
1  2  

Comparing 6 models [n = 5, method = Joint]

                  logL K     AICc      dAICc Akaike.wt
GRW          -2.146990 3 34.29398 14.8367421     0.000
URW          -4.987786 2 19.97557  0.5183339     0.291
Stasis       -4.855965 2 19.71193  0.2546920     0.332
StrictStasis -8.061952 1 19.45724  0.0000000     0.377
Punc-1       -1.623249 4      Inf        Inf     0.000
OU           -2.130029 4      Inf        Inf     0.000
```

```
Warning in compareModels(grw, urw, sta, strsta, pun2, ou): Optimization for the following model(s) did not converge: OU
These model fit(s) should not be considered reliable.
```

```
Total # hypotheses:  2 
1  2  

Comparing 6 models [n = 5, method = Joint]

                  logL K     AICc     dAICc Akaike.wt
GRW          -5.349643 3 40.69929 20.956129     0.000
URW          -5.545276 2 21.09055  1.347395     0.257
Stasis       -5.622301 2 21.24460  1.501445     0.238
StrictStasis -8.204912 1 19.74316  0.000000     0.505
Punc-1       -3.217994 4      Inf       Inf     0.000
OU           -3.854503 4      Inf       Inf     0.000
```

```
Total # hypotheses:  2 
1  2  

Comparing 6 models [n = 5, method = Joint]

                   logL K     AICc     dAICc Akaike.wt
GRW          -11.457628 3 52.91526 23.040340     0.000
URW          -11.483332 2 32.96666  3.091748     0.176
Stasis        -9.937458 2 29.87492  0.000000     0.824
StrictStasis -34.929881 1 73.19310 43.318179     0.000
Punc-1        -8.941715 4      Inf       Inf     0.000
OU            -9.531685 4      Inf       Inf     0.000
```

### 2e. *Homo neanderthalensis* lineages joining all *H. neanderthalensis* in one OTU

The final alternative scenario explores the the fit of evolutionary models to the *H. neaderthalensis* lineage when early and late *H. neaderthalensis* specimens are grouped in a single OTU. This analysis aims to explore the impact fo considering *H. neaderthalensis* as two difference OTUs in the analyses presented in the article, which uses OTUs that create divisions along different accepted taxonomic levels.

#### i. Neurocranial analyses of the *Homo neanderthalensis* lineage

The analysis follows the same structure as the previous section 2d in the supplementary section.

```
#1. Remove specimens that are nots from the H. neanderthalensis lineage
neandertal_lineage_index_2ei <- !neurocranium_data$SpeciesExpanded %in% c(
  "early H. sapiens",
  "Upper Paleolithic H. sapiens",
  "H. sapiens"
)

neuro_neandertal_nomiss_2ei <- neuro_nomiss|>
  filter( #Filter out species not on H. neanderthalensis lineage
    neandertal_lineage_index_2ei
  )

#2. Calculate PCA
PCA_neuro_neandertal_2ei <- PCA(neuro_neandertal_nomiss_2ei[,2:ncol(neuro_nomiss)],neuro_neandertal_nomiss_2ei[,1])

#A dataframe is created to facilitate ploting via ggplot
PCA_neuro_neandertal_plotdf_2ei <- tibble(
  Specimen = neuro_neandertal_nomiss_2ei$Specimen,
  Species =  neurocranium_data$SpeciesExpanded[neandertal_lineage_index_2ei],
  PC1 = PCA_neuro_neandertal_2ei$PCS[,1],
  PC2 = PCA_neuro_neandertal_2ei$PCS[,2],
  PC3 = PCA_neuro_neandertal_2ei$PCS[,3],
  PC4 = PCA_neuro_neandertal_2ei$PCS[,4],
  Centroid_size = neuro_gpa$Csize[neandertal_lineage_index_2ei]
)

#Group H. neanderthalensis specimens in one OTU.
PCA_neuro_neandertal_plotdf_2ei <- PCA_neuro_neandertal_plotdf_2ei|>
  mutate(
    Species = ifelse(Species == "early H. neanderthalensis", 
                     "H. neanderthalensis",
                     Species),
    Species = factor(Species, levels = c(
      "early Homo",
      "H. erectus",
      "H. heidelbergensis s.l.",
      "H. neanderthalensis"
    )
    )
  
)

#Plot PC1
ggplot(PCA_neuro_neandertal_plotdf_2ei, aes(y = PC1,x=Species))+
  geom_violin(aes(color=Species, fill = Species))+
  scale_colour_viridis_d(option="H")+
  geom_boxplot(width=0.1)+
  scale_fill_viridis_d(alpha=0.3,option="H")+
  labs(x = "PC1")
```

```
#Plot PC2
ggplot(PCA_neuro_neandertal_plotdf_2ei, aes(y = PC2,x=Species))+
  geom_violin(aes(color=Species, fill = Species))+
  scale_colour_viridis_d(option="H")+
  geom_boxplot(width=0.1)+
  scale_fill_viridis_d(alpha=0.3,option="H")+
  labs(x = "PC2")
```

```
#Plot PC3
ggplot(PCA_neuro_neandertal_plotdf_2ei, aes(y = PC3,x=Species))+
  geom_violin(aes(color=Species, fill = Species))+
  scale_colour_viridis_d(option="H")+
  geom_boxplot(width=0.1)+
  scale_fill_viridis_d(alpha=0.3,option="H")+
  labs(x = "PC3")
```

```
#Plot PC4
ggplot(PCA_neuro_neandertal_plotdf_2ei, aes(y = PC4,x=Species))+
  geom_violin(aes(color=Species, fill = Species))+
  scale_colour_viridis_d(option="H")+
  geom_boxplot(width=0.1)+
  scale_fill_viridis_d(alpha=0.3,option="H")+
  labs(x = "PC4")
```

```
#Plot centroid size
ggplot(PCA_neuro_neandertal_plotdf_2ei, aes(y = Centroid_size, x=Species))+
  geom_violin(aes(color=Species, fill = Species))+
  scale_colour_viridis_d(option="H")+
  geom_boxplot(width=0.1)+
  scale_fill_viridis_d(alpha=0.3,option="H")+
  labs(x = "Centroid Size")
```

```
#3 Evolutionary models

#Create vector with species assignation
species_neandertal_2ei <- PCA_neuro_neandertal_plotdf_2ei$Species

#load chronology data
chronology_neandertal_2ei <- taxa_chronology|>
  filter(
    !taxon %in% c(
      "early H. sapiens",
      "early H. neanderthalensis",
      "Upper Paleolithic H. sapiens",
      "H. sapiens"
    )
  )

#This function prepares the data to be passed to PaleoTS
PTS_PCS_neandertal_2ei <- dists2PaleoTS(data.frame(PCA_neuro_neandertal_plotdf_2ei[,c(1,3,5:7)]),species_neandertal_2ei,chronology_neandertal_2ei)

#This wrapper function will test the 6 evolutionary models considered in the article against each of the PCs and the centroid size. 
PTS_PCS_models_neandertal_2ei <- multiple_paleoTS(PTS_PCS_neandertal_2ei,models=6)
```

```
Total # hypotheses:  1 
1  

Comparing 6 models [n = 4, method = Joint]

                    logL K      AICc      dAICc Akaike.wt
GRW            2.3163020 3       Inf        Inf     0.000
URW           -3.8040038 2  23.60801  60.570261     0.000
Stasis        -5.7449070 2  27.48981  64.452067     0.000
StrictStasis -44.8493641 1  93.69873 130.660982     0.000
Punc-1        -0.8975948 4 -30.20481   6.757443     0.033
OU             2.4811268 4 -36.96225   0.000000     0.967
```

```
Total # hypotheses:  1 
1  

Comparing 6 models [n = 4, method = Joint]

                   logL K       AICc     dAICc Akaike.wt
GRW          -1.0193833 3        Inf       Inf     0.000
URW          -1.0247568 2  18.049514 48.737202     0.000
Stasis       -0.9573088 2  17.914618 48.602306     0.000
StrictStasis -0.9572687 1   5.914537 36.602226     0.000
Punc-1       -0.9484249 4 -30.103150  0.584538     0.427
OU           -0.6561559 4 -30.687688  0.000000     0.573
```

```
Total # hypotheses:  1 
1  

Comparing 6 models [n = 4, method = Joint]

                   logL K       AICc      dAICc Akaike.wt
GRW          -0.7716038 3        Inf        Inf     0.000
URW          -0.9597941 2  17.919588 48.4105618     0.000
Stasis       -0.9428615 2  17.885723 48.3766965     0.000
StrictStasis -0.9428532 1   5.885706 36.3766799     0.000
Punc-1       -0.8643666 4 -30.271267  0.2197068     0.473
OU           -0.7545132 4 -30.490974  0.0000000     0.527
```

```
Warning in compareModels(grw, urw, sta, strsta, pun2, ou): Optimization for the following model(s) did not converge: OU
These model fit(s) should not be considered reliable.
```

```
Total # hypotheses:  1 
1  

Comparing 6 models [n = 4, method = Joint]

                  logL K      AICc      dAICc Akaike.wt
GRW          -4.766742 3       Inf        Inf     0.000
URW          -7.545961 2  31.09192 55.3007214     0.000
Stasis       -8.207087 2  32.41417 56.6229724     0.000
StrictStasis -8.520134 1  21.04027 45.2490681     0.000
Punc-1       -4.351350 4 -23.29730  0.9114985     0.388
OU           -3.895600 4 -24.20880  0.0000000     0.612
```

#### ii. Facial analyses of the *Homo neanderthalensis* lineage

The analysis follows the same structure as the previous section.

```
#1. Remove specimens that are nots from the H. neanderthalensis lineage
neandertal_lineage_index_2eii <- !face_data$SpeciesExpanded %in% c(
  "early H. sapiens",
  "Upper Paleolithic H. sapiens",
  "H. sapiens"
)

face_neandertal_nomiss_2eii <- face_nomiss|>
  filter( #Filter out species not on H. neanderthalensis lineage
    neandertal_lineage_index_2eii
  )

#2. Calculate PCA
PCA_face_neandertal_2eii <- PCA(face_neandertal_nomiss_2eii[,2:ncol(face_nomiss)],face_neandertal_nomiss_2eii[,1])

#A dataframe is created to facilitate ploting via ggplot
PCA_face_neandertal_plotdf_2eii <- tibble(
  Specimen = face_neandertal_nomiss_2eii$Specimen,
  Species =  face_data$SpeciesExpanded[neandertal_lineage_index_2eii],
  PC1 = PCA_face_neandertal_2eii$PCS[,1],
  PC2 = PCA_face_neandertal_2eii$PCS[,2],
  PC3 = PCA_face_neandertal_2eii$PCS[,3],
  PC4 = PCA_face_neandertal_2eii$PCS[,4],
  Centroid_size = face_gpa$Csize[neandertal_lineage_index_2eii]
)

#Group H. neanderthalensis specimens in one OTU.
PCA_face_neandertal_plotdf_2eii <- PCA_face_neandertal_plotdf_2eii|>
  mutate(
    Species = ifelse(Species == "early H. neanderthalensis", 
                     "H. neanderthalensis",
                     Species),
    Species = factor(Species, levels = c(
      "early Homo",
      "H. erectus",
      "H. heidelbergensis s.l.",
      "H. neanderthalensis"
    )
    )
  
)


#Plot PC1
ggplot(PCA_face_neandertal_plotdf_2eii, aes(y = PC1,x=Species))+
  geom_violin(aes(color=Species, fill = Species))+
  scale_colour_viridis_d(option="H")+
  geom_boxplot(width=0.1)+
  scale_fill_viridis_d(alpha=0.3,option="H")+
  labs(x = "PC1")
```

```
#Plot PC2
ggplot(PCA_face_neandertal_plotdf_2eii, aes(y = PC2,x=Species))+
  geom_violin(aes(color=Species, fill = Species))+
  scale_colour_viridis_d(option="H")+
  geom_boxplot(width=0.1)+
  scale_fill_viridis_d(alpha=0.3,option="H")+
  labs(x = "PC2")
```

```
#Plot PC3
ggplot(PCA_face_neandertal_plotdf_2eii, aes(y = PC3,x=Species))+
  geom_violin(aes(color=Species, fill = Species))+
  scale_colour_viridis_d(option="H")+
  geom_boxplot(width=0.1)+
  scale_fill_viridis_d(alpha=0.3,option="H")+
  labs(x = "PC3")
```

```
#Plot PC4
ggplot(PCA_face_neandertal_plotdf_2eii, aes(y = PC4,x=Species))+
  geom_violin(aes(color=Species, fill = Species))+
  scale_colour_viridis_d(option="H")+
  geom_boxplot(width=0.1)+
  scale_fill_viridis_d(alpha=0.3,option="H")+
  labs(x = "PC4")
```

```
#Plot centroid size
ggplot(PCA_face_neandertal_plotdf_2eii, aes(y = Centroid_size, x=Species))+
  geom_violin(aes(color=Species, fill = Species))+
  scale_colour_viridis_d(option="H")+
  geom_boxplot(width=0.1)+
  scale_fill_viridis_d(alpha=0.3,option="H")+
  labs(x = "Centroid Size")
```

```
#3 Evolutionary models

#Create vector with species assignation
species_neandertal_2eii <- PCA_face_neandertal_plotdf_2eii$Species

#load chronology data
chronology_neandertal_2eii <- taxa_chronology|>
  filter(
    !taxon %in% c(
      "early H. sapiens",
      "early H. neanderthalensis",
      "Upper Paleolithic H. sapiens",
      "H. sapiens"
    )
  )

#This function prepares the data to be passed to PaleoTS
PTS_PCS_neandertal_2eii <- dists2PaleoTS(data.frame(PCA_face_neandertal_plotdf_2eii[,c(1,4:7)]),species_neandertal_2eii,chronology_neandertal_2eii)

#This wrapper function will run test the 6 evolutionary models considered in the article against each of the PCs and the centroid size. 
PTS_PCS_models_neandertal_2eii <- multiple_paleoTS(PTS_PCS_neandertal_2eii,models=6)
```

```
Total # hypotheses:  1 
1  

Comparing 6 models [n = 4, method = Joint]

                    logL K      AICc     dAICc Akaike.wt
GRW            1.3252688 3       Inf       Inf     0.000
URW           -3.2477926 2  22.49559 57.522588     0.000
Stasis        -5.5605913 2  27.12118 62.148186     0.000
StrictStasis -21.7291839 1  47.45837 82.485371     0.000
Punc-1         0.9957443 4 -33.99149  1.035515     0.373
OU             1.5135016 4 -35.02700  0.000000     0.627
```

```
Total # hypotheses:  1 
1  

Comparing 6 models [n = 4, method = Joint]

                  logL K       AICc      dAICc Akaike.wt
GRW          -2.031364 3        Inf        Inf     0.000
URW          -2.102305 2  20.204610 48.8841417     0.000
Stasis       -2.085367 2  20.170734 48.8502655     0.000
StrictStasis -2.085363 1   8.170726 36.8502572     0.000
Punc-1       -1.660234 4 -28.679532  0.0000000     0.532
OU           -1.787486 4 -28.425028  0.2545031     0.468
```

```
Total # hypotheses:  1 
1  

Comparing 6 models [n = 4, method = Joint]

                  logL K      AICc     dAICc Akaike.wt
GRW          -1.925642 3       Inf       Inf     0.000
URW          -4.388012 2  24.77602 54.259771     0.000
Stasis       -4.319194 2  24.63839 54.122134     0.000
StrictStasis -7.320863 1  18.64173 48.125473     0.000
Punc-1       -3.041101 4 -25.91780  3.565948     0.144
OU           -1.258127 4 -29.48375  0.000000     0.856
```

```
Warning in compareModels(grw, urw, sta, strsta, pun2, ou): Optimization for the following model(s) did not converge: GRW
These model fit(s) should not be considered reliable.
```

```
Total # hypotheses:  1 
1  

Comparing 6 models [n = 4, method = Joint]

                  logL K      AICc     dAICc Akaike.wt
GRW          -5.036322 3       Inf       Inf     0.000
URW          -5.349898 2  26.69980 50.247102     0.000
Stasis       -5.340369 2  26.68074 50.228046     0.000
StrictStasis -5.340365 1  14.68073 38.228037     0.000
Punc-1       -4.568786 4 -22.86243  0.684879     0.415
OU           -4.226347 4 -23.54731  0.000000     0.585
```
