# Supplementary material for: Evolutionary drivers of encephalization and facial reduction in the genus Homo
Source: Nat Commun. 2026 Jul 6;17:5625. doi: 10.1038/s41467-026-74739-w (PMC13338430; doi:10.1038/s41467-026-74739-w)
Supplement: Supplementary file 4 — Reporting Summary [file 41467_2026_74739_MOESM4_ESM.pdf]

nature portfolio

Corresponding author(s): Katerina Harvati

Last updated by author(s): Mar 16, 2025

Reporting Summary

Nature Portfolio wishes to improve the reproducibility of the work that we publish. This form provides structure for consistency and transparency in reporting. For further information on Nature Portfolio policies, see our Editorial Policies and the Editorial Policy Checklist.

Statistics

For all statistical analyses, confirm that the following items are present in the figure legend, table legend, main text, or Methods section.

n/a	Confirmed	
☐	☒	The exact sample size (*n*) for each experimental group/condition, given as a discrete number and unit of measurement
☐	☒	A statement on whether measurements were taken from distinct samples or whether the same sample was measured repeatedly
☒	☐	The statistical test(s) used AND whether they are one- or two-sided<br>*Only common tests should be described solely by name; describe more complex techniques in the Methods section.*
☒	☐	A description of all covariates tested
☒	☐	A description of any assumptions or corrections, such as tests of normality and adjustment for multiple comparisons
☐	☒	A full description of the statistical parameters including central tendency (e.g. means) or other basic estimates (e.g. regression coefficient) AND variation (e.g. standard deviation) or associated estimates of uncertainty (e.g. confidence intervals)
☐	☒	For null hypothesis testing, the test statistic (e.g. *F*, *t*, *r*) with confidence intervals, effect sizes, degrees of freedom and *P* value noted<br>*Give P values as exact values whenever suitable.*
☒	☐	For Bayesian analysis, information on the choice of priors and Markov chain Monte Carlo settings
☒	☐	For hierarchical and complex designs, identification of the appropriate level for tests and full reporting of outcomes
☒	☐	Estimates of effect sizes (e.g. Cohen's *d*, Pearson's *r*), indicating how they were calculated

Our web collection on statistics for biologists contains articles on many of the points above.

Software and code

Policy information about availability of computer code

Data collection	Coordinate data were collected by KH using either a microscribe directly from specimens or digitally from 3D models
Data analysis	All analyses were done in R 4.5.2. All packages used in the analyses are cited in the manuscript. The full analysis walkthrough is shared as html and Quarto documents, which include direct links to all additional functions deposited in github.

For manuscripts utilizing custom algorithms or software that are central to the research but not yet described in published literature, software must be made available to editors and reviewers. We strongly encourage code deposition in a community repository (e.g. GitHub). See the Nature Portfolio guidelines for submitting code & software for further information.

Data

Policy information about availability of data

All manuscripts must include a data availability statement. This statement should provide the following information, where applicable:

- Accession codes, unique identifiers, or web links for publicly available datasets
- A description of any restrictions on data availability
- For clinical datasets or third party data, please ensure that the statement adheres to our policy

The neurocranial and facial datasets used in this study have been deposited in Zenodo and are available in https://doi.org/10.5281/zenodo.20049310

Research involving human participants, their data, or biological material

Policy information about studies with human participants or human data. See also policy information about sex, gender (identity/presentation), and sexual orientation and race, ethnicity and racism.

Reporting on sex and gender	n/a
Reporting on race, ethnicity, or other socially relevant groupings	n/a
Population characteristics	n/a
Recruitment	n/a
Ethics oversight	n/a

Note that full information on the approval of the study protocol must also be provided in the manuscript.

Field-specific reporting

Please select the one below that is the best fit for your research. If you are not sure, read the appropriate sections before making your selection.

☐ Life sciences ☐ Behavioural & social sciences ☒ Ecological, evolutionary & environmental sciences

For a reference copy of the document with all sections, see nature.com/documents/nr-reporting-summary-flat.pdf

Ecological, evolutionary & environmental sciences study design

All studies must disclose on these points even when the disclosure is negative.

Study description	The study tests the commonly assumed hypothesis that gradual directional selection drove increased encephalization and reduced facial size of the genus Homo through time by exploring the goodness-of-fit of the predictions of six evolutionary processes to the observed cranial morphological variation in Homo
Research sample	Our sample includes 62 fossil Homo and 24 recent Homo sapiens crania, grouped into eight Operational Taxonomic Units (OTUs), detailed in Table S1 of the manuscript.
Sampling strategy	Sample size is based on the availability of fossil specimens. All specimens measured by KH that had the minimal number of anatomical landmarks for the study were included in the analyses. This is the largest available dataset of early and late Homo specimens currently available. The 24 individuals represent one male and one female randomly chosen from a larger sample of 233 individuals from 12 modern human populations worldwide, representing subsets of previously published samples. This sample size was chosen to avoid the overrepresentation of modern humans in calculation of the average covariance between landmarks
Data collection	Coordinate data were collected by KH using either a microscribe directly from specimens or digitally from 3D models. Landmarks were selected to represent overall craniofacial morphology, while minimizing missing information in the data (see below). The final datasets comprised 21 landmarks for the neurocranial and 23 landmarks for the facial datasets as subsets of previously published data. Tables S2 and S3 list the landmarks included in each dataset, as well as the percentage of missing data for each landmark.
Timing and spatial scale	Morphometric data has been collected over the last two decades, following protocols established by KH. All anatomical landmarks used in the study are well defined in the anatomical literature, widely used, and
Data exclusions	Specimens were excluded based on the availability of anatomical landmarks. Missing data estimation and inclusion criteria are detailed in Material and Methods section.
Reproducibility	No experiments are associated with this study.
Randomization	To maximize the number of steps in the Homo evolutionary lineage in the tests of evolutionary scenarios, we classified the specimens into eight Operational Taxonomic Units (OTUs) along chronological and species boundaries (Table S6). Further details are provided in Materials and Methods.
Blinding	Blinding was not possible during measurements, as the analysis as collection of 3D anatomical landmarks requires the observation of the full hominin skull.

Did the study involve field work? ☐ Yes ☒ No

Reporting for specific materials, systems and methods

We require information from authors about some types of materials, experimental systems and methods used in many studies. Here, indicate whether each material, system or method listed is relevant to your study. If you are not sure if a list item applies to your research, read the appropriate section before selecting a response.

Materials & experimental systems

n/a	Involved in the study
☒	☐ Antibodies
☒	☐ Eukaryotic cell lines
☐	☒ Palaeontology and archaeology
☒	☐ Animals and other organisms
☒	☐ Clinical data
☒	☐ Dual use research of concern
☒	☐ Plants

Methods

n/a	Involved in the study
☒	☐ ChIP-seq
☒	☐ Flow cytometry
☒	☐ MRI-based neuroimaging

Palaeontology and Archaeology

Specimen provenance: Bibliographical references for each of the fossils measured in the study that detail their provenance are provided in Table S1.

Specimen deposition: No new fossils were included in this study.

Dating methods: References supporting the chronology for each fossil are provided in Table S1

☐ Tick this box to confirm that the raw and calibrated dates are available in the paper or in Supplementary Information.

Ethics oversight: No ethical approval was required for this study. Access to each specimen was given by the curators and institutions housing the specimens.

Note that full information on the approval of the study protocol must also be provided in the manuscript.

Plants

Seed stocks: n/a

Novel plant genotypes: n/a

Authentication: n/a
