# Supplementary material for: Evolutionary drivers of encephalization and facial reduction in the genus Homo
Source: Nat Commun. 2026 Jul 6;17:5625. doi: 10.1038/s41467-026-74739-w (PMC13338430; doi:10.1038/s41467-026-74739-w)
Supplement: Supplementary file 5 — Transparent Peer Review file [file 41467_2026_74739_MOESM5_ESM.pdf]

nature portfolio

Peer Review File

Evolutionary drivers of encephalization and facial reduction in the genus Homo

Corresponding Author: Professor Katerina Harvati

This file contains all reviewer reports in order by version, followed by all author rebuttals in order by version.

Version 0:

Reviewer comments:

Reviewer #1

(Remarks to the Author)
The authors present a thought-provoking study using evolutionary model expectations based on changes in means and variances of a morphological time series to compare the relative strength of six evolutionary models against empirical data to test the hypothesis that encephalization and facial size reduction in the genus Homo was driven primarily by directional selection. The authors find that models of stochastic evolution and/or stabilizing selection best explain the pattern of neurocranial and facial shape changes over time, and they infer that this indicates a lifting of constraints on cranial shape in the evolution of the genus Homo, which is consistent with an increased role of cultural factors, such as improved food processing technology, on cranial evolution.
In general, I liked this study, and I appreciate the testing of a multiplicity of evolutionary models to explain a morphological trend in human evolution (i.e. bigger brains and smaller faces) that is routinely assumed to be the result of directional selection. As such, the results make an important contribution to the growing literature on the importance of stochastic and non-directional sources of selection on the evolution of so-called “unique” human phenotypic features. The dataset is impressive, despite being small by the standards of extant taxon studies, and the overall results are clearly reported. I did, however, find that the description of the methodological approach was a bit thin and confusing at times. As such, my biggest concern with the study as currently presented is that it would be difficult to replicate without referencing the cited studies by Hunt and/or becoming familiar with the PaleoTS package in R. I recommend that the authors devote some space to elaborating on the way in which this model fitting works either in the main text and/or Supplementary Information.
Specifically, while I understand the basic differences in the expectations of the six different models tested (as listed in the methods section), the relationship between the parameters needed to test the models and the data provided is not clear. If I am understanding correctly, each specimen provides a set of four principal component scores (derived from the morphometric analysis) and is associated with a timestamp and is then grouped into an OTU. So, each OTU can be reduced to an average position in time around which means and variances are calculated? How does sampling bias affect this? In the case of early Homo, there are only a couple of specimens available for that OTU, so have the authors considered what effect sampling bias might have on the relative goodness-of-fit of the different models? I think adding some more detail on the expectations of the six tested evolutionary scenarios and explaining how those parameters are estimated based on the observed data would help make this a more impactful roadmap for similar studies in the future.
I also think that the authors need to acknowledge that the model expectations are tested across the entire time series, which would limit the ability of this research design to detect any changes in evolutionary rates or changes in the relative impact of stochastic and selective processes over time. What I mean is that, for example, this method would not be able to discern if the early part of a lineage were characterized by strong directional selection, but later time periods were characterized by strong stabilizing selection. If I have understood the method correctly, this is a limitation of the model-fitting method that needs to be explicitly acknowledged.
Finally, I am not sure if the brief commentary on lifting of constraints and the importance of cultural factors in the evolution of the genus Homo is necessary or justified given the methodology applied here. While I do not disagree with the sentiment that the later part of the evolution of our genus was intimately wrapped up with changes in cognition and social learning, I do not think that this study speaks to this issue in any meaningful way. Given that no explicit analysis of morphological constraint is performed here, I think including discussion of evolvability and constraint is not warranted, beyond the finding that some aspects of later Homo craniofacial evolution is characterized by stabilizing selection.
Minor comments:
Line 37: “derived evolutionary hominin trajectory” is unclear to me – is it the trajectory that is derived or the hominins? Please consider revising for clarity.

Line 64: Typo: General Random Walk
Line 91: Insert “a” between “with” and “clear gap”.
Line 146: Instead of “directional selection was also selected” would “directional selection was also detected” work better?
Line 172, Sentence beginning with “Our limited sample size…”, I’m confused by this statement – do you mean that your dataset would not have permitted Punctuated Equilibrium and OU to be detected, even if they were the best explanations for the observed trends? If that is the case, why even include them as possible explanations in the study design? This should become clearer if more detail on the behind-the-scenes mechanics of the model fitting were provided.
Line 212: Instead of “state of completion” do you mean “state of completeness”?
Line 237: “safe” inference is unclear to me – perhaps “accurate inference”
Line 280: this sentence was also confusing, do you mean something like this: “magnitude and direction of changes in trait averages over time, as a function of the variance observed in each step of a lineage?”
Line 296: Should this read “relatively constant directional selective pull…”?

Reviewer #2

(Remarks to the Author)
Thank you for the opportunity to review the manuscript "Evolutionary drivers of encephalization and facial reduction in the genus Homo." The idea of testing key evolutionary scenarios related to brain size and facial size in the genus Homo is a topic worthy of investigation. The application of quantitative genetics methods to the human fossil record is a valid approach to this issue.

However, I see some flaws in the methodology that make it challenging to assess whether the conclusions follow from the analyses as described below.

Major Issues
Construction of the OTUs.
Lumping H. habilis and H. rudolfensis together is theoretically problematic given the specific hypotheses being tested. The criteria used to distinguish these species relate to brain size and (roughly speaking) robusticity of the masticatory system, which relate to the variables being tested. Combining them in a single OUT ‘smears’ the differences between them and creates a non-biologically informed chimera. I am also puzzled by the decision to lump all H. erectus and all H. heidelbergensis while simultaneously separating the early and late Neanderthals and the early, UP and recent H. sapiens into separate OTUs.
The proffered justification relates to differences in fossil availability, but is not supportable from a methodological perspective. The OTUs should reflect broadly similar taxonomic levels, whereas the current configuration has OTUs that incorporate multiple species (Early Homo), multiple potential subspecies or even species (H. erectus sl, H. heidelbergensis sl) or tightly constrained temporal subsets of a single species (H. sapiens, Neanderthals). The OTUs need to be re-worked to be as comparable as possible. For example, many workers believe the east / southeast Asian H. erectus were not directly on the path of H. sapiens or H. neanderthalensis evolution. Perhaps they could be excluded in favor of a more restricted Afro-Georgian sample. However, there is at least as much time depth even in this group as in the Neanderthal and modern human samples, so this may require further refinement.
Related question is why you excluded African HH from the Neanderthal tests. Does this assume that this group was not on this lineage at all?

Variables
The raw variables used in hypothesis testing / model fitting are PC scores from shape coordinates. Because of the scaling step during GPA, these PCs capture shape variation but not size differences directly. This is an important distinction because the hypotheses relate to encephalization (or larger brain size, though these two terms are not interchangeable) and facial reduction, which are inherently related to size. Encephalization is specifically defined as brain size relative to body size, and body size is not considered here. Facial reduction is often conceptualized as relative to overall cranial size as well, and, again, this is not evaluated. Even if these were redefined to be absolute size, these would not correspond to shape variables captured by PC scores. For example, different shapes can correspond to the same brain size (indeed, that is essentially what the authors argue in the Discussion – Neanderthals and H. sapiens undergo brain expansion along distinct shape axes). The paper could be reframed to focus on shape differences. For example, I could imagine relating face shape to evolutionary predictions via biomechanical structural inferences (though perhaps complex), but even less straightforward for the vault. Alternatively, tests could be performed on centroid size or some kind of proportional size measure, although this might require different statistical tests.
The authors presumably recognize this issue as there is no discussion of what many of the higher PCs represent with regard to the hypotheses being tested. Nor is the any discussion of how PC scores do or do not relate to size. There is some acknowledgement of this issue on Lines 166-175, but overall I think that the variables as currently constructed do not provide a clearcut test of the evolutionary scenarios described.

Other issues:
Sampling
Why were H. floresiensis and H. naledi excluded from the analysis? My guess is that it is because the model testing assumes all species are on a single lineage, whereas these species are viewed as side branches (alluded to in the Introduction). However, they could also be includes as additional endpoints alongside Neanderthals and modern humans.
Why was only S17 included of the Asian fossils? (Presumably because it has a preserved face, but OH 9 is included which

does not have a face, which begs the question why so many well-preserved neurocranial fossils from Asia were not included.)
Introduction
Lines 43-44: What is “isotonic directional selection”? This is not a term that I’ve seen before.
Line 64: “Radom” should be “Random”
Line 67: Explain how the OU model differs from the GRW model as these are both directional selection, presumably toward an adaptive peak. It’s my understanding that the OU model involves some stabilizing selection, too, but this need to be clearer.
Results
Lines 75-78: “The PCA of the neurocranial dataset for the H. sapiens lineage captured changes in neurocranial shape related to encephalization in the first two PCs (Figure 1). PC1 (28.2% of variance; Table S4) reflected overall neurocranial expansion in relative cranial height, especially at bregma, as well as in cranial breadth, occurring in a relatively linear trend through time.” While this may be true, there is no evidence presented regarding whether these shapes reflect encephalization (or even absolute brain size). This makes it difficult to interpret.
Methods
Lines 236-243 - Thank you for including information about missing landmarks. How did you land on 35%? 30% still seems like a high percentage. However, this is not the full story – if the 30% are all from the maxilla, for example, is that a ‘legitimate’ reconstruction? Please discuss any problematic or concerning fossils that required a high percentage of missing landmark reconstruction.
Lines 288-314: Please provide more details about the model construction with information about how models were parameterized. For example, the OU model has numerous parameters including the alpha parameter determining the strength of the ‘rubber band.’ These seem directly relevant for understanding the models tested and the interpretation of the results. This is also necessary for reproducability of the study.
Lines 294-296 – The description of the GRW model does not correspond well to Hunt’s description. He calls this a biased walk (Hunt 2006), where a positive mean value for the step distribution produces an increase in the average trait value and a negative mean value produces an average decrease in trait values. It is the case that step distributions with higher variances lead to larger excursions / more volatility. While diversifying selection also increases variance in a population, the mean values will show a bimodal distribution. Is this also the case for the GRW model with a high variance for the step distribution? I’m not sure, but I think that this requires more detail and explanation of how this model was parameterized and how this corresponds to expectations under diversifying selection. The description on Lines 65 “gradual directional selection” seems more appropriate.
Lines 251-257: “…which require the 3D morphometric data to be converted into linear dimensions…The choice of using Principal Components as linear measurements of morphological trends…” Once the GPA is performed (mentioned in the previous paragraph), these are no longer linear dimensions as broadly understood since all landmark configurations have been scaled to unit centroid size. (Also, from a purely semantic perspective, PCs are not “linear measurements” but rather linear combinations of the original variables with weights (coefficients) forming the eigenvectors, but also, it is actually the PC scores that are serving as variables in this analysis.
Figures: The wireframes for the neurocranium are oriented such that the are not particularly interpretable. I assume that glabella is shown to the far left at the bottom of the left-most images, but this should be rotated to approximate Frankfort Horizontal, tipping glabella up to be more co-planar with lambda.
Table S1: What specimens are “Dmanisi 1” and “Dmanini 2” (also, note typo in this spelling)
Why are there references missing for some of the Homo erectus and UP H. sapiens fossils in Table S1? Add information to Table S1 about data source (original fossil, cast, 3D scan, reconstruction, etc.).

Reviewer #3

(Remarks to the Author)
The goal of the study is to evaluate how important different evolutionary processes were to producing changes in relative brain and facial size from early Homo to recent humans and Neanderthals. The authors conclude that there is limited support for directional natural selection and stronger support for neutral evolution and stabilizing natural selection. This is a very interesting study; the analyses are well conceived and executed and the writing is clear. I only have two major (overarching) comments and a few minor comments for the authors to address.

Major comments:

Because the landmark coordinates have been turned into shape variables through Procrustes superimposition, the analyses don’t evaluate trends in absolute cranial capacity or facial size. I agree with the authors that this seems appropriate because, for example, it is generally assumed that when cranial capacity increases the face stays the same size (or decreases in size), which would then produce relative size (shape) changes. That said, I think it would be good for the authors to explain in more detail why analyses of shape are the most appropriate way to evaluate the different models.

The authors should be careful when discussing the analyses to not use “strongest fit” or “goodness of fit” interchangeably with “best supported model” because AIC evaluates both model fit and model complexity. Better fitting models (as measured by log likelihood) may not be preferred if they are more complex (as measured by K) than worse fitting models. Related to this point, in some cases, the General Random Walk model has a higher log likelihood than the Unbiased Random Walk model (e.g., Neurocranial Analysis - Homo sapiens lineage PC1) but the AIC is higher because the General Random Walk model has more parameters than the Unbiased Random Walk model. The simpler model (Unbiased Random Walk) is preferred even though the more complex model (General Random Walk) fits better. This observation potentially reconciles

the clearly observable morphological trends along some of the principal components with the best supported model often being Unbiased Random Walk. In other words, although there may be evidence of directional selection in the presence of directional trends in morphology, this possible evidence isn't strong enough to outweigh the additional complexity that positing direction selection requires relative to positing neutral evolution. It would be good for the authors to incorporate this kind of nuance into their text.

Minor comments:

pp. 1, line 29. There are purported Homo fossils older than 2.5 Ma (Ledi-Geraru, Ethiopia, dated to ~2.8 Ma). Given this, it might be better to phrase this sentence as "The appearance of the genus Homo around 2.5-3.0 million years ago . . ."

pp. 1, line 33. The authors' phrase "increased locomotion efficiency" may be interpreted to mean that hominins became more efficient in all kinds of locomotion, but they actually became more efficient at some kinds of locomotion (bipedalism) but less efficient at other kinds of locomotion (climbing). I would be good to rephrase to make this clearer.

pp. 2, line 64. Typo: "Random"

Version 1:

Reviewer comments:

Reviewer #1

(Remarks to the Author)
The authors have done a good job addressing all of my concerns and questions raised during the initial review of this study. I found the revised manuscript to be much clearer in terms of the description of the models and the interpretation of the results. I also appreciate the inclusion of alternative scenarios where early Homo is represented by just H. habilis or H. rudolfensis etc. and agree that the overall conclusions are robust even in the face of these alternatives.
I find this version of the manuscript to be suitable for publication and have only a few suggestions for minor changes that the authors may wish to consider. The revised manuscript did not include line numbers, so I refer to the page numbers of the compiled PDF that includes the cover letter, reply to reviewers, and where the revised manuscript begins on page 11.
Page 11, first paragraph of Introduction: Consider rephrasing to "increased bipedal locomotor efficiency"
Page 13, first paragraph titled "Encephalization". I found the sentence starting "However, once the models' complexities…" a little confusing. Consider rephrasing as "However, once model complexity is taken into consideration, the Akaike weights show…"
Page 13, near bottom of last paragraph, correct type "into a single OUT"
Page 15, near end of third paragraph, consider changing to "are associated with changes in absolute cranial size" to emphasize difference with encephalization, which is a ratio of brain size to that expected based on body size.
Page 16, first paragraph, consider deleting the "the" before "neurocranial": "Similar to the neurocranial size, facial size…"
Page 18, near end of main paragraph, consider adding "number" to sentence: "… show a compromise between the bare minimum number of fossils in an OTU…"

Reviewer #2

(Remarks to the Author)
As before, I find the topic worthy as it has a long history in human evolutionary studies but the current study applies a novel test of the evolutionary scenario linking changes in brain and face size to directional selection. Authors are commended for real improvements in the current version of the manuscript. Most of my additional comments are minor rather than major. However, I remain concerned about the use of the term 'encephalization' which is understood as an increase in brain relative to body size. The measures used here DO NOT reflect that ratio. Even with the addition of centroid size analyses, the brain size is not truly measured as the landmark data are ectocranial points also include external topography unrelated to brain size (e.g., mastoid process, suprameatal crest). Despite improvements, this looseness (or really, inaccuracy) in language persists.

p. 12, Sentence beginning "Data were collected in the form of three-dimensional coordinates of osteometric landmarks…" I am not sure what you mean by "adjusting for centroid size". I think you mean that you retain the centroid sizes calculated as a byproduct of GPA for further analysis, but please check the wording on this.

p. 13, First sentence. Be more cautious in stating "…captured changes in neurocranial shape related to encephalization…" As noted in the original review, you are capturing changes in shape after scaling to unit centroid size. Encephalization relates to changes in brain size relative to body size…this is not evaluated at all here. You could say "captured changes in neurocranial shape typically seen in species with absolutely larger brain sizes." Or you could state that changes along PC 1 are positively correlated with centroid size and thus are associated with larger cranial size (if true). Or something along those lines. The danger here is that fossils which have smaller brains but more 'rounded' neurocranial shapes that might be interpreted as looking 'encephalized', but that are not particularly large-brained…

p. 13, Results. Presenting both log likelihoods and Akaike results makes for a confusing read (ie., GRW strongly supported by log likelihood but not by Akaike). I recommend you report both in a table, but only discuss those you think are the most

‘correct.’ If you think the Akaike weights are statistically justified, then that is what you should summarize in the Results (as you do for some PCs but not others).

p. 13, last sentence: Homo rudolphensis should be Homo rudolfensis. (also, you can probably just use H. habilis and H. rudolfensis without writing out Homo).
p. 14, First sentence. Similar to earlier comment about being cautious in language describing shape change. Yes, reduced prognathism and more vertical faces are associated with gracilization / reduction of the face. However, this could also describe changes between Australopithecus and Paranthropus, but the latter would not be described as having facial reduction. I think describing the shape changes, and stating that these are changes typically associated facial reduction is more accurate. Or, again, you could correlate PC1 scores with centroid size to see if these shape changes do in fact correlate with smaller overall size of the face.

P. 14: Descriptions of face changes on PCs are missing some key changes related to upper face shape and dental arcade length that appear to be substantial.

p. 16-17, Discussion. Another possible point of discussion is whether the landmark set fully captures the variables under discussion (aside from the issue of scaling by body size), or more accurately, whether it captures more than just brain expansion. For example, quite a few of the landmarks on the neurocranium capture ectocranial topography not clearly related to brain size but rather features extending away from the vault surface (e.g., auriculare, mastoidale, lat. glenoid).
p., Discussion of ‘release of stabilizing selection.’ I find this discussion thought provoking but it would be great to go a little further. What are the genetic / phenotypic expectations in the case of a ‘release of stabilizing selection?’ Theoretically I imagine that an increase in variation may result – do you see evidence for this (or are there sufficient samples to examine this) at these key transitions? Are there other hallmarks that can be used to evaluate this idea? In other words, can this hypothesis be operationalized into a test? It would be great to move this from ‘just so’ story to a true evolutionary model that can be evaluated.

Table S1: Were any virtual reconstructions used? If so, please add this information to the table. For example, I am aware of reconstructions for Dmanisi 2282, KNM-WT 15000, Arago 21, Ndutu, Saccopastore 1?

Reviewer #3

(Remarks to the Author)
The authors have been diligent in addressing my comments and those of the other reviewers.

My only minor comment is that in the text the authors sometimes use “geometric mean” when I believe they mean “centroid size”.

Open Access This Peer Review File is licensed under a Creative Commons Attribution 4.0 International License, which permits use, sharing, adaptation, distribution and reproduction in any medium or format, as long as you give appropriate credit to the original author(s) and the source, provide a link to the Creative Commons license, and indicate if changes were made.

In cases where reviewers are anonymous, credit should be given to 'Anonymous Referee' and the source.
The images or other third party material in this Peer Review File are included in the article's Creative Commons license, unless indicated otherwise in a credit line to the material. If material is not included in the article's Creative Commons license and your intended use is not permitted by statutory regulation or exceeds the permitted use, you will need to obtain permission directly from the copyright holder.
To view a copy of this license, visit https://creativecommons.org/licenses/by/4.0/

REPLY TO THE REVIEWERS

Below we detail how the revised version of the manuscript addresses and incorporates the suggestions, critique, and comments from the three peer reviewers. We are thankful for the comments, ideas, and challenges posed by the reviewers, and we believe the revised manuscript is stronger and clearer thanks to their input.

Reviewer #1 (Remarks to the Author):

REVIEWER: The authors present a thought-provoking study using evolutionary model expectations based on changes in means and variances of a morphological time series to compare the relative strength of six evolutionary models against empirical data to test the hypothesis that encephalization and facial size reduction in the genus Homo was driven primarily by directional selection. The authors find that models of stochastic evolution and/or stabilizing selection best explain the pattern of neurocranial and facial shape changes over time, and they infer that this indicates a lifting of constraints on cranial shape in the evolution of the genus Homo, which is consistent with an increased role of cultural factors, such as improved food processing technology, on cranial evolution.
In general, I liked this study, and I appreciate the testing of a multiplicity of evolutionary models to explain a morphological trend in human evolution (i.e. bigger brains and smaller faces) that is routinely assumed to be the result of directional selection. As such, the results make an important contribution to the growing literature on the importance of stochastic and non-directional sources of selection on the evolution of so-called “unique” human phenotypic features. The dataset is impressive, despite being small by the standards of extant taxon studies, and the overall results are clearly reported. I did, however, find that the description of the methodological approach was a bit thin and confusing at times. As such, my biggest concern with the study as currently presented is that it would be difficult to replicate without referencing the cited studies by Hunt and/or becoming familiar with the PaleoTS package in R. I recommend that the authors devote some space to elaborating on the way in which this model fitting works either in the main text and/or Supplementary Information.

REPLY: The revised Material and Methods were expanded to facilitate the replication of the study, with expanded explanations for Hunt’s models. Please see tracked changes in the revised manuscript. We also have added supplementary documents in html and Quarto format that replicate step by step all the analyses presented in the study. These files include links to github functions written for the analyses. The combination of the expanded description and the explained walkthrough for the analyses makes the study now transparent and replicable.

REVIEWER: Specifically, while I understand the basic differences in the expectations of the six different models tested (as listed in the methods section), the relationship between the parameters needed to test the models and the data provided is not clear. If I am understanding correctly, each specimen provides a set of four principal component scores (derived from the morphometric analysis) and is associated with a timestamp and is then grouped into an OTU. So, each OTU can be reduced to an average position in time around which means and variances are calculated? How does sampling bias affect this? In the case of early Homo, there are only a couple of specimens available for that OTU, so have the authors considered what effect sampling bias might have on the relative goodness-of-fit of the different models? I think adding some more detail on the expectations of the six tested evolutionary scenarios and

explaining how those parameters are estimated based on the observed data would help make this a more impactful roadmap for similar studies in the future.

REPLY: The reviewer is correct that each OTU is collapsed into a mean and variance is estimated from the sample, and that small samples will bias the estimation of variance. To address the magnitude of the impact of sample size and OTU composition in our analyses, we ran five alternative scenarios to see whether inclusion of early *Homo* specimens or the separation of neandertals into early and late specimens had a significant impact on the results. These alternative scenarios are presented as supplementary results (See Tables S7-S11, and explanation of scenarios in Material and Methods). Overall, the patterns observed in alternative scenarios did not change our main conclusions, adding some degree of confidence to our interpretations.

REVIEWER: I also think that the authors need to acknowledge that the model expectations are tested across the entire time series, which would limit the ability of this research design to detect any changes in evolutionary rates or changes in the relative impact of stochastic and selective processes over time. What I mean is that, for example, this method would not be able to discern if the early part of a lineage were characterized by strong directional selection, but later time periods were characterized by strong stabilizing selection. If I have understood the method correctly, this is a limitation of the model-fitting method that needs to be explicitly acknowledged.

REPLY: We agree with the reviewer. This point was already presented in the original version, but is now made more explicit in the Discussion section. Please see revised discussion.

REVIEWER: Finally, I am not sure if the brief commentary on lifting of constraints and the importance of cultural factors in the evolution of the genus Homo is necessary or justified given the methodology applied here. While I do not disagree with the sentiment that the later part of the evolution of our genus was intimately wrapped up with changes in cognition and social learning, I do not think that this study speaks to this issue in any meaningful way. Given that no explicit analysis of morphological constraint is performed here, I think including discussion of evolvability and constraint is not warranted, beyond the finding that some aspects of later Homo craniofacial evolution is characterized by stabilizing selection.

REPLY: We agree with the reviewer's comment, and have softened our conclusions regarding the causes for the evolutionary patterns supported by the models tested. Please refer to revised discussion.

REVIEWER: Minor comments:
Line 37: "derived evolutionary hominin trajectory" is unclear to me – is it the trajectory that is derived or the hominins? Please consider revising for clarity.
Line 64: Typo: General Random Walk
Line 91: Insert "a" between "with" and "clear gap".
Line 146: Instead of "directional selection was also selected" would "directional selection was also detected" work better?
Line 172, Sentence beginning with "Our limited sample size…", I'm confused by this statement – do you mean that your dataset would not have permitted Punctuated Equilibrium and OU to be detected, even if they were the best explanations for the observed trends? If that is the case,

why even include them as possible explanations in the study design? This should become clearer if more detail on the behind-the-scenes mechanics of the model fitting were provided.
Line 212: Instead of “state of completion” do you mean “state of completeness”?
Line 237: “safe” inference is unclear to me – perhaps “accurate inference”
Line 280: this sentence was also confusing, do you mean something like this: “magnitude and direction of changes in trait averages over time, as a function of the variance observed in each step of a lineage?”
Line 296: Should this read “relatively constant directional selective pull…”?

REPLY: All minor comments were fixed or addressed in response to the reviewer’s comments.

Reviewer #2 (Remarks to the Author):

REVIEWER: Thank you for the opportunity to review the manuscript "Evolutionary drivers of encephalization and facial reduction in the genus Homo." The idea of testing key evolutionary scenarios related to brain size and facial size in the genus Homo is a topic worthy of investigation. The application of quantitative genetics methods to the human fossil record is a valid approach to this issue.

However, I see some flaws in the methodology that make it challenging to assess whether the conclusions follow from the analyses as described below.

Major Issues
Construction of the OTUs.
Lumping H. habilis and H. rudolfensis together is theoretically problematic given the specific hypotheses being tested. The criteria used to distinguish these species relate to brain size and (roughly speaking) robusticity of the masticatory system, which relate to the variables being tested. Combining them in a single OUT ‘smears’ the differences between them and creates a non-biologically informed chimera.

REPLY: While we agree with the reviewer that lumping the two species in one single OTU is problematic, the lack of well-preserved early fossils makes it difficult to establish the morphology of the start of the genus *Homo* lineage. However, while we concur that the early *Homo* OTU creates a chimera that may not represent the typical ancestral morphology of our lineage, we believe that this chimera is still not too divergent from what our ancestral morphology would have been, especially when the differences between the early specimens are seen in relationship to later *Homo* species. To address the concern of the reviewer, we made our rationale for lumping the species more detailed in the revised manuscript (see revised Materials and Methods), and we also present as supplementary results three alternative evolutionary scenarios, one using only *H. habilis* as early *Homo*, one using only *H. rudolphensis*, and one where the lineage starts with *H. erectus* and the early *Homo* specimens are excluded. The results of the alternative scenarios are largely consistent with our initial results, which add confidence to the fact that the lumping of the two early specimens is not biasing the results significantly.

REVIEWER: I am also puzzled by the decision to lump all H. erectus and all H. heidelbergensis while simultaneously separating the early and late Neanderthals and the early, UP and recent H. sapiens into separate OTUs.
The proffered justification relates to differences in fossil availability, but is not supportable from a methodological perspective. The OTUs should reflect broadly similar taxonomic levels, whereas the current configuration has OTUs that incorporate multiple species (Early Homo), multiple potential subspecies or even species (H. erectus sl, H. heidelbergensis sl) or tightly constrained temporal subsets of a single species (H. sapiens, Neanderthals). The OTUs need to be re-worked to be as comparable as possible. For example, many workers believe the east / southeast Asian H. erectus were not directly on the path of H. sapiens or H. neanderthalensis evolution. Perhaps they could be excluded in favor of a more restricted Afro-Georgian sample. However, there is at least as much time depth even in this group as in the Neanderthal and modern human samples, so this may require further refinement.

REPLY: The reviewer is correct in pointing out that the OTUs don't necessarily represent similar taxonomic levels. However, the model fitting methods adopted here do not require that similar taxonomic levels are presented, only that they are part of a lineage with time steps between OTUs defined. The models tested assume that the entire lineage was subject to the same evolutionary process across its entire existence, which means that the expected changes in trait values are proportional to the time that separates consecutive steps in the lineage. As such, OTUs chronologically close to each are expected to show small changes compared to more distantly related steps. This proportional expectation of change is consistent with diachronic trends within a species as well as with changes between species. Parameters that are of larger relevance to the estimation of models' goodness of fit are the mean and variance in each step and the number of steps in the lineage. Therefore, the lineages used in this study show a compromise between the bare minimum of fossils in an OTU to allow a calculation of variance and the maximum number of OTUs that can be considered part of the lineage. To address the concerns of the reviewer, we also ran one alternative scenarios grouping neandertals into a single OTU, and another scenario excluding modern humans from the *H. sapiens* lineage, to make it more directly comparable to the results of the *H. neanderthalensis* in terms of OTUs and approximate sample sizes. The results of the joined Neandertal scenario show support to OU and punctuated equilibrium models, but the fact that there are only 4 OTUs in this analysis makes the results harder to interpret. They do favor models that rely on stasis during part of the linage, and in that sense agree with the general results obtained in the overall analysis. The analysis of the *H. sapiens* lineage without modern humans presents the same general results as the analyses in the main manuscript. We have added this explanation to the revised Material and Methods section.

Finally, we have removed Sangiran 17, and restricted our lineages to African and Europeans fossils.

REVIEWER: Related question is why you excluded African HH from the Neanderthal tests. Does this assume that this group was not on this lineage at all?
REPLY: This was corrected in the revised version and all *H. heidelbergensis* are considered part of the *H. sapiens* and *H. neaderthalensis* lineage, given the inability to confidently assign specific fossils to each lineage.

REVIEWER: Variables
The raw variables used in hypothesis testing / model fitting are PC scores from shape coordinates. Because of the scaling step during GPA, these PCs capture shape variation but not size differences directly. This is an important distinction because the hypotheses relate to encephalization (or larger brain size, though these two terms are not interchangeable) and facial reduction, which are inherently related to size. Encephalization is specifically defined as brain size relative to body size, and body size is not considered here. Facial reduction is often conceptualized as relative to overall cranial size as well, and, again, this is not evaluated. Even if these were redefined to be absolute size, these would not correspond to shape variables captured by PC scores. For example, different shapes can correspond to the same brain size (indeed, that is essentially what the authors argue in the Discussion – Neanderthals and H. sapiens undergo brain expansion along distinct shape axes). The paper could be reframed to focus on shape differences. For example, I could imagine relating face shape to evolutionary predictions via biomechanical structural inferences (though perhaps complex), but even less straightforward for the vault. Alternatively, tests could be performed on centroid size or some kind of proportional size measure, although this might require different statistical tests.

REPLY: To incorporate a dimension of size change in our analyses, the revised manuscript now explores centroid size of neurocrania and face as variables that were tested using the same model-fitting approach. The results were incorporated in the main article analyses and in all the alternative scenarios presented in the supplementary results.

REVIEWER: The authors presumably recognize this issue as there is no discussion of what many of the higher PCs represent with regard to the hypotheses being tested. Nor is the any discussion of how PC scores do or do not relate to size. There is some acknowledgement of this issue on Lines 166-175, but overall I think that the variables as currently constructed do not provide a clearcut test of the evolutionary scenarios described.
REPLY: With the addition of the analysis of centroid size, we incorporated discussion of size changes as they related to neurocranial expansion and facial reduction in the lineage. We maintain, however, that the hypotheses tested in the manuscript relate directly to shape changes in the architecture of neurocraniums and face. We agree with the reviewer that trends in shape changes are not very evident in many of the higher PCs presented, but these PCs still represent a significant portion of the variance in the dataset, and as such represent dimensions of high potential evolvability. We clarified the description of shapes related to our hypotheses throughout the results section.

REVIEWER: Other issues:
Sampling
Why were H. floresiensis and H. naledi excluded from the analysis? My guess is that it is because the model testing assumes all species are on a single lineage, whereas these species are viewed as side branches (alluded to in the Introduction). However, they could also be includes as additional endpoints alongside Neanderthals and modern humans. Why was only S17 included of the Asian fossils? (Presumably because it has a preserved face, but OH 9 is

included which does not have a face, which begs the question why so many well-preserved neurocranial fossils from Asia were not included.)

RESPONSE: The reviewer is correct: naledi and florensiensis were not included as we restricted our analyses to lineages with reasonably accepted ancestorship relationships between OTUs. As mentioned above, Sangiran 17 was removed from the analyses to focus on the African and Eruopean fossil record

REVIEWER: Introduction
Lines 43-44: What is “isotonic directional selection”? This is not a term that I’ve seen before.

REPLY: This term was changed to gradual direction selection and this was used throughout the manuscript.

Line 64: “Radom” should be “Random”
REPLY: fixed.

REVIEWER: Line 67: Explain how the OU model differs from the GRW model as these are both directional selection, presumably toward an adaptive peak. It’s my understanding that the OU model involves some stabilizing selection, too, but this need to be clearer.
REPLY: Expanded in Introduction and Material and Methods. OU assumes that species gravitate around an ideal phenotypic peak for most of their lineage time, and therefore phenotypic change is not gradual (linear) over time. GRW expects that changes fall closer to a linear progression over time without an optimal phenotypic value being reached.

REVIEWER: Results
Lines 75-78: “The PCA of the neurocranial dataset for the H. sapiens lineage captured changes in neurocranial shape related to encephalization in the first two PCs (Figure 1). PC1 (28.2% of variance; Table S4) reflected overall neurocranial expansion in relative cranial height, especially at bregma, as well as in cranial breadth, occurring in a relatively linear trend through time.” While this may be true, there is no evidence presented regarding whether these shapes reflect encephalization (or even absolute brain size). This makes it difficult to interpret.
REPLY: See reply to previous comment, related to the inclusion of geometric mean in the analyses.

REVIEWER: Methods
Lines 236-243 - Thank you for including information about missing landmarks. How did you land on 35%? 30% still seems like a high percentage. However, this is not the full story – if the 30% are all from the maxilla, for example, is that a ‘legitimate’ reconstruction? Please discuss any problematic or concerning fossils that required a high percentage of missing landmark reconstruction.

REPLY: 35% was the best compromise we found after testing for different cut values. The reviewer is right that this is still a high percentage of error, but we note that the average percentage of missing values among the fossils is 8.4% for the face and 3.8% for the neurocranium. So, while it is possible that fossils would be poorly estimated depending on

where the missing values occur, very few of the specimens would be susceptible to this type of bias. This information is now presented in Materials and Methods.

REVIEWER: Lines 288-314: Please provide more details about the model construction with information about how models were parameterized. For example, the OU model has numerous parameters including the alpha parameter determining the strength of the 'rubber band.' These seem directly relevant for understanding the models tested and the interpretation of the results. This is also necessary for reproducability of the study.

REPLY: The parameters included in each model have been detailed in the revised version. Parameters for each are estimated as part of the model-fitting calculations developed by Hunt. To make sure the analyses are reproducible, we added a commented documented in html and quarto formats (SI documents 1 and 2), that presents the complete walkthrough of the analyses, as well as functions and data required for it.

REVIEWER: Lines 294-296 – The description of the GRW model does not correspond well to Hunt's description. He calls this a biased walk (Hunt 2006), where a positive mean value for the step distribution produces an increase in the average trait value and a negative mean value produces an average decrease in trait values. It is the case that step distributions with higher variances lead to larger excursions / more volatility. While diversifying selection also increases variance in a population, the mean values will show a bimodal distribution. Is this also the case for the GRW model with a high variance for the step distribution? I'm not sure, but I think that this requires more detail and explanation of how this model was parameterized and how this corresponds to expectations under diversifying selection. The description on Lines 65 "gradual directional selection" seems more appropriate.

REPLY: We are thankful for the error noted by the reviewer. This has been corrected in the revised version. As remarked by the reviewer, our use of diversifying selection is not accurate, as it is commonly used to explain the split of lineages. We changed the term to gradual directional selection. Under the GRW, bimodal distribution are not expected to be formed, as gradual linear change is expected across the different steps of the lineage.

REVIEWER: Lines 251-257: "…which require the 3D morphometric data to be converted into linear dimensions…The choice of using Principal Components as linear measurements of morphological trends…" Once the GPA is performed (mentioned in the previous paragraph), these are no longer linear dimensions as broadly understood since all landmark configurations have been scaled to unit centroid size. (Also, from a purely semantic perspective, PCs are not "linear measurements" but rather linear combinations of the original variables with weights (coefficients) forming the eigenvectors, but also, it is actually the PC scores that are serving as variables in this analysis.

REPLY: We are thankful for the correction. We fixed the wrong terminology to explain the outcome of the PC analysis.

REVIEWER: Figures: The wireframes for the neurocranium are oriented such that the are not particularly interpretable. I assume that glabella is shown to the far left at the bottom of the left-

most images, but this should be rotated to approximate Frankfort Horizontal, tipping glabella up to be more co-planar with lambda.

REPLY: Fixed.

REVIEWER: Table S1: What specimens are “Dmanisi 1” and “Dmanini 2” (also, note typo in this spelling)
Why are there references missing for some of the Homo erectus and UP H. sapiens fossils in Table S1? Add information to Table S1 about data source (original fossil, cast, 3D scan, reconstruction, etc.).

REPLY: Clarified and fixed.

Reviewer #3 (Remarks to the Author):

REVIEWER: The goal of the study is to evaluate how important different evolutionary processes were to producing changes in relative brain and facial size from early Homo to recent humans and Neanderthals. The authors conclude that there is limited support for directional natural selection and stronger support for neutral evolution and stabilizing natural selection. This is a very interesting study; the analyses are well conceived and executed and the writing is clear. I only have two major (overarching) comments and a few minor comments for the authors to address.

Major comments:

Because the landmark coordinates have been turned into shape variables through Procrustes superimposition, the analyses don’t evaluate trends in absolute cranial capacity or facial size. I agree with the authors that this seems appropriate because, for example, it is generally assumed that when cranial capacity increases the face stays the same size (or decreases in size), which would then produce relative size (shape) changes. That said, I think it would be good for the authors to explain in more detail why analyses of shape are the most appropriate way to evaluate the different models.

REPLY: The use of shape in relationship to the hypotheses tested has been clarified in the revised version. As detailed in the reply to Reviewer 2, we also include the analyses of size change as measured through geometric means of the fossils, to include in the discussion the implications of size changes in the lineage.

REVIEWER: The authors should be careful when discussing the analyses to not use “strongest fit” or “goodness of fit” interchangeably with “best supported model” because AIC evaluates both model fit and model complexity. Better fitting models (as measured by log likelihood) may not be preferred if they are more complex (as measured by K) than worse fitting models. Related to this point, in some cases, the General Random Walk model has a higher log likelihood than the Unbiased Random Walk model (e.g., Neurocranial Analysis - Homo sapiens lineage PC1) but the AIC is higher because the General Random Walk model has more parameters than the Unbiased Random Walk model. The simpler model (Unbiased Random Walk) is preferred even

though the more complex model (General Random Walk) fits better. This observation potentially reconciles the clearly observable morphological trends along some of the principal components with the best supported model often being Unbiased Random Walk. In other words, although there may be evidence of directional selection in the presence of directional trends in morphology, this possible evidence isn't strong enough to outweigh the additional complexity that positing direction selection requires relative to positing neutral evolution. It would be good for the authors to incorporate this kind of nuance into their text.

REPLY: We appreciate the reviewer bringing this point to our attention. We have incorporated these points in our results and revised discussion.

REVIEWER: Minor comments:

pp. 1, line 29. There are purported Homo fossils older than 2.5 Ma (Ledi-Geraru, Ethiopia, dated to ~2.8 Ma). Given this, it might be better to phrase this sentence as "The appearance of the genus Homo around 2.5-3.0 million years ago . . ."

REPLY: Fixed.

REVIEWER: pp. 1, line 33. The authors' phrase "increased locomotion efficiency" may be interpreted to mean that hominins became more efficient in all kinds of locomotion, but they actually became more efficient at some kinds of locomotion (bipedalism) but less efficient at other kinds of locomotion (climbing). I would be good to rephrase to make this clearer.

REPLY: Rephrased.

REVIEWER: pp. 2, line 64. Typo: "Random"

REPLY: Fixed.

REVIEWERS' COMMENTS

Reviewer #1 (Remarks to the Author):

The authors have done a good job addressing all of my concerns and questions raised during the initial review of this study. I found the revised manuscript to be much clearer in terms of the description of the models and the interpretation of the results. I also appreciate the inclusion of alternative scenarios where early Homo is represented by just H. habilis or H. rudolfensis etc. and agree that the overall conclusions are robust even in the face of these alternatives.
I find this version of the manuscript to be suitable for publication and have only a few suggestions for minor changes that the authors may wish to consider. The revised manuscript did not include line numbers, so I refer to the page numbers of the compiled PDF that includes the cover letter, reply to reviewers, and where the revised manuscript begins on page 11.

Page 11, first paragraph of Introduction: Consider rephrasing to "increased bipedal locomotor efficiency"

REPLY: Changed to "increased locomotion efficiency"

Page 13, first paragraph titled "Encephalization". I found the sentence starting "However, once the models' complexities…" a little confusing. Consider rephrasing as "However, once model complexity is taken into consideration, the Akaike weights show…"

REPLY: Results were edited to focus only on Akaike weights, following Reviewer 2 suggestion, which addresses this comment as well.

Page 13, near bottom of last paragraph, correct type "into a single OUT"
REPLY: Fixed

Page 15, near end of third paragraph, consider changing to "are associated with changes in absolute cranial size" to emphasize difference with encephalization, which is a ratio of brain size to that expected based on body size.
REPLY: Suggestions accepted

Page 16, first paragraph, consider deleting the "the" before "neurocranial": "Similar to the neurocranial size, facial size…"

REPLY: Fixed.
Page 18, near end of main paragraph, consider adding “number” to sentence: “… show a compromise between the bare minimum number of fossils in an OTU…”

REPLY: Added.

Reviewer #2 (Remarks to the Author):

As before, I find the topic worthy as it has a long history in human evolutionary studies but the current study applies a novel test of the evolutionary scenario linking changes in brain and face size to directional selection. Authors are commended for real improvements in the current version of the manuscript. Most of my additional comments are minor rather than major. However, I remain concerned about the use of the term 'encephalization' which is understood as an increase in brain relative to body size. The measures used here DO NOT reflect that ratio. Even with the addition of centroid size analyses, the brain size is not truly measured as the landmark data are ectocranial points also include external topography unrelated to brain size (e.g., mastoid process, suprameatal crest). Despite improvements, this looseness (or really, inaccuracy) in language persists.
REPLY: We have now acknowledged the limitations of our study in what regards to our estimation of encephalization and brain morphology in the introduction, methods and discussion. In the introduction and methods, we detail our rationale to assume the analysis of shape and size as proxies for encephalization. In the discussion we explicitly recognize the limitation of our study given that it is limited to ectocranial landmarks.

p. 12, Sentence beginning “Data were collected in the form of three-dimensional coordinates of osteometric landmarks…” I am not sure what you mean by “adjusting for centroid size”. I think you mean that you retain the centroid sizes calculated as a byproduct of GPA for further analysis, but please check the wording on this.

REPLY: Rephrased.

p. 13, First sentence. Be more cautious in stating “…captured changes in neurocranial shape related to encephalization…” As noted in the original review, you are capturing changes in shape after scaling to unit centroid size. Encephalization relates to changes in brain size relative to body size…this is not evaluated at all here. You could say “captured changes in neurocranial shape typically seen in species with absolutely larger brain sizes.” Or you could state that changes along PC 1 are positively correlated with

centroid size and thus are associated with larger cranial size (if true). Or something along those lines. The danger here is that fossils which have smaller brains but more 'rounded' neurocranial shapes that might be interpreted as looking 'encephalized', but that are not particularly large-brained…

REPLY: Rephrased after first suggestion.

p. 13, Results. Presenting both log likelihoods and Akaike results makes for a confusing read (ie., GRW strongly supported by log likelihood but not by Akaike). I recommend you report both in a table, but only discuss those you think are the most 'correct.' If you think the Akaike weights are statistically justified, then that is what you should summarize in the Results (as you do for some PCs but not others).

REPLY: We followed the suggestion of the reviewer, and focused the presentation of Akaike weights.

p. 13, last sentence: Homo rudolphensis should be Homo rudolfensis. (also, you can probably just use H. habilis and H. rudolfensis without writing out Homo).

REPLY: Fixed

p. 14, First sentence. Similar to earlier comment about being cautious in language describing shape change. Yes, reduced prognathism and more vertical faces are associated with gracilization / reduction of the face. However, this could also describe changes between Australopithecus and Paranthropus, but the latter would not be described as having facial reduction. I think describing the shape changes, and stating that these are changes typically associated facial reduction is more accurate. Or, again, you could correlate PC1 scores with centroid size to see if these shape changes do in fact correlate with smaller overall size of the face.

REPLY: We have added a stronger justification of the use of facial shape and size data as proxies to facial reduction in the introduction and methods, together with the discussion about encephalization. We did not include discussion about facial changes in australopiths as our analysis focuses only on the genus *Homo*, which have a derived evolutionary trajectory from robust australopiths.

P. 14: Descriptions of face changes on PCs are missing some key changes related to upper face shape and dental arcade length that appear to be substantial.

REPLY: Descriptions were revised to include these suggestions.

p. 16-17, Discussion. Another possible point of discussion is whether the landmark set

fully captures the variables under discussion (aside from the issue of scaling by body size), or more accurately, whether it captures more than just brain expansion. For example, quite a few of the landmarks on the neurocranium capture ectocranial topography not clearly related to brain size but rather features extending away from the vault surface (e.g., auriculare, mastoidale, lat. glenoid).

REPLY: This limitation is now acknowledged in the discussion.

p., Discussion of 'release of stabilizing selection.' I find this discussion thought provoking but it would be great to go a little further. What are the genetic / phenotypic expectations in the case of a 'release of stabilizing selection?' Theoretically I imagine that an increase in variation may result – do you see evidence for this (or are there sufficient samples to examine this) at these key transitions? Are there other hallmarks that can be used to evaluate this idea? In other words, can this hypothesis be operationalized into a test? It would be great to move this from 'just so' story to a true evolutionary model that can be evaluated.
REPLY: The discussion was expanded a little to acknowledge the point of the reviewer. We acknowledge more explicitly the importance of identifying key moments of release of stabilizing selection and the expected increase in variance that would result from it, but also recognize the limitations of the fossil record that would allow us to test this more formally.

Table S1: Were any virtual reconstructions used? If so, please add this information to the table. For example, I am aware of reconstructions for Dmanisi 2282, KNM-WT 15000, Arago 21, Ndutu, Saccopastore 1?

REPLY: The information has been added to Table S1. The only fossil reconstruction used was from Arago, and a citation for that reconstruction has been added as a footnote.

Reviewer #3 (Remarks to the Author):

The authors have been diligent in addressing my comments and those of the other reviewers.

My only minor comment is that in the text the authors sometimes use "geometric mean" when I believe they mean "centroid size".

REPLY: Fixed all instances.
